# Computational Design of Pore-Forming Peptides with Potent Antimicrobial and Anticancer Activities

**DOI:** 10.1021/acs.jmedchem.4c00912

**Published:** 2024-08-08

**Authors:** Rahul Deb, Marcelo D. T. Torres, Miroslav Boudný, Markéta Koběrská, Floriana Cappiello, Miroslav Popper, Kateřina Dvořáková Bendová, Martina Drabinová, Adelheid Hanáčková, Katy Jeannot, Miloš Petřík, Maria Luisa Mangoni, Gabriela Balíková Novotná, Marek Mráz, Cesar de la Fuente-Nunez, Robert Vácha

**Affiliations:** † CEITEC − Central European Institute of Technology, Masaryk University, Brno 625 00, Czech Republic; ‡ National Centre for Biomolecular Research, Faculty of Science, Masaryk University, Brno 625 00, Czech Republic; § Machine Biology Group, Departments of Psychiatry and Microbiology, Institute for Biomedical Informatics, Institute for Translational Medicine and Therapeutics, Perelman School of Medicine, University of Pennsylvania, Philadelphia, Pennsylvania 19104, United States; ∥ Departments of Bioengineering and Chemical and Biomolecular Engineering, School of Engineering and Applied Science, University of Pennsylvania, Philadelphia, Pennsylvania 19104, United States; ⊥ Penn Institute for Computational Science, University of Pennsylvania, Philadelphia, Pennsylvania 19104, United States; # Department of Chemistry, School of Arts and Sciences, University of Pennsylvania, Philadelphia, Pennsylvania 19104, United States; ∇ Department of Internal Medicine, Hematology and Oncology, University Hospital Brno and Faculty of Medicine, Masaryk University, Brno 625 00, Czech Republic; ○ Institute of Microbiology, Czech Academy of Sciences, BIOCEV, Vestec 252 50, Czech Republic; ◆ Department of Biochemical Sciences, Laboratory Affiliated to Istituto Pasteur Italia-Fondazione Cenci Bolognetti, Sapienza University of Rome, Rome 00185, Italy; ¶ Institute of Molecular and Translational Medicine, Faculty of Medicine and Dentistry, Palacký University, Olomouc 779 00, Czech Republic; & University of Franche-Comté, CNRS, Chrono-environment, Besançon 25030, France; ● National Reference Centre for Antibiotic Resistance, Besançon 25030, France; ◊ Czech Advanced Technology and Research Institute, Palacký University, Olomouc 779 00, Czech Republic; ▲ Department of Condensed Matter Physics, Faculty of Science, Masaryk University, Brno 611 37, Czech Republic

## Abstract

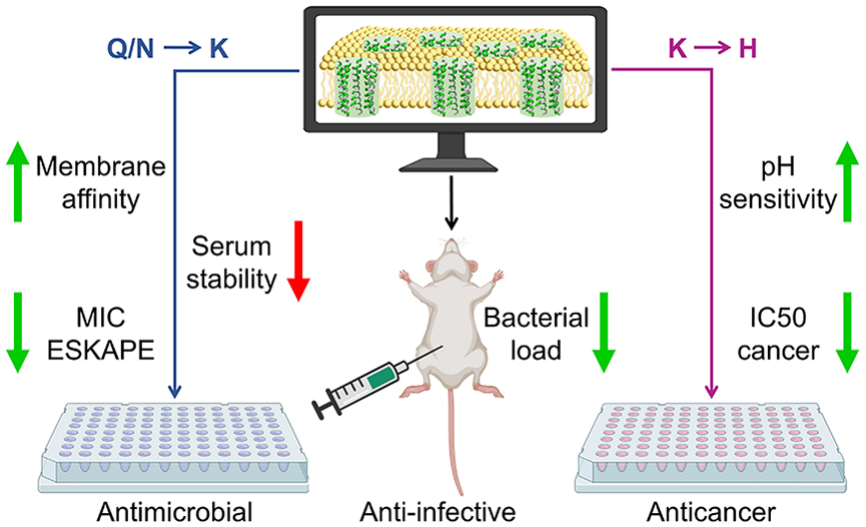

Peptides that form transmembrane barrel-stave pores are potential alternative therapeutics for bacterial infections and cancer. However, their optimization for clinical translation is hampered by a lack of sequence-function understanding. Recently, we have *de novo* designed the first synthetic barrel-stave pore-forming antimicrobial peptide with an identified function of all residues. Here, we systematically mutate the peptide to improve pore-forming ability in anticipation of enhanced activity. Using computer simulations, supported by liposome leakage and atomic force microscopy experiments, we find that pore-forming ability, while critical, is not the limiting factor for improving activity in the submicromolar range. Affinity for bacterial and cancer cell membranes needs to be optimized simultaneously. Optimized peptides more effectively killed antibiotic-resistant ESKAPEE bacteria at submicromolar concentrations, showing low cytotoxicity to human cells and skin model. Peptides showed systemic anti-infective activity in a preclinical mouse model of *Acinetobacter baumannii* infection. We also demonstrate peptide optimization for pH-dependent antimicrobial and anticancer activity.

## Introduction

Two large issues loom over the global public health landscape: antimicrobial resistance^1^ and cancer.^2^ Bacterial resistance to last-resort antibiotics^1,3,4^ and cancer cell resistance to existing anticancer drugs^5,6^ pose serious threats to human health and the global economy.

In particular, the resistance of Gram-negative bacteria to carbapenems, colistin, and aminoglycosides, i.e., the antibiotics of last resort for multidrug-resistant bacterial infections, has created a need for alternative antibiotics.^7,8^ Antimicrobial peptides (AMPs), which can rapidly kill bacteria by forming pores in bacterial cell membranes, have the potential to become a new generation of antibiotics because bacteria do not readily develop resistance to them.^9−17^ However, increasing antimicrobial activity and reducing cytotoxicity while maintaining pore-forming ability requires careful optimization.

Cancer is a leading cause of death worldwide,^2^ and cancer cells are becoming increasingly resistant to current anticancer drugs.^5,6^ Similar to bacterial cell killing activity, pore-forming peptides exhibit direct cytotoxicity against cancer cells and therefore offer an alternative or complementary treatment.^18−22^ However, cytotoxicity should be selective and therefore needs to be carefully optimized.

Electrostatic interactions are thought to determine peptide selectivity, at least in part.^23,24^ Most antimicrobial and anticancer peptides have a net positive charge, while bacterial and cancer cells have negatively charged lipids exposed to the extracellular space. In contrast, normal human cells have mainly zwitterionic lipids in the extracellular space. In addition, the acidic extracellular microenvironment of cancer cells could be used to tune pore-forming AMPs into pH-sensitive anticancer agents that would selectively kill below the physiological pH of normal cells.^25−28^

We recently demonstrated that computer simulation can be useful for the *de novo* design of α-helical peptides that self-assemble into transmembrane barrel-stave pores (TBPs) capable of transporting small molecules across lipid membranes.^29^ We identified the role of amino acids at each position of the 30-residue-long peptides and proposed a set of design guidelines and 52 sequence patterns for TBP-forming peptides. The defined roles of individual residues in the proposed sequence patterns provide control over the pore properties, enabling the custom design of pore-forming peptides for different applications. We demonstrated such fine-tuning for antimicrobial applications; tuned AMPs killed both Gram-negative and Gram-positive bacteria. The most active AMP, KDFA2i+9-NH2, presented minimum inhibitory concentration (MIC) values in the micromolar range against some of the antibiotic-resistant ESKAPEE pathogens^30^ tested and exhibited low toxicity for normal human cells.^29^ Atomic force microscopy (AFM), channel electrical recordings, and fluorescence experiments confirmed that KDFA2i+9-NH2 forms large, stable, and functional TBPs, which is the likely mechanism of antimicrobial action. Notably, KDFA2i+9-NH2 is the first synthetic AMP that has been shown to form TBPs. Therefore, KDFA2i+9-NH2 represents a good scaffold for the systematic investigation of mutations that would improve the peptide activity.

Here, we employed a structure-based investigative approach, specifically aiming to improve intermolecular peptide–peptide interactions to enhance pore stability. Our hypothesis is that peptides capable of forming more stable pores would be more effective in disrupting the membrane barrier and killing bacteria. However, the extent to which this activity can be improved by solely focusing on pore stability remains unclear, as several other factors are involved.^9,10,23,31^ Therefore, we first performed molecular dynamics (MD) simulations with 46 mutants to identify the mutations that confer greater stability to TBPs. The peptides were tested *in vitro* for their ability to permeabilize membranes and kill bacterial and human cells. We then evaluated the relationships between simulated pore-stabilizing activity, on the one hand, and experimental pore-forming activity, antimicrobial activity against ESKAPEE pathogens, toxicity for human and murine cells, and effects in a human skin model, on the other hand. We demonstrated the anti-infective properties using preclinical mouse models of Gram-negative bacterial infection. Furthermore, we investigated the optimization for pH-dependent antimicrobial activity and anticancer application of the peptides.

## Results

### Computer Simulations

**Figure 1:**
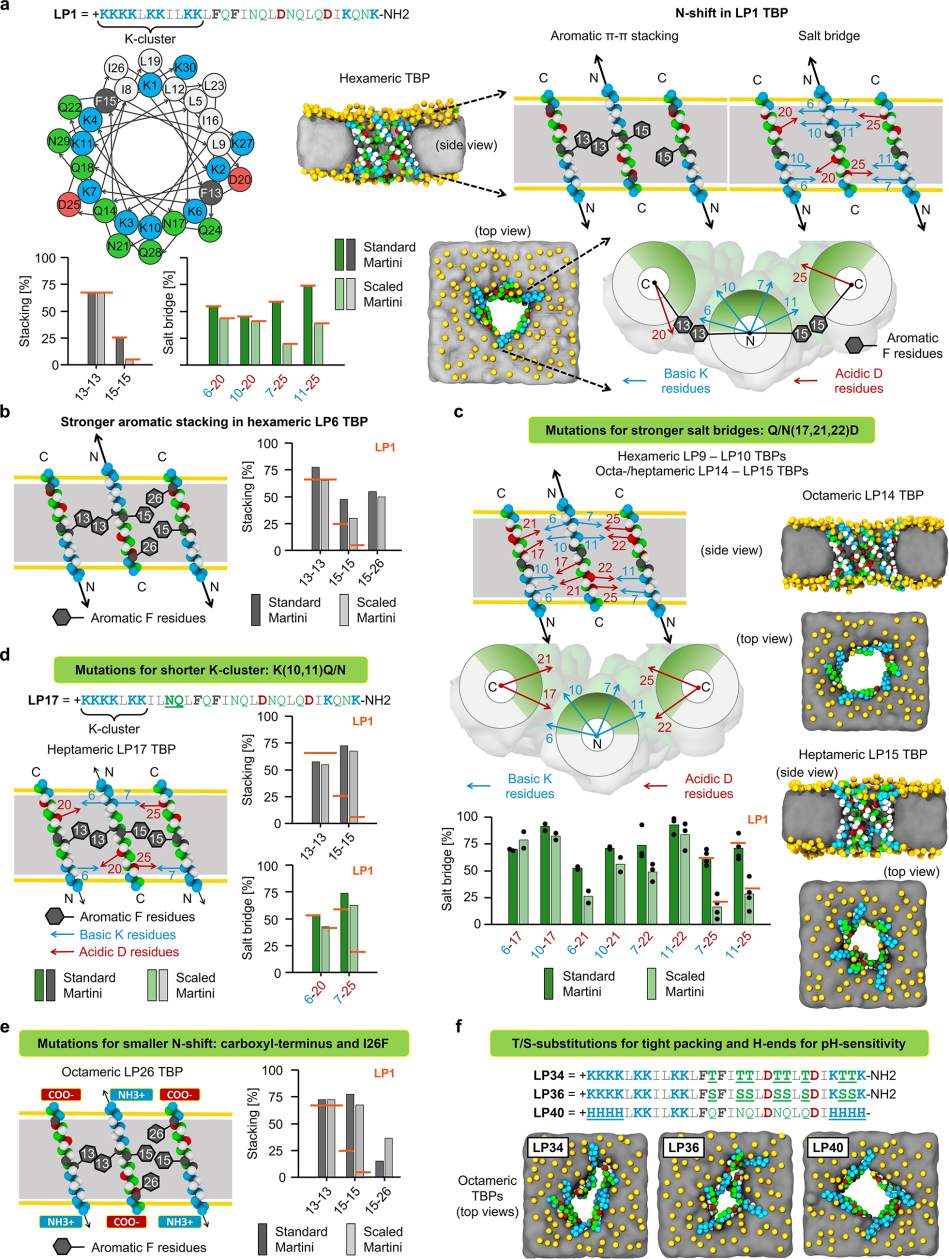
Computational design of TBP-forming peptides. Mutations favoring TBP stabilization in the “scaled” Martini simulations. (a) Helical wheel diagram of LP1, simulation snapshots of hexameric LP1 TBP, and the effect of “N-shift” motions (black arrows) on the stability of intermolecular salt bridges and aromatic stacking interactions in LP1 TBP. LP1 interaction strengths are used as a reference (orange lines). (b) Stronger aromatic stacking on the second peptide–peptide interface of hexameric LP6 TBP using I26F substitution. (c) Stronger salt bridges resulted in octameric LP14 and heptameric LP15 TBPs. (d) Shorter N-terminal K-cluster decreased the N-shift and stabilized heptameric LP17 TBP. (e) Carboxy-terminus and complementary stacking with I26F resulted in an octameric LP26 TBP. (f) T/S substitutions caused tight packing of polar faces, resulting in narrower but octameric LP34 and LP36 TBPs. Neutral H-containing peptide ends (H ends) and carboxy-terminus resulted in octameric LP40 TBP. Snapshots were taken after 51 μs simulation using the “scaled” Martini force field, showing the side and top views of TBPs in the POPC lipid membrane. Schematic illustrations are shown for three antiparallel neighboring transmembrane peptides representing two peptide–peptide interfaces of a TBP (side and top views). Stability of stacking and salt bridge interactions was calculated as the percentage of designed interaction contacts averaged over 51 μs simulation using the standard and “scaled” Martini force fields (Table 1). Color coding: peptide hydrophilic and hydrophobic residues in green and white, respectively; basic and acidic in residues blue and red, respectively; aromatic residues in gray; membrane lipid phosphates in yellow and tails as gray panel; and yellow horizontal lines in the schematic illustrations indicate the position of lipid phosphates.

Using MD simulations, we evaluated the effects of systematic mutations in the sequence of a TBP-forming 30-residue-long AMP, KDFA2i+9-NH2,^29^ hereafter referred to as LP1 (long pore-forming peptide 1). We focused on the ability of each mutation to stabilize TBP in the common zwitterionic 1-palmitoyl-2-oleoyl-*sn*-glycero-3-phosphocholine (POPC) lipid membrane (Table 1). We aimed to identify mutations that increase TBP stability via peptide–peptide interactions. Starting from a preformed octameric antiparallel peptide pore, we compared the relative stability of TBPs using both the standard and a “scaled” coarse-grained (CG) Martini (2.2) force field.^32,33^ After a 51 μs-long unbiased simulation, the pore was considered as a “stable” TBP if it consisted of at least six peptides, i.e., hexamer. In the case of pentamer and tetramer, the pore was considered “deformed”. Finally, the pore was considered “closed” if fewer than four peptides remained transmembrane. LP1 yielded octameric and hexameric TBPs using standard and scaled Martini, respectively (Figure 1a and Figure S1a). The mutations studied were classified into the following eight types.

**Table 1:**
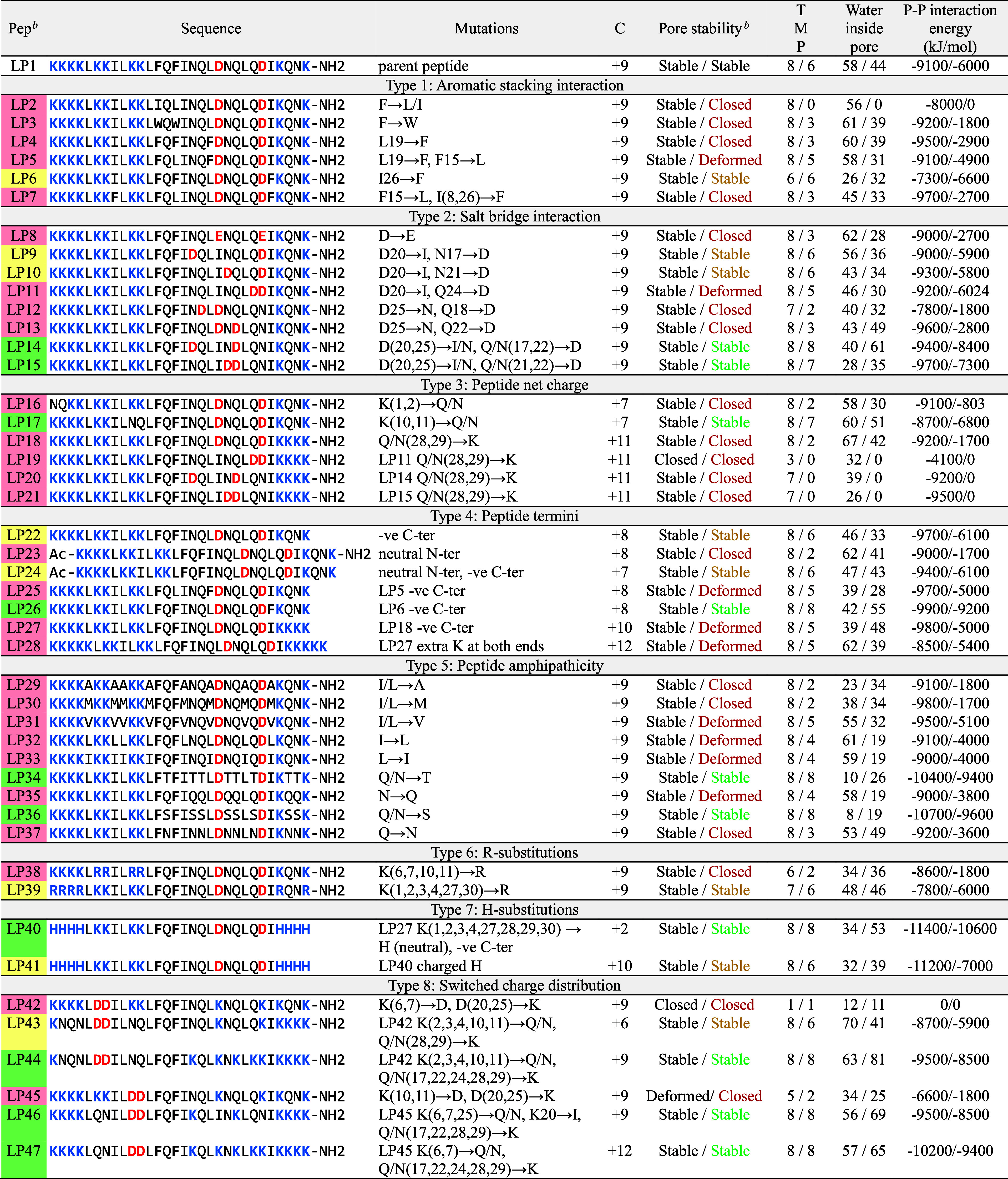
TBP-Stabilizing Activity in MD Simulations^a^

a Peptide termini were either unmodified (i.e., positively charged N-terminus and negatively charged C-terminus) or capped (acetylated N-terminus, Ac–, and amidated C-terminus, −NH_2_). C indicates peptide net charge. Pore stability in the POPC lipid membrane using the standard and “scaled” CG Martini force fields is separated by a slash (/) symbol. TMP represents the number of transmembrane peptides after 51 μs. The average number of water beads inside the pore and the transmembrane peptide–peptide interaction energy were calculated over the last 3 μs (i.e., 48–51 μs).

b Pore-stabilizing activity of the mutated peptides compared to LP1 in the “scaled” Martini simulations is colored as follows: green for increased, yellow for equivalent, and red for decreased TBP stability.

### Type 1: Aromatic π–π Stacking Interactions

LP1 was designed to stabilize TBP with 13–13 and 15–15 aromatic stacking interactions using phenylalanine (F) residues at positions 13 and 15 on the first and second peptide–peptide interfaces, respectively (Figure 1a). The importance of these F-stacking interactions was demonstrated by LP2 and LP3 peptides (with F-to-leucine (L)/isoleucine (I) and F-to-tryptophan (W) substitutions, respectively), which did not stabilize TBPs using scaled Martini (Table 1). Closer examination of LP1 TBP revealed that, due to a cluster of lysine (K) residues at the N-terminal end (i.e., eight consecutive Ks interspersed by three L/I residues), transmembrane peptides in TBP were displaced along the membrane normal to maximize hydrogen bonding and electrostatic interactions with lipid phosphates (Figure 1a). We termed this movement “N-shift”. Due to the N-shift, 15–15 stacking became unstable at the second interface. In a search for mutations that could improve F-stacking at the second interface (LP4–LP7; see Table 1), we found that I26F could form a complementary 15–26 stacking, simultaneously increasing the stability of 15–15 stacking in LP6 (Figure 1b). However, the LP6 TBP remained hexameric (Figure S1a). Other mutants with stacking interactions at different positions failed to stabilize TBP by using scaled Martini. Thus, we identified the I26F mutation as beneficial but did not increase TBP stability significantly.

### Type 2: Salt Bridge Interactions

In addition to aromatic stacking, LP1 TBP was designed with 6/10–20 and 7/11–25 K-aspartic acid (D) salt bridge interactions on the first and second peptide–peptide interfaces, respectively (Figure 1a). However, due to the N-shift, the salt bridges became unstable, especially in the scaled Martini simulation. Therefore, we sought for stronger salt bridges that could enhance the TBP stability (using LP8–LP15; see Table 1). We found that 10–17/21 salt bridges at the first interface improved TBP stability when combined with 7/11–22 salt bridges at the second interface (Figure 1c). LP14–LP15 resulted in octameric and heptameric TBPs with 10–17/21 and 7/11–22 salt bridges, whereas LP9–LP10 resulted in hexameric TBPs with 10–17/21 and 7/11–25 salt bridges (Figure S1b). Other mutants with different salt bridges did not stabilize TBP with the scaled Martini (Table 1). Therefore, we identified two combinations of mutations, Q/N-to-D either at positions 17 and 22 or 21 and 22, that increased TBP stability.

### Type 3: Peptide Net Charge

Peptide net charge is assumed to play a key role in cell selectivity, and increasing the net charge has been reported to improve antimicrobial efficacy but only up to a certain peptide-specific threshold.^9,23^ However, extra charges can interfere with the designed interactions and reduce pore stability.^29,34^ For example, in LP1, Ks are mainly clustered at the N-terminal end (eight Ks in positions 1–11), causing a N-shift and affecting the stability of TBP-stabilizing interactions (Figure 1a). It was therefore important to understand the effect of varying net charge on pore stability. First, we investigated the decrease in net charge by shortening the K-cluster from eight to six Ks at positions 3–11 and 1–7 in LP16 and LP17, respectively (Table 1). LP16, with a K-cluster deeper in the sequence, exhibited a greater N-shift, closing the pore by using scaled Martini. In contrast, LP17, which has a similar but shorter K-cluster than LP1, showed a smaller N-shift, thus stabilizing a heptameric TBP with an increased stability of the 7–25 salt bridge and 15–15 stacking at the second peptide–peptide interface (Figure 1d and Figure S1c). Therefore, shortening the K-cluster by removing Ks from the middle of the sequence increased TBP stability compared to that of LP1. Second, we investigated the increase in net charge by introducing extra Ks at the C-terminal end of LP18–LP21 (Table 1). These mutations led to intramolecular salt bridges (Figure S2a), which hindered the designed intermolecular salt bridges and closed the pore using scaled Martini. Thus, the addition of extra charges at the C-end did not increase the TBP stability.

### Type 4: Peptide Termini

The peptide termini influences several properties, including net charge, lipid-specific interactions, membrane insertion, and transmembrane peptide–peptide interactions.^29,35^ The N-terminus of LP1 was unmodified (i.e., positively charged), whereas the C-terminus was capped (i.e., amidated −NH2) to increase the overall net charge. Investigating the other combinations of capped and charged termini (using LP22–LP28; see Table 1), we observed that a negatively charged C-terminus increases the TBP stability because of the electrostatic interactions and the hydrogen bonding with the adjacent positively charged N-terminus. The combination of the I26F mutation and the negatively charged C-terminus resulted in an octameric LP26 TBP (Figures 1e and Figure S1d), whereas these individual mutations resulted in hexameric LP6 and LP22 TBPs (Table 1).

### Type 5: Peptide Amphiphilicity

The relative abundance of hydrophobic and hydrophilic residues within a peptide could influence not only antimicrobial activity and toxicity^9^ but also TBP stability.^29^ Specific residues on the polar and nonpolar faces can alter TBP stability due to the different packing of the interhelical side chains.^36−38^ We investigated the effects of the following polar and hydrophobic residues: alanine (A), methionine (M), valine (V), leucine (L), isoleucine (I), threonine (T), glutamine (Q), serine (S), and asparagine (N), using peptides LP29–LP37 (see Table 1). Residues that could disrupt the helical structure of the peptides or form disulfide bonds were not tested. Using scaled Martini, only the T and S mutants LP34 and LP36 stabilized octameric TBPs (Figure 1f), due to the tight packing of smaller polar side chains facing the pore lumen (higher peptide–peptide interaction energy; see Table 1). This tighter packing was consistent with previous reports that T and S promote helix association through hydrogen bonding networks.^39^ However, the pore cavities became too narrow for a continuous water channel in the Martini model (Figure S1e), in which each water bead represents four water molecules. Although A substitutions can induce tight packing of antiparallel helices, they must be made at specific positions and in limited numbers;^40^ otherwise, the lower hydrophobicity of A residues could reduce the desired interactions between the peptide hydrophobic patch and lipid tails, resulting in reduced TBP stability. TBP destabilization with the larger side chains suggests that these residues must be carefully positioned in the helix to avoid steric clashes and maximize the intermolecular packing in TBP.^37,38^ Optimal positioning of these residues requires an understanding of the effect of single-residue mutations in the all-atom system, which will be the subject of future research.

### Type 6: Arginine Substitutions

Unlike K-rich peptides, arginine (R)-rich peptides can aggregate in the membrane and form transient pores.^41^ To investigate TBP-stabilizing effects of Rs, we tested LP38 and LP39 with K-to-R substitutions in the middle of the sequence and at the ends, respectively (Table 1). While the R ends stabilized hexameric LP39 TBP (Figure S 1f), the midsequence R substitutions closed the LP38 pore. A higher propensity of Rs to interact with lipid phosphate and glycerol groups plausibly led to LP38 pore closure.

### Type 7: Histidine Substitutions

Histidine (H)-containing peptides could have a higher net charge at acidic pH, while keeping low net charge at neutral pH.^42,43^ Therefore, such peptides may selectively kill the negatively charged cancer cells in the acidic microenvironment while having only low toxicity for neutral normal human cells.^24,27,28^ To test the effect of H substitution on TBP stability, we simulated LP40 and LP41 with neutral and protonated H ends (i.e., K-to-H substitutions at the peptide ends; see Table 1), mimicking partially charged Hs at pH >6 and protonated Hs at pH ≤6, respectively. K residues in the middle of the sequence were not modified to preserve the K–D salt bridges that stabilize the TBP. H ends with zero charge resulted in octameric LP40 TBP (Figure 1f), whereas charged H ends resulted in hexameric LP41 TBP (Figure S1g). The lower TBP stability with charged Hs could be caused by the repulsion between neighboring H ends, the formation of intramolecular salt bridges at the C-terminal end, or both (Figure S2a).

### Type 8: Switched Charge Distribution

**Figure 2:**
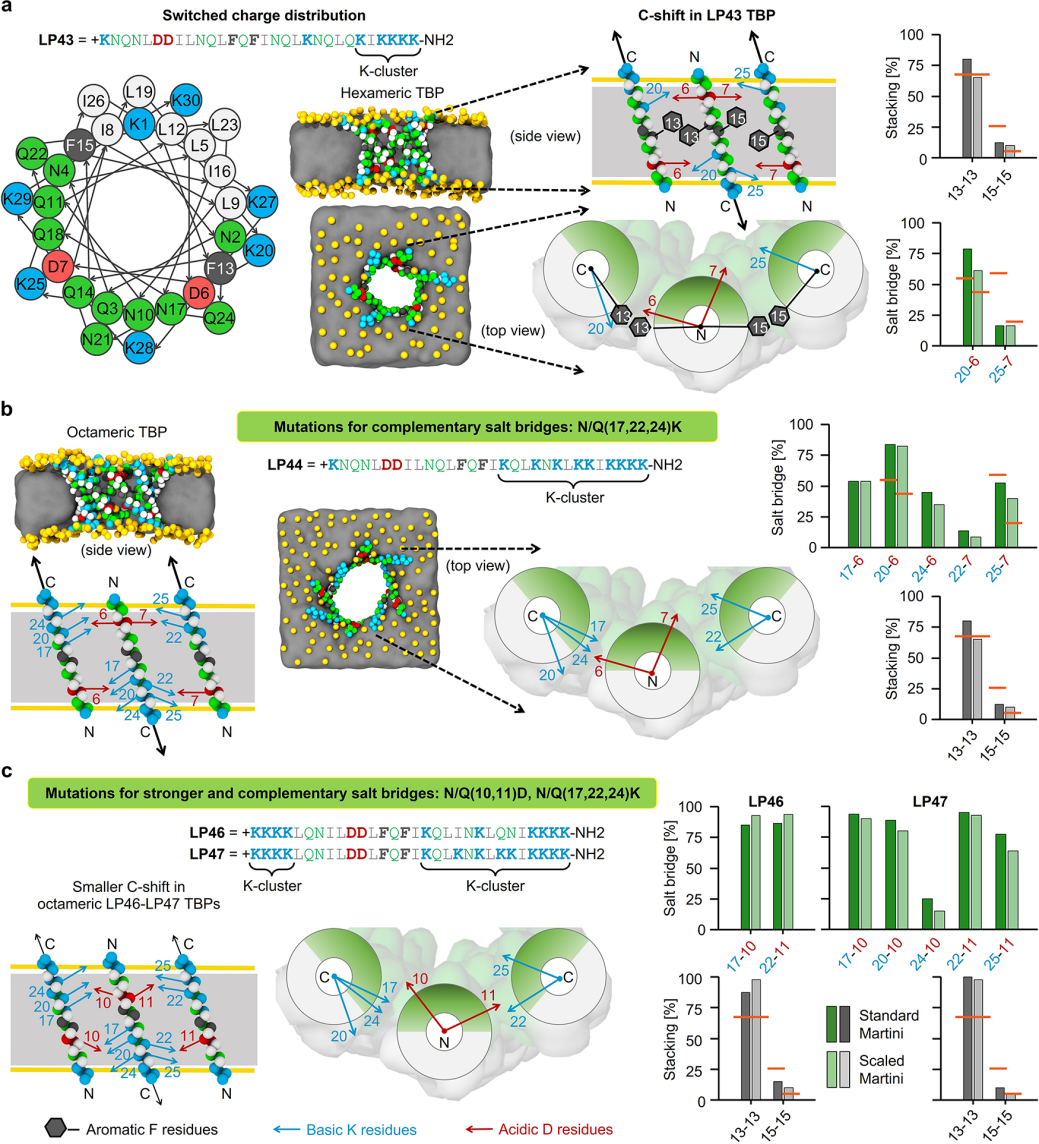
Computational design of peptides with switched charge distribution stabilizing TBPs. Mutations favoring TBP stabilization in the “scaled” Martini simulation. Helical wheel diagram (a), simulation snapshots of TBPs (a, b), schematic illustrations of “C-shift” motions (black arrows) and the intermolecular interactions, and the strength of these interactions in TBPs (a–c; orange lines indicate LP1 reference). Snapshots were captured after 51 μs simulation using “scaled” Martini force field, showing the side and top views of TBPs in POPC lipid membrane. Schematic illustrations represent three antiparallel neighboring transmembrane peptides with two peptide–peptide interfaces from a TBP (side and top views). Stability of aromatic stacking and salt bridge interactions was calculated as the percentage of designed interaction contacts averaged over 51 μs simulation using the standard and “scaled” Martini force fields (Table 1). Color coding: peptide hydrophilic and hydrophobic residues in green and white, respectively; basic and acidic residues in blue and red, respectively; aromatic residues in gray; membrane lipid phosphates in yellow and tails as gray panel; and yellow horizontal lines in the schematic illustrations indicate the position of lipid phosphates.

Peptides with different charge distributions could have different selectivities for bacteria and different kinetics of self-assembly.^44,45^ Moreover, charge distribution should have a strong effect on TBP stability (Table 1). Simply switching/swapping the positions (6, 7, 20, and 25) of the two oppositely charged K and D residues that formed salt bridges did not stabilize LP42 TBP due to the formation of intramolecular salt bridges at the N-terminal end (Figure S2b). Moving Ks from the N end to the C end removed these intramolecular interactions and LP43, similarly to LP1, stabilizing hexameric TBP (Figure 2a and Figure S1h). As expected, LP43 peptides showed a “C-shift” motion in TBP, and as a result, the 15–15 stacking and the 7–25 salt bridge at the second peptide–peptide interface were again unstable. Incorporation of additional Ks at the other salt bridge forming positions (i.e., 17, 22, and 24), resulted in octameric LP44 TBP due to the formation of complementary salt bridges (Figure 2b and Figure S1h). Similar results were obtained for LP45–LP47 with Ds at positions 10 and 11, resulting in even stronger salt bridges and smaller C shifts due to K clusters at both N and C ends (Figure 2c and Figures S1h and S2b). Thus, we identified two combinations of pore-stabilizing mutations: (1) Q/N-to-K at 17, 22, and 24, with a K-cluster only at the C end due to the presence of D6 and D7, and (2) Q/N-to-K at 17 and 22 (optionally also 24) with K clusters at both ends (with D10 and D11). Both combinations (1) and (2) resulted in increased TBP stability for the peptides with a switched charge distribution.

### Experiments

We synthesized 22 mutant peptides and tested them experimentally for antimicrobial activity against ESKAPEE pathogens and for toxicity to normal human cells. The mechanism of antimicrobial activity was then investigated using liposomal leakage, bacterial membrane disruption, and AFM experiments. Selected peptides were also tested for their pH-dependent anticancer activity.

The peptides were selected from all eight types, regardless of their TBP-stabilizing activity in “scaled” Martini simulations (Table 2). LP2, LP3, and LP8 (types 1 and 2) were tested without aromatic stacking, with W-stacking, and with K–E salt bridges, respectively. LP18 and LP20 (type 3) were tested to find the effect of increasing peptide net charge from +9 e to +11 e by making K-substitutions at the C-terminal end. LP22, LP23, and LP26 (type 4) were tested by combining a cationic N-terminus with an anionic C-terminus, an acetylated N-terminus with an amidated C-terminus, and an anionic C-terminus with the I26F mutation. In addition, we tested LP28 with additional Ks at both ends, together with an anionic C-terminus (32 residues long peptide with +12 e net charge). All hydrophobic and hydrophilic variants LP29–LP40 (types 5–7) were tested. Finally, we tested LP43 (type 8), a peptide with switched charge distribution. The peptides were synthesized with >95% purity in high-performance liquid chromatography (HPLC, see the Supporting Information) and were soluble in PBS.

**Table 2:**
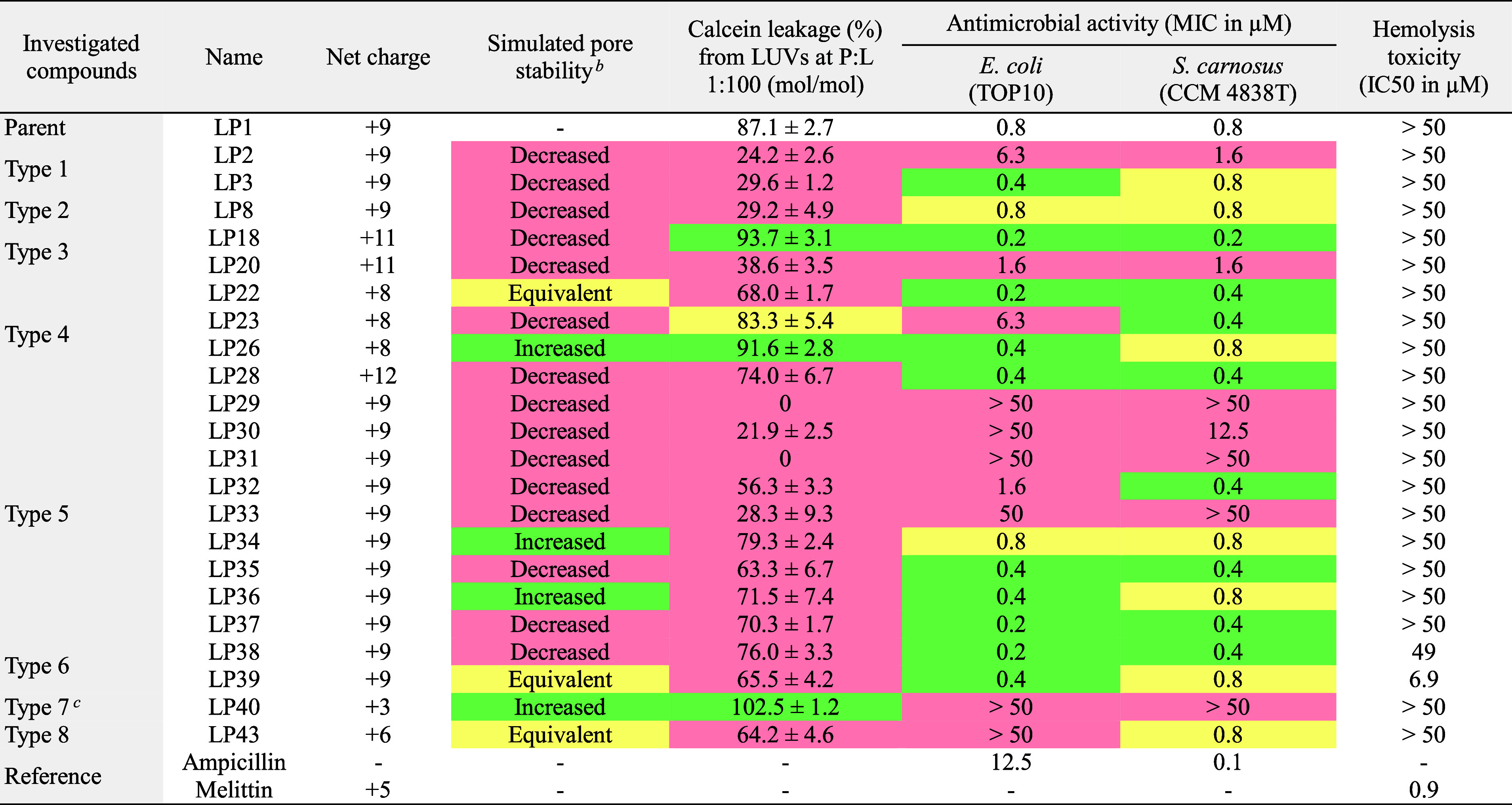
*In Vitro* Activity and Toxicity^a^

a Activities of the mutated peptides compared to LP1 are colored as follows: green for increased, yellow for equivalent, and red for decreased activity.

b Pore stability in POPC lipid membrane compared to LP1 in the “scaled” Martini simulations (see Table 1).

c At physiological pH 7.4, the H residues in LP40 are only partially charged (i.e., carry less than +1 e charge). However, in the presence of negatively charged lipids, the charge of H residues is likely to be higher due to protonation.

### *In Vitro* Antimicrobial Activity

To test antimicrobial activity, all peptides were initially screened, in a broth microdilution assay, against Gram-negative *Escherichia coli* (TOP10) and Gram-positive *Staphylococcus carnosus* (CCM 4838T) Biosafety Level 1 (BSL-1) bacteria (Table 2). Six mutants (LP18, LP22, LP28, LP35, LP37, and LP38) showed higher activity than LP1 against both strains, having MIC values, i.e., the lowest concentration that inhibited visible growth of bacteria, as low as 0.2 μM, whereas the MIC of LP1 reached 0.8 μM. Four other mutants (LP3, LP26, LP36, and LP39) had higher activity than LP1 against *E. coli*, and two mutants (LP23 and LP32) had higher activity than LP1 against *S. carnosus*. Six mutants (LP29, LP30, LP31, LP33, LP40, and LP43) were not active against one or both bacteria, even at the highest concentration tested (50 μM).

**Figure 3:**
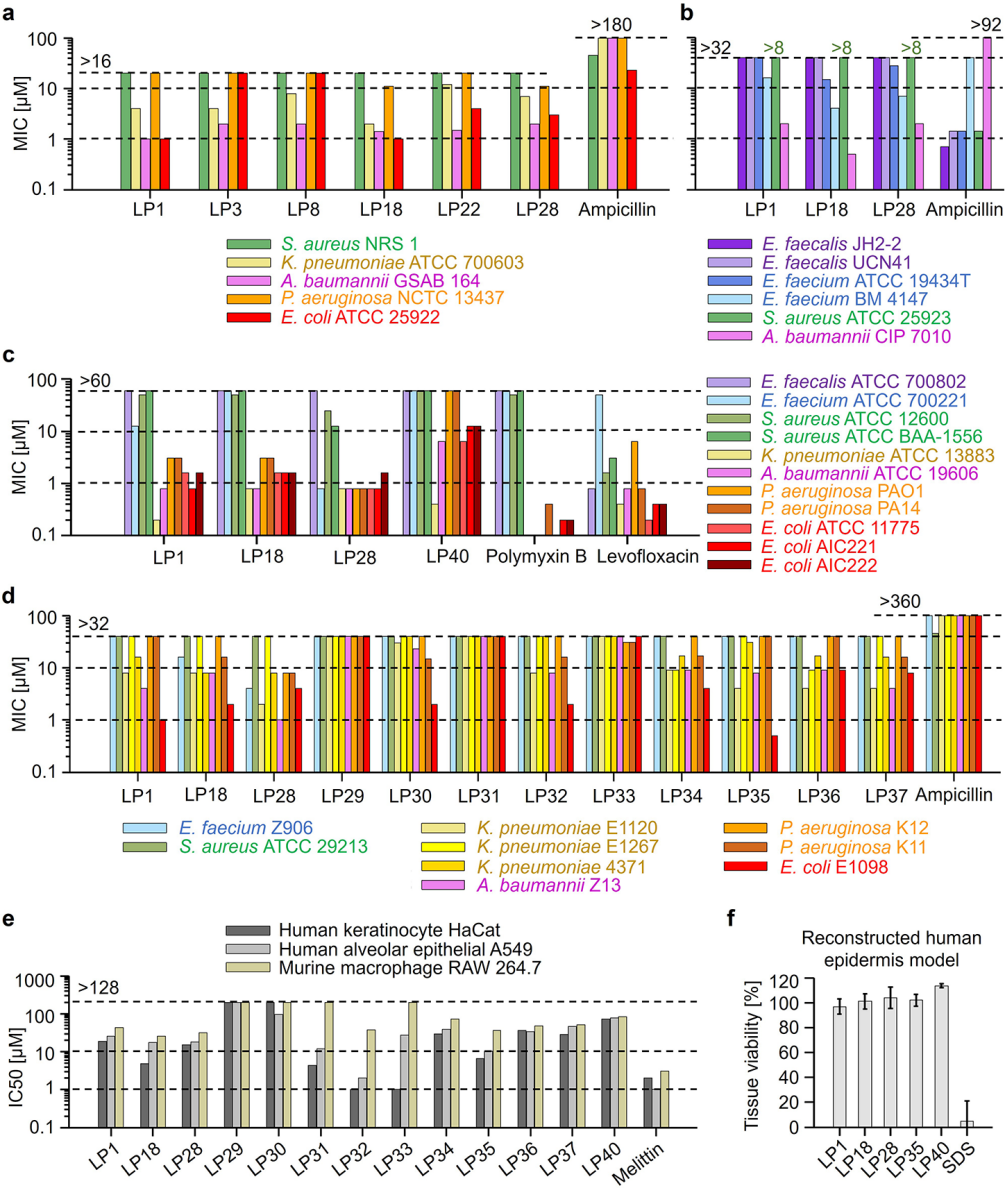
*In vitro* antimicrobial activity and toxicity. (a–d) Antimicrobial activity is reported as the MIC values against Gram-positive (colored violet–blue–green) and Gram-negative (colored yellow–pink–orange–red) ESKAPEE pathogens. (e) Cytotoxicity against human keratinocyte cells HaCaT, human alveolar epithelial cells A549 (derived from adenocarcinoma), and murine macrophage cells RAW 264.7 is reported as the IC50 values after 24 h of peptide treatment. (f) Toxicity against the reconstructed human epidermis model is reported as the average tissue viability after 1 h treatment with peptides at 50 μM concentration and 42 h of post-treatment incubation. Ampicillin, polymyxin B, levofloxacin, melittin, and sodium dodecyl sulfate (SDS) were used as controls. Peptide mutations tested are as follows—LP3: F→W for aromatic W-stacking; LP8: D→E for K–E salt bridges; LP18: +2 e increase in net charge; LP22: negatively charged C-terminus; LP28: +3 e increase in net charge; LP29–LP37: A-, M-, V-, L-, I-, T-, Q-, S-, N-variants; LP40: H-variant with +2 e net charge; sequences are given in Table 1.

We tested the antimicrobial activity of selected peptides against ESKAPEE pathogens (Figure 3a–d and Figure S3a). In the first set of experiments (Figure 3a), the peptides tested were selected based on the results of the initial screening (Table 2) and the bacterial strains tested were the commercially available ones. Peptide net charge appeared to have an effect on activity, which was then investigated first against the reference strains in the second set of experiments (Figure 3b) and then against strains susceptible to the conventional antibiotics in the third set of experiments (Figure 3c). Finally, the most promising cationic peptides and all hydrophobic and hydrophilic variants were tested against ampicillin-resistant strains to see the effect of both net charge and specific amino acids, the fourth set of experiments (Figure 3d).

First, LP1 and five mutants (LP3, LP8, LP18, LP22, and LP28) were tested against five commercially available ESKAPEE strains (Figure 3a). Promising activity was obtained against Gram-negative *Acinetobacter baumannii* (GSAB 164), which is resistant to colistin and meropenem, followed by a quality control strain of *E. coli* (ATCC 25922). Micromolar activity, i.e., MIC of 1 μM, was obtained with both LP1 and LP18.

Overall, increasing the net charge from +9 to +11 e increased the activity of LP18. However, a further increase in the net charge to +12 e did not increase the activity of LP28. The effect of net charge was further investigated against six reference strains of ESKAPEE pathogens (Figure 3b). Promising activity was obtained against *A. baumannii* (CIP 7010 or ATCC 15151). LP18, with submicromolar MIC of 0.5 μM, was four times more active than LP1 and LP28. We next found that the effect of net charge is complicated and rather strain specific, as observed against 11 ESKAPEE strains mostly susceptible to the conventional antibiotics polymyxin B and levofloxacin (Figure 3c). Submicromolar activity (i.e., MIC <1 μM, see Figure S3a) was observed against vancomycin-resistant *E. faecium* (ATCC 700221; LP28 MIC = 0.8 μM), *K. pneumoniae* (ATCC 13883; LP1 MIC = 0.2 μM; LP18 and LP28 MIC = 0.8 μM), *A. baumannii* (ATCC 19606; all MIC = 0.8 μM), *P. aeruginosa* (PAO1 and PA14; LP28 MIC = 0.8 μM), and *E. coli* (ATCC 11775; LP28 MIC = 0.8 μM; and AIC221; LP1 and LP28 MIC = 0.8 μM). We also tested the H-variant LP40 to see the effect of a reduced net charge (+3 e at pH 7.4). As expected, the activity of LP40 was consistently lower than the other three peptides, with a surprising submicromolar activity against *K. pneumoniae* (MIC = 0.4 μM) (Figure 3c).

The cationic peptides, LP1, LP18, and LP28, consistently maintained their activity across various strains including colistin resistant ones, confirming different mechanism of action at membrane (Figure S3a). Apart from the net charge, a specific amino acid could have a strong effect on a specific bacterial strain. Therefore, in the final set of experiments, three cationic peptides and all hydrophobic and hydrophilic variants (LP29–LP37) were tested against nine ampicillin-resistant ESKAPEE strains (Figure 3d). The activity against the multidrug-resistant strains of *E. coli* (E1098) and *A. baumannii* (Z13) was particularly promising. Interestingly, against *E. coli*, the Q-variant LP35 had submicromolar (MIC = 0.5 μM) activity, two times more active than LP1 (MIC = 1 μM), and against *A. baumannii*, LP28 had micromolar activity (MIC = 1 μM), four to eight times more active than LP1 and LP18.

Overall, our peptides were less effective against Gram-positive bacteria, particularly *S. aureus*. At the highest tested concentration of 30 μM, the peptides inhibited only 45% growth of the community-associated methicillin-resistant *S. aureus* (CA-MRSA) strain USA300 LAC derivative JE (Figure S3b). This suggests that our peptides may have been degraded by the extracellular proteases of *S. aureus*.^46,47^ To identify the proteases, we tested 12 peptides (LP1, cationic LP18, and polar–apolar variants LP28–LP37) against single transposon insertion mutants derived from JE2. The insertions had been made in genes encoding the proteases aureolysin (NE 163; aur::Tn::Erm), V8 protease (NE1506; sspA::Tn::Erm, staphopain A (NE1740; scpA::Tn::Erm), and staphopain B (NE934; sspB::Tn::Erm).^48,49^ We also tested the protease null mutant AH 1919 (LAC*Δaur ΔsspAB ΔscpA spl::erm),^50^ which lacks the above proteases and the serine proteases SplABCDEF. Peptides did not inhibit the growth of transposon insertion mutants by more than 45%, suggesting that some yet unidentified protease or other unknown factor hinders the activity of these peptides against CA-MRSA.

### *In Vitro* Toxicity on Representative Mammalian Cell Lines

To investigate the toxicity for normal human cells, all peptides were first screened against human red blood cells (HRBCs) using a hemolysis assay (Table 2 and Figure S4). All mutants were found to be nonhemolytic up to the highest tested concentration of 50 μM, with the exception of two R-variants, LP38 and LP39. The toxicity of R-rich peptides is consistent with our previous report, in which the peptide RDFA2i+9-NH2, in which all Ks in LP1 were replaced by Rs, showed hemolysis similar to that of reference hemolytic peptide, melittin, with a half-maximal inhibitory concentration, i.e., IC50 value, of 0.9 μM.^29^ LP39, which has R substitutions only at the ends, was less toxic (IC50 = 6.9 μM) than melittin, and LP38, which has R substitutions only in the middle of the sequence, was even less toxic (IC50 = 49 μM) than LP39. The higher toxicity of R ends (LP39) compared to R-middle (LP38) was most likely due to the stronger interactions of peptide ends of TBPs with phospholipids present in human membranes.^51^

To further assess toxicity, we chose to test LP1, two promising cationic mutants (LP18 and LP28), the hydrophobic and hydrophilic variants (LP29–LP37), and the H-variant (LP40) against human immortalized keratinocytes HaCaT, human alveolar epithelial type II cells A549 (although derived from adenocarcinoma, but commonly used for toxicity studies^52^), and murine macrophages RAW 264.7 using an MTT-based assay (Figure 3e). After 24 h of incubation, the IC50 values of the four highly active AMPs, LP1, LP18, LP28, and LP35, were at least one order of magnitude higher than the bacterial MIC of 0.5 μM. Among these peptides, LP18 and the Q-variant LP35 showed the highest toxicity, followed by LP28 and LP1. Against HaCaT, LP18, LP35, LP28, and LP1 had the IC50 values of 5, 7, 15, and 19 μM, while the same peptides had the IC50 values of 18, 10, 18, and 26 μM against A549 and the IC50 values of 25, 36, 31, and 44 μM against RAW 264.7 cells. The hydrophobic V/L/I-variants (LP31–LP33) showed significant toxicity, of which the L-variant LP32 had the highest cytotoxicity, followed by the I-variant LP33 and V-variant LP31. Against HaCaT, LP32, LP33, and LP31 had the IC50 values of <2, <2, and 4 μM, while the same peptides had the IC50 values of 2, 27, and 12 μM against A549 and the IC50 values of 38, >128, and >128 μM against RAW 264.7 cells. The cytotoxicity of LP32 was comparable to that of the reference toxic peptide melittin (IC50 values were 2, <2, and 3 μM against HaCaT, A549, and RAW 264.7, respectively). The H-variant LP40 had IC50 values consistently above 70 μM.

Finally, four potent antibacterial candidates (LP1, LP18, LP28, and LP35) and the H-variant (LP40) were tested for skin toxicity against a reconstructed human skin model consisting of a functional epidermis and stratum corneum. The OECD TG 439 guidelines for *in vitro* skin irritation testing were followed. The peptides were found to be nonirritating to human skin at the tested concentration of 50 μM (100 times higher than the bacterial MIC of 0.5 μM), with 100% tissue viability after 1 h exposure and 42 h of incubation (Figure 3f).

### Stability in Human Serum

One of the major limitations of AMPs is their low resistance to proteolytic degradation.^53^ For systemic administration, the peptides must be able to withstand proteolytic cleavage in the blood. To assess this ability, we incubated our three potent cationic AMPs (LP1, LP18, LP28) and the H-variant (LP40) in 25% human serum.^54,55^ Peptides remained stable, i.e., their half-lives (i.e., 50% stability) were consistently above 6 h (Figure S5). The susceptibility to proteolytic degradation increased with the number of basic residues in the order (from lowest to highest) of LP1, LP18, LP40, and LP28.

### *In Vivo* Anti-Infective and Wound-Healing Properties

**Figure 4:**
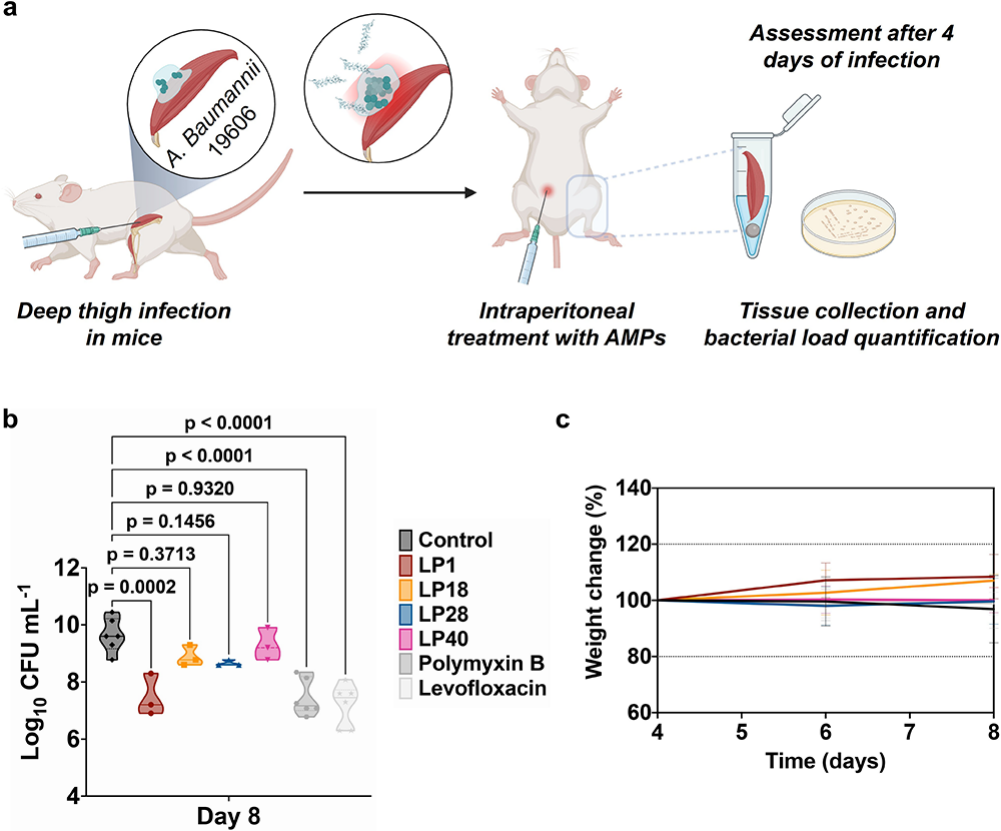
Anti-infective activity in the deep thigh infection mouse model. (a) Schematic of the deep thigh infection model in which bacteria are injected intramuscularly at day 4 and peptides are administered intraperitoneally also at day 4 to assess their anti-infective activity. Mice were euthanized 4 days postinfection (day 8). Each group consisted of six mice (*n* = 6), and the bacterial loads used to infect the mice derived from three different inocula. (b) Intraperitoneal treatment with the peptides at 10-fold MIC (i.e., LP1: 0.16 mg/kg, LP18: 0.17 mg/kg, LP28: 0.18 mg/kg, LP40: 1.33 mg/kg) reduced the bacterial load of *A. baumannii* (ATCC 19606) compared to the untreated control group. Polymyxin B (0.006 mg/kg) and levofloxacin (0.014 mg/kg) were used as the reference antibiotics. Statistical significance was done using one-way ANOVA followed by Dunnett’s test. Violin plots represent the median, upper quartile, and lower quartile. (c) Mouse weight was monitored to exclude possible toxic effects of the peptides. Peptide sequences are shown in Table 1.

Four peptides (LP1, LP18, LP28, and LP40), which had been thoroughly evaluated for *in vitro* stability, activity, and cytotoxicity, were next tested for *in vivo* anti-infective activity in a murine deep thigh infection model^56^ (Figure 4a). Neutropenic mice were infected intramuscularly (in the right thigh) with 10^6^ cells per mL of Gram-negative *A. baumannii* (ATCC 19606). A single dose of peptide at 10 times the MIC (LP1: 0.16 mg/kg; LP18: 0.17 mg/kg; LP28: 0.18 mg/kg; LP40: 1.33 mg/kg) was administered intraperitoneally. Four days after treatment, LP1 reduced the bacterial load by two to three orders of magnitude, comparable to the reference antibiotics polymyxin B and levofloxacin (Figure 4b). However, the other peptides did not significantly reduce the bacterial load. There was no significant change in mouse weight, indicating a lack of peptide toxicity (Figure 4c).

To investigate wound-healing applications, two peptides (LP1 and LP18) were tested in a murine wound infection model.^57^ Immunosuppressed mice were infected with 5 × 10^4^ cells per mL of *A. baumannii* (NCTC 13301) at the excised dorsal wound. The peptides at 1000 times the MICs (∼10 mg/kg) were applied directly to the wound by puncturing the dressing with a syringe once a day for 7 days. After removal of the dressing, necrosis of the epidermal tissue at the wound edges and wound expansion were observed. These conditions were reduced in the LP1-treated group, followed by the LP18-treated, PBS-treated, and untreated groups (Figure S6a,b). Contractile wound healing appeared to be higher in the LP1-treated group compared to the other groups. After 20 days, LP1-treated wounds were either completely closed or had less than 10 mm^2^ of overlying serocellular crust, whereas the other wounds had larger and more inflamed serocellular crusts. The bacterial load in the wounds, although gradually decreasing, was surprisingly similar in the treated and untreated groups (Figure S6c). At day 3 postinfection, the untreated group had a 30% decrease in survival compared to 100% survival in the treated groups (Figure S6d). Notably, after 45 days, there was a dramatic loss of body weight and survival in both the treated and untreated groups (Figure S6d,e). This suggests proteolytic degradation and promiscuous peptide binding/aggregation with host factors, also indicated by the bacterial load.

### Dye Leakage Activity Using Lipid Vesicles

**Figure 5:**
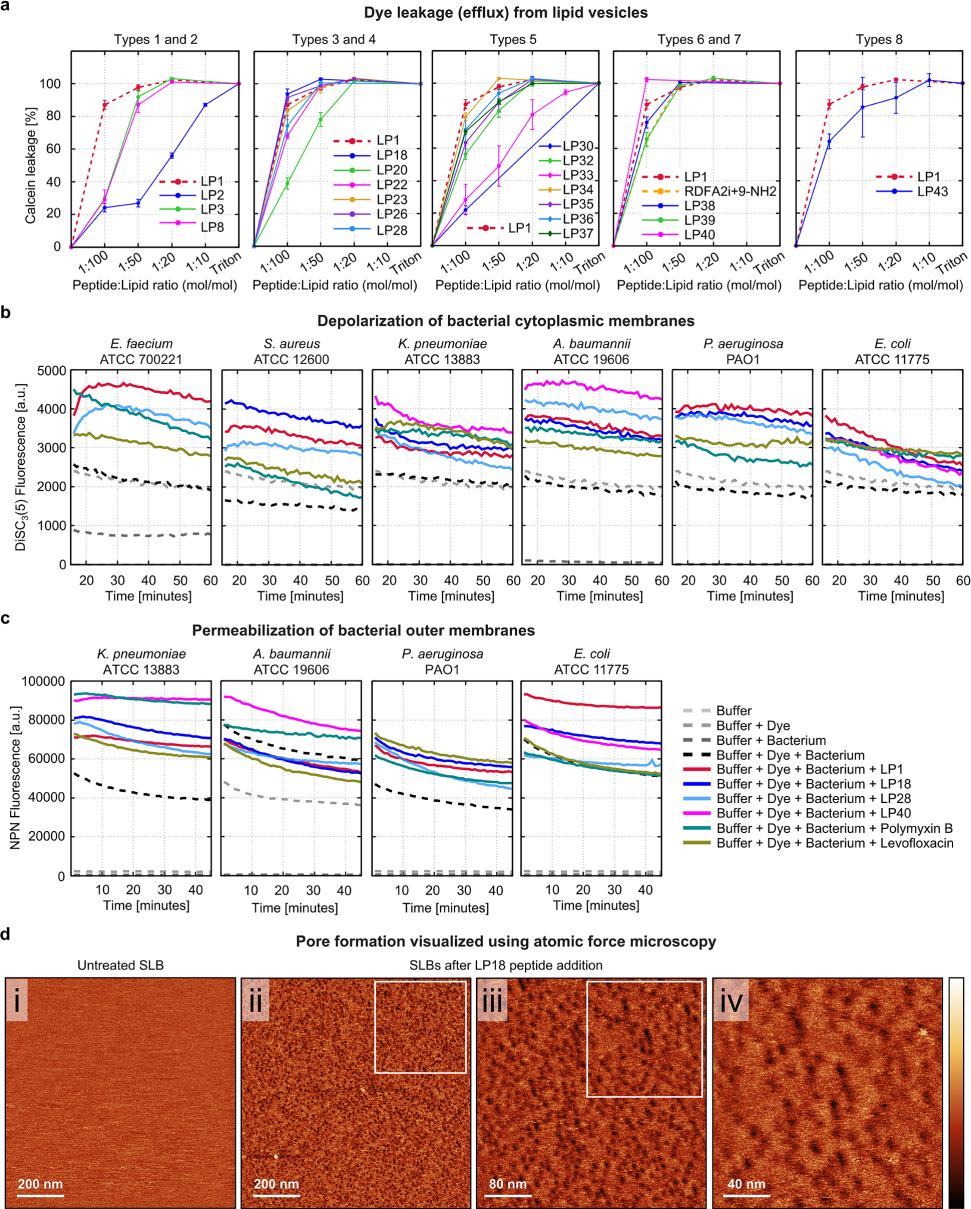
Pore-forming mechanism of action of designed AMPs. (a) Peptide-induced leakage of the fluorescent dye calcein from the LUVs composed of POPC:POPG (1:1 mol/mol) lipids. Surfactant triton was used as the control, causing 100% leakage in the end. (b) Peptide-induced depolarization of the bacterial cytoplasmic membranes is detected as an increase in the fluorescence intensity of the membrane potential-sensitive dye DiSC_3_-(5). (c) Peptide-induced permeabilization of Gram-negative bacterial outer membranes is indicated as an increase in fluorescence intensity of the lipophilic dye NPN. Peptides were tested at their MIC values against the respective bacteria. Polymyxin B and levofloxacin were used as reference antibiotics. (d) AFM topographical images of (i) the initial untreated, homogeneous, defect-free, and vesicle-free SLB (1 × 1 μm^2^) and (ii–iv) LP18 peptide-treated SLBs captured successively at increasing resolution (1 × 1 μm^2^ > 400 × 400 nm^2^ > 200 × 200 nm^2^). Color scale (height): 2 nm. SLBs were composed of POPC:POPG lipids (1:1 mol/mol). The studied P:L molecular ratio is 1:500, which corresponds to a final peptide concentration of 1 μM. Peptide sequences are given in Table 1.

To verify the pore-forming mechanism of action of the designed AMPs, we performed a calcein leakage assay. Twenty out of the 22 mutants showed concentration-dependent leakage (efflux) of the fluorescent dye calcein from large unilamellar vesicles (LUVs) composed of 1:1 mol/mol POPC:POPG (1-palmitoyl-2-oleoyl-*sn*-glycero-3-phospho-1′-rac-glycerol) lipids (Figure 5a). These lipid vesicles were used as simple mimics of bacterial membranes.^58^ At the lowest tested peptide-to-lipid (P:L) ratio of 1:100 (mol/mol), three mutants (LP18, LP26, and LP40) caused >90% leakage, which was higher than the leakage caused by LP1 (87%). Interestingly, with a net charge of only +3 e, the H-variant LP40 caused 100% leakage at the physiological pH of 7.4. At the same concentration, LP23 caused leakage equivalent to that caused by LP1. Other mutants had lower leakage activity. At a higher P:L ratio of 1:50, all the abovementioned mutants caused 100% leakage, equivalent to that caused by LP1. Notably, the A-variant LP29 did not cause any leakage, and the V-variant LP31 caused only minimal leakage (≤20%) even at a higher P:L ratio of 1:10 (Figure S7).

### Disruption of Bacterial Membranes

While both Gram-negative and Gram-positive bacterial cells are surrounded by a cytoplasmic membrane, Gram-negative bacteria also have an additional outer membrane.^59^ Having studied the effects of peptides on the bacterial membrane mimicking lipid vesicles, we next investigated their effects on the real bacterial membranes using the following two assays.

First, we investigated the ability of the peptides to depolarize the cytoplasmic membranes of ESKAPEE pathogens. We chose to test the Gram-negative strains *K. pneumoniae* (ATCC 13883), *A. baumannii* (ATCC 19606), *P. aeruginosa* (PAO1), and *E. coli* (ATCC 11775), which were susceptible to our designed AMPs (submicromolar MICs), and two Gram-positive strains *S. aureus* (ATCC 12600) and *E. faecium* (ATCC 700221), which showed some degree of resistance with only one exception. Against these strains, our AMPs had activity comparable to that of the conventional antibiotics polymyxin B and levofloxacin (see Figure 3c), which can be used for comparisons of membrane-disrupting activity. Bacteria were incubated with 3,3′-dipropylthiadicarbocyanine iodide [DiSC_3_-(5)] dye, and the dye fluorescence was measured after peptide treatment. DiSC_3_-(5) is sensitive to transmembrane potential changes. It accumulates in the cytoplasmic membrane with quenched fluorescence, and when the membrane potential becomes unbalanced, the dye molecules translocate to the outside environment, resulting in increased fluorescence.^56^ The three potent AMPs (LP1, LP18, and LP28) and the H-variant (LP40) depolarized the cytoplasmic membrane of both Gram-negative and Gram-positive bacteria at their MICs. The extent of membrane depolarization was either greater than or comparable to that of the reference antibiotics polymyxin B and levofloxacin (Figure 5b).

Second, we investigated the ability of peptides to permeabilize the outer membrane of Gram-negative ESKAPEE pathogens *K. pneumoniae* (ATCC 13883), *A. baumannii* (ATCC 19606), *P. aeruginosa* (PAO1), and *E. coli* (ATCC 11775) using the 1-(*N*-phenylamino) naphthalene (NPN) uptake assay. NPN is a lipophilic dye that does not permeate the outer membrane unless the membrane integrity is compromised, resulting in increased fluorescence.^56^ The tested peptides permeabilized the outer membrane of the Gram-negative bacteria, except *A. baumannii*, at their MICs, either more than or as much as the reference antibiotics (Figure 5c). Interestingly, our peptides are the most active against *A. baumannii*, whose outer membrane contains lipooligosaccharides.^60^

### AFM Images of Pores Formed on Supported Lipid Membranes

To directly visualize the membrane pores formed by the designed AMPs, we performed real-time AFM imaging of supported lipid bilayers (SLBs) treated with highly active mutant AMP LP18 (Figure 5d). The SLBs were composed of POPC:POPG (1:1 mol/mol) lipids, the same composition used in the dye leakage assay. At a P:L molecular ratio of 1:500 (equivalent to 1 μM peptide concentration), LP18 formed a significantly higher number of membrane defects or pores than LP1.^29^ The pores were stable for an investigation period of 2 h and were visibly more numerous than those produced by LP1.^29^

### Effect of pH and Media on *In Vitro* Antimicrobial and Anticancer Activity

**Figure 6:**
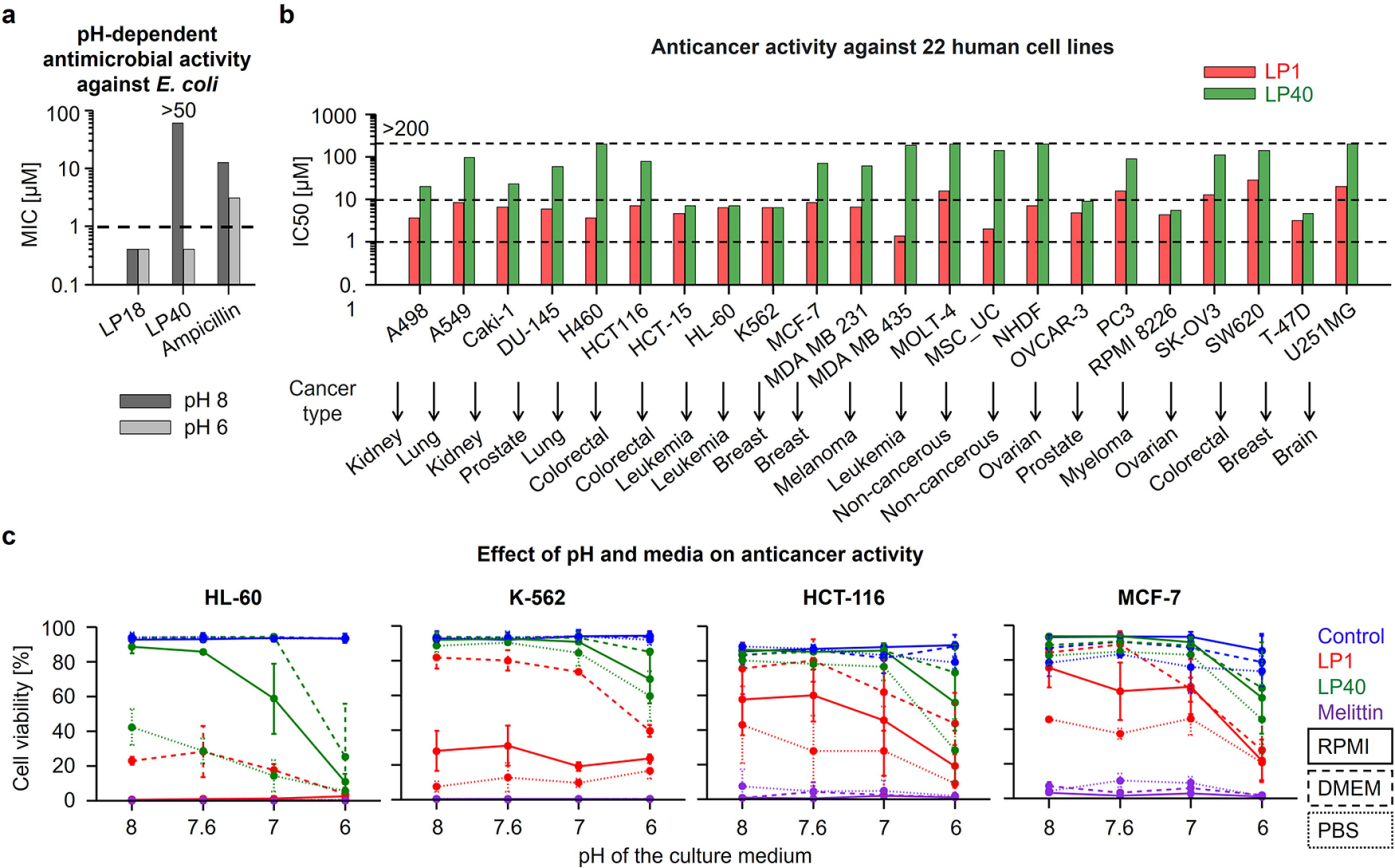
The pH-dependent *in vitro* antimicrobial and anticancer activity. (a) Antimicrobial activity against *E. coli* (TOP10) cultured at two different pH is reported as the MIC values. (b) Activity against a panel of 22 different cancerous and noncancerous human cell lines is reported as IC50 values after 72 h of peptide treatment at the physiological pH. (c) Anticancer activity against four different cancerous human cell lines cultured in three different media, RPMI, DMEM, and PBS, which are adjusted to three different pH values 8, 7, and 6, and additionally one media without pH adjustment (∼7.6) is reported as the cell viability after 1 h treatment with peptides at 10 μM concentration. Peptide melittin, antibiotic ampicillin, and PBS buffer were used as controls. The graphs represent two independent repetitions. Peptide sequences are listed in Table 1.

TBP-forming peptides can be tuned for the pH sensitivity. In particular, the H-variant LP40 is expected to have a higher net charge at lower pH (because the p*K*_a_ of H residue is ∼6^42,43^). Such an increased net charge at low pH could strengthen the peptide affinity for the negatively charged bacterial and cancer cell membranes. LP40 had the highest leakage (pore-forming) activity, yet it showed no antimicrobial activity and cytotoxicity up to the highest tested concentration of 50 μM (see Table 2). This difference in activity could be due to the low membrane affinity at neutral pH 8 of the bacterial growth media. To investigate the pH effect, we first tested the antimicrobial activity of LP40 against *E. coli* (TOP10) at both pH 8 and 6, with a K-variant AMP, LP18, as a reference. As expected, LP40 showed promising antibacterial activity with an MIC of 0.4 μM at pH 6, whereas the activity of LP18 remained independent of pH (Figure 6a).

First, we tested LP40 and LP1 for anticancer activity at the physiological pH. The peptides were screened against a panel of 20 different human cancer and 2 noncancerous cell lines using a CellTiter-Glo luminescent cell viability assay (Figure 6b). After 72 h of incubation, the K-variant LP1 had the IC50 values in the range of 1–20 μM, while the IC50 values of the H-variant LP40 were mostly above 20 μM, including noncancerous cell lines with IC50 above 100 μM. For few exceptional cell lines, the activity of LP40 was below 10 μM.

Next, we confirmed that the anticancer activity of our peptides was dose dependent. In another assay using the WST-1 reagent, peptides inhibited the growth of three cancer cell lines: acute myeloid leukemia (MOLM-13), chronic lymphocytic leukemia (MEC-1), and diffuse large B-cell lymphoma (SU-DHL-4) at the physiological pH (Figure S8). LP1 had the IC50 values in the range of 0–10 μM, while the IC50 values of LP40 were in the slightly higher range of 1–20 μM, after 24 h of incubation. The reference peptide melittin showed higher activity with the IC50 values in the range of 0–5 μM. The anticancer activity decreased moderately after 72 h of incubation, presumably due to proteolytic degradation of the peptides.

Interestingly, we found that the medium in which the cancer cell lines were cultured had an effect on the peptide activity, as shown in the panel of 22 different cancer cell lines (Table S1). In general, cells cultured in the RPMI medium were found to be more sensitive than those cultured in DMEM (Figure S9a). We further verified the improved anticancer activity in RPMI compared to DMEM by testing the peptides against three cell lines derived from acute myeloid leukemia (HL-60), chronic myeloid leukemia (K-562), and breast adenocarcinoma (MCF-7) (Figure S9b). The improved activity could be explained by the lower pH of RPMI compared to that of DMEM medium (Figure S9c).

To investigate the effect of pH and media on anticancer activity, we further tested the peptides against four cell lines, HL-60, K-562, and MCF-7, and the colon cancer cells HCT-116, cultured in three different media, namely, RPMI, DMEM, and PBS, each adjusted to three different pH values 8, 7, and 6, and additionally one case without pH adjustment (∼7.6) (Figure 6c). To avoid pH changes due to the natural buffering of the CO_2_-bicarbonate system used in cell culture, the peptides were tested at a concentration of 10 μM for only 1 h, as the cell lysis effect was already apparent within 30 min of exposure (Figure S9d). Similar to the pH-dependent antibacterial activity, both LP1 and LP40 showed a pH-dependent activity against cancer cells, with a higher dependence obtained with LP40 (ANOVA, *p* = 0.002) (Figure S10a,b). Further analysis showed that the culture medium had a strong effect on the pH-dependent activity of both LP40 (ANOVA, *p* = 0.049) and LP1 (ANOVA, *p* < 0.001) peptides, and it varied with individual cell lines. As expected, the reference peptide melittin did not allow cell growth, regardless of pH and medium.

Furthermore, immunoblotting detection of cleaved PARP and caspase-3 markers confirmed the induction of apoptosis in MCF-7 cells by the peptides (Figure S10c). The level of cleaved proteins (corresponding to the increase in apoptosis) was higher at pH 6 and, as expected, particularly for LP40.

Finally, we tested the effect of LP1 and LP40 on healthy primary cells in whole blood from a healthy donor (Figure S11). There was no apparent deleterious effect of the peptides against normal B and T lymphocytes, granulocytes, and monocytes up to the highest tested concentration of 50 μM, suggesting a wide therapeutic window.

## Discussion

### MD Simulations Revealed Mutations that Increased TBP Stability

We hypothesized that the antimicrobial activity of pore-forming peptides could be improved by optimizing intermolecular peptide–peptide interactions to enhance the pore stability. Therefore, using LP1, the most potent TBP-forming AMP from the previous study,^29^ as a template, we systematically investigated the effects of different mutations on TBP stability using MD simulations. For simulations, we selected the CG Martini 2.2 force field because of its widespread use and computational efficiency,^61^ which allowed us to study a large number of big systems over long time scales. Moreover, we took advantage of two versions of this force field. Standard Martini may overestimate peptide–peptide interactions,^29,33^ while the “scaled” Martini with downscaled peptide–peptide interactions may underestimate these interactions.^33^ With studied or mutated peptides, TBPs remained mostly stable using standard Martini but deformed or closed using scaled Martini (Figure S12a). Because of this clear distinction in pore stability, we focused on scaled Martini to identify the peptides with mutations that increased TBP stability. Of the 46 mutated peptides tested, 10 formed TBPs with increased stability (see Table 1).

### Membrane Leakage and Simulated Pore Stability

We verified the simulations using an *in vitro* fluorescent dye leakage assay, which is commonly used to analyze the pore-forming activity of peptides.^29,58^ For these experiments, 22 mutants were selected independently of their TBP-stabilizing activity in the scaled Martini simulation in order to evaluate the relationship between the simulations and experiments (Table 2). The relationship between simulated TBP stability and leaky pore-forming activity appeared to be complex. Two of the four mutants with increased simulated TBP stability, LP26 and LP40, consistently caused higher leakage, whereas the other two, LP34 and LP36, caused less leakage than LP1. The lower leakage could be due to the narrower pore cavities observed in our simulations because of the tighter packing of T and S residues at the pore lumen. Tighter peptide packing due to hydrogen bonding networks of T and S is in line with a previous report.^39^ These results suggest that T/S mutations could be more selective for the transport of larger molecules through the pore and may have applications in nanobiotechnology for sensing and sequencing.^62,63^ Apart from the above correlation, 18 other mutants with equivalent or decreased TBP stability did not show a strong correlation between simulated TBP stability and leakage activity (Figure S12b). Moreover, several mutants caused significant leakage when there was no pore or when only closed pores were observed in the scaled Martini simulations (Table 2). Similarly, no correlation was found between the simulated TBP stability and antimicrobial activity (Figure S12c). Therefore, the scaled Martini force field seems to have underestimated the strength of the peptide–peptide interactions and TBP stability. Nevertheless, performing simulations with underestimated TBP stability facilitated identification of the pore-stabilizing mutations.

### Pore-Forming Ability and Micromolar Antimicrobial Activity

Only a weak correlation was found between the experimental leaky pore-forming activity and the *in vitro* antimicrobial activity (Table 2 and Figure S12d). However, with very few exceptions, antimicrobial activity within 1 μM concentration was always associated with a reasonable pore-forming activity, i.e., ≥50% leakage (Figure S12d). Other factors seem to play important roles in improving micromolar antimicrobial activity, even for those peptides that induced strong vesicle leakage. For example, LP40, which displayed increased TBP stability in simulations, induced both a higher leakage and higher antimicrobial activity. However, the antimicrobial activity was particularly high at pH 6 but not at the physiological pH of 7.4 (Figure 6a). Notably, in the leakage assay, LP40 was the most active pore-forming peptide at pH 7.4. The increase in antibacterial activity with decreasing pH may be related to the increase in peptide net charge from around +3 e to +10 e and to the related electrostatic attraction to the negatively charged bacterial cell membranes.^9,64,65^ Thus, the pore-forming ability is a necessary but not sufficient condition for killing bacteria, and AMP efficacy also needs a strong initial affinity for bacterial membranes. The fact that the most active peptide, LP18, showed both higher leakage and stronger antimicrobial activity supports this conclusion. The only difference between LP18 and LP1 was that LP18 has a +2 e higher net charge, which could plausibly have increased its affinity for bacterial membranes. Increased peptide affinity is also in line with more membrane defects and pores, as observed by AFM (Figure 5d). In summary, these results suggest that the stable TBP-forming ability is a crucial step, but not the limiting factor, in improving antimicrobial activity at peptide concentrations below one micromolar. Peptide affinity for the bacterial membrane, low barrier for insertion into the membrane, and low susceptibility to proteolytic degradation^9,10,23,31^ need to be tuned simultaneously.

### Antimicrobial Activity and Peptide Net Charge

There was no correlation between the peptide net charge and antimicrobial activity (Figure S12e), similar to dye leakage (Figure S12f). Peptides with the same net charge (+9 e) had bacterial MICs ranging from 0.2 to >50 μM and dye leakage ranging from zero to 90%. The most active peptide, LP18, had a net charge of +11, but peptide LP20, which has the same net charge, was less active. In addition, increasing the net charge further to +12 e was not found to increase activity, with a few exceptions (compare the antibacterial activity of LP28 with LP18). This observation is consistent with previous reports that increasing the net charge beyond a sequence-specific threshold might even decrease activity or selectivity.^9,11,23^ Such a threshold could be modulated by intramolecular pore-destabilizing interactions and/or too strong an affinity for the bacterial cell wall, limiting accessibility of the plasma membrane. Another possible cause is a higher barrier for the transmembrane insertion of the peptides or pore formation.^10,29,34,66,67^

### Nanomolar to Submicromolar Antimicrobial Activity against ESKAPEE Pathogens and Low Toxicity to Human Cells

We obtained the highest activity of LP1 with an MIC of 200 nM against *K. pneumoniae* ATCC 13883, a BSL-2 quality control strain. Some of the peptides derived from LP1 had higher *in vitro* antibacterial activity against the antibiotic-resistant ESKAPEE strains irrespective of the used protocol and strain resistance (Figure S3a). First, LP18, with an MIC of 500 nM, had four times higher activity than LP1 against a reference strain of *A. baumannii* (CIP 7010 or ATCC 15151). The activity of LP18 was equivalent to that of LP1 with MICs of 800 and 1000 nM against a clinically relevant strain of *A. baumannii* (ATCC 19606), a colistin- and meropenem-resistant clinical isolate of *A. baumannii* (GSAB 164) and a quality control strain (Seattle 1946) of *E. coli* (ATCC 25922). Second, LP28, with an MIC of 800 nM, was more active than LP1 against *E. faecium* (ATCC 700221; resistant to vancomycin), *P. aeruginosa* (PAO1 and PA14), and *E. coli* (ATCC 11775) and equally active against *A. baumannii* (ATCC 19606) and *E. coli* (AIC221). The micromolar activity of LP28 against *A. baumannii* (Z13), was four times higher than that of LP1. Third, LP35 had activity two times higher with an MIC of 500 nM against *E. coli* (E1098). These activities are higher than those of known AMPs, including indolicidin, HH2, Bac2A, guavanin-2, LDKA, YI12, FK13, interferon-I, anoplin, and temporin-PE;^58,68−73^ equivalent to those of synthetic and proline-rich peptides;^74,75^ and lower than those of arenicin-3.^76^

At the same time, the cytotoxic concentrations, i.e., IC50 values (with an average of 35 μM for all cell lines tested), of our highly active AMPs, LP1, LP18, LP28, and LP35, were sufficiently higher than the submicromolar bacterial MICs (i.e., MICs ranging from 0.2 to 1 μM). Therapeutic indices (calculated as the ratio of IC50 to MIC) ranged from 35 (using 35 μM IC50 and 1 μM MIC) to over 100 (for 0.2 μM MIC). Cytotoxicity was initially tested against human erythrocytes, skin keratinocytes, alveolar epithelial cells, and murine macrophages. These four AMPs were also found to be nonirritating to a reconstructed human skin model that mimics the biochemical and physiological properties of the outer layers of human skin. Note that the peptides could be degraded by host proteases (similar to bacterial proteases), resulting in less toxicity, irritation, and activity. Indeed, we have observed slow degradation in 25% human serum, leading to about 60% peptide remaining after 6 h of incubation and obtained no harmful side effects in whole blood experiments.

Antibacterial analysis of three cationic peptides, LP1, LP18, and LP28, against a panel of 33 ESKAPEE strains allows direct comparison of AMP activity against Gram-positive and Gram-negative bacteria. Overall, the peptides were more effective against Gram-negative than Gram-positive bacteria (Figure S13a). The lower activity against Gram-positive bacteria can be explained by the differences in the cell walls surrounding the plasma membrane of the two groups of bacteria. The peptidoglycan cell wall of Gram-positive bacteria is thicker and contains the negatively charged lipoteichoic acids,^77^ which together may prevent the peptides from reaching the membrane and forming pores. In addition, Gram-positive bacteria contain the positively charged lysyl-phosphatidylglycerol lipids in the plasma membranes, which have been reported to inhibit AMP activity.^78,79^ Nevertheless, the peptides showed submicromolar activity against Gram-positive *E. faecium*, but only against some specific strains, with MICs varying over an order of magnitude when considering all strains tested (Figure S13b). Similar variation in the MICs was observed against Gram-negative *K. pneumoniae* and *P. aeruginosa*. Such variation suggests an innate resistance of these bacteria to the cationic peptides, which was plausibly absent in Gram-negative *E. coli* and *A. baumannii*.

### Anti-Infective Activity in the Mouse Models of Gram-Negative Bacterial Infections

Due to the wide therapeutic window, our peptides are promising candidates for the treatment of skin and soft tissue infections caused by *A. baumannii*. The low hemolysis toxicity, optimal serum stability, and lack of deleterious side effects allow exploration of the other routes of administration. We investigated the efficacy of our AMPs for both topical and systemic applications using two mouse models: an excisional dorsal wound infection model and a deep thigh infection model. These are sensitive, standardized, and widely used preclinical models for evaluating the effects of antimicrobial compounds in complex living systems.^56,57,80^ In the wound model, the topically administered peptides demonstrated contractile healing of the *A. baumannii* wound infection under physiological conditions. The bacterial load did not decrease compared to that of the untreated control, suggesting peptide inactivation by pus or other wound factors that could induce peptide binding, aggregation, or degradation. Other topical formulations will be investigated in the future. In the deep thigh infection model, a single intraperitoneal dose of LP1 reduced the bacterial load of a clinically relevant *A. baumannii* infection by two to three orders of magnitude, demonstrating systemic antibiotic properties comparable to those of the conventional antibiotics polymyxin B and levofloxacin. The lower efficacy with increasing net charge of the peptides could be explained by their higher susceptibility to proteolytic degradation, which increased with the number of basic residues, common targets for serine proteases.^81^ It has also been reported that an increase in peptide net charge leads to a decrease in systemic *in vivo* efficacy in murine models.^82^ Nevertheless, our *in vivo* results set the stage for the future development of peptide-based drugs to treat infections caused by Gram-negative bacteria resistant to conventional antibiotics.

### pH-Sensitive Peptides with Anticancer Application

Cancer cell membranes, like bacterial cell membranes, have a net negative charge in the outer leaflet.^18−22,24,27,28^ Our cationic AMPs can selectively kill cancer cells by the TBP-forming mechanism because of their affinity for these membranes. Cancer cells are also surrounded by an acidic extracellular microenvironment,^83,84^ due to lactate secretion from anaerobic glycolysis. This acidic microenvironment could be exploited to increase the net charge of the peptides at low pH and enhance peptide selectivity for cancer cells.^25,26^ This approach could be applied to both solid tumors and blood cancers, as the microenvironment of lymph nodes, the main site of cell proliferation in leukemia and lymphoma (e.g., chronic lymphocytic leukemia), is acidic.^85^ In this context, the H residue has been shown to be beneficial,^86−89^ because it changes protonation state at around pH 6 (p*K*_a_ ∼ 6), i.e., it carries a +1 e charge at pH <6, is partially charged at pH ∼ 6, and is mostly neutral at pH >6.^42,43^ We rationally designed a H-variant peptide, LP40, by replacing the Ks at the peptide ends with Hs while leaving the Ks in the midsequence intact to preserve the TBP-stabilizing K-D salt bridges. As expected, the LP40 activity was found to be pH sensitive. A gain in activity against the cancer cells was observed when the pH of the medium was lowered from 8 to 6, similar to the pH-sensitive antibacterial activity. To the best of our knowledge, this is the first report of TBP-forming pH-sensitive antimicrobial and anticancer peptides.

## Conclusions

We used a combination of computer simulations and various *in vitro* experiments to show that the ability to form stable pores, although crucial for killing bacteria and cancer cells, is not the limiting factor in improving the antimicrobial and anticancer activity of our peptides below one micromolar concentration. Once the peptides were able to form stable pores, their affinity for bacterial and cancer cell membranes appeared to be the next property to optimize. Increasing the net charge of the peptides could improve activity while avoiding skin irritation and maintaining low toxicity to normal human cells. However, there is no linear relationship between the net charge and micromolar activity. A better understanding of affinity is needed to obtain peptides with antibacterial and anticancer activities in the nanomolar range. In addition, the interplay between sequence, affinity, and function in living systems appeared to be more complicated than in *in vitro* systems, as peptides may be susceptible to proteolytic degradation, a susceptibility that increases with net charge. Nevertheless, initial *in vivo* screening of the peptides in preclinical mouse models has demonstrated their anti-infective properties for systemic applications, comparable to polymyxin B and levofloxacin, suggesting that they may be promising AMP leads for the future development of AMPs for Gram-negative bacterial infections resistant to last-resort antibiotics. In addition, we have demonstrated the anticancer application of our *de novo* designed barrel-stave pore-forming peptides against a panel of cancer cell lines with pH-dependent activity. In summary, we have for the first time designed a class of novel synthetic peptides that form barrel-stave pores and exhibit potent anti-infective activity and pH-sensitive anticancer activity, further demonstrating the power of computers in antibiotic and chemotherapeutic discovery.

## Experimental Section

### Computer Simulations

MD simulations were performed with Gromacs program package (version 5.1.4.)^90^ using the CG Martini^91^ force field of two different types: the standard version 2.2^32^ and its modified “scaled” version in which Lennard-Jones interaction between peptides was uniformly decreased by 10%.^33^ The peptide structure was constructed in α-helical conformation using PyMOL^92^ and was converted to the CG model using martinize.py script.^32^ The helical secondary structure was maintained during the simulation by using dihedral angle restrains. Neutral peptide termini were used to mimic the acetylated N-terminus and amidated C-terminus by removing the charge and changing the backbone bead type of the first and last residues of the sequence, as recommended. The POPC bilayer consisting of 504 lipids was constructed in xy plane using the Martini Maker tool in CHARMM-GUI^93^ (http://www.charmm-gui.org).

A pore was created at the bilayer center by applying an inverted flat bottom potential (cylinder of radius 2 nm; force constant 1000 kJ mol^–1^ nm^–2^). Eight peptides were inserted perpendicularly (with respect to the membrane xy plane) in an antiparallel arrangement along the edge of the pore, with the peptide hydrophobic patches facing the lipid tails. The system was solvated with approximately 12,000 nonpolarizable standard Martini water beads, followed by the addition of Na^+^ and Cl^–^ ions to reach the physiological salt concentration of 0.154 M and for system neutralization. The system was energy minimized using steepest descent method and equilibrated in NPT ensemble as follows: (i) for 25 ps with 1 fs, (ii) for 250 ps with 5 fs, (iii) for 1 ns with 10 fs, (iv) for 5 ns with 20 fs, and (v) for 1 μs with 20 fs time step, in succession. During equilibration, the pore was held open using an inverted flat bottom potential. Subsequently, an unbiased production run of 51 μs with a 30 fs time step was performed.

Newton’s equations of motion were integrated using the leapfrog algorithm.^94^ Velocity rescale thermostat^95^ was used to maintain the temperature at 320 K with 1 ps coupling applied separately to protein–membrane and water–ion. Parrinello–Rahman barostat^96^ was used to maintain the pressure at 1 bar with semi-isotropic pressure coupling of 12 ps coupling constant and 3 × 10^–4^ bar^–1^ compressibility applied independently to the membrane plane and normal. Periodic boundary conditions were applied. The Verlet cutoff scheme^97^ with a 1.1 nm cutoff was applied. The Neighbor list was updated every 20 steps. van der Waals and Coulomb interactions were cutoff at 1.1 nm. The reaction field method^98^ was used to treat coulomb interactions with a relative dielectric constant of 15. The LINCS algorithm^99^ was used to constrain the bonds.

The ability of peptides to stabilize and relax the preformed pore (initially lined by the lipid heads) into a regular barrel-stave pore structure^9,10,29^ was investigated using VMD (version 1.9.3.)^100^ to generate the snapshots. The average number of water beads inside the pore was calculated within a 2 nm vertical distance from the membrane center of mass. A pair of interacting residues (charged or aromatic) was considered to be in the interaction contact (salt bridge or stacking) if the pairwise distance was less than 0.8 nm, which is the position of the first minima in radial distribution functions.^29^ The average number of interaction contacts over a 51 μs simulation was calculated as a percentage of the designed number of contacts. An interaction was considered to be stable when the contact occurred for at least 50% of the simulation time.

### Chemicals

Peptides were synthesized from CASLO ApS (Denmark), and phospholipids were purchased from Avanti Polar Lipids (USA). Cell lines were obtained from the following resources: HaCaT cells from AddexBio (USA), A549 cells and RAW 264.7 macrophages from ATCC (USA), epidermis test kit EpiDerm EPI-200-SIT (containing tissues, cultivation medium, MTT Assay Kit) from MatTek In Vitro Life Science Laboratories (Slovakia), and cancer cells from the German Collection of Microorganisms and Cell Cultures (DSMZ). Other chemicals were acquired from the following resources: NaH_2_PO_4_·H_2_O, NaCl, and NaOH from Merck (Germany); Tris–HCl from Roche Diagnostics (Germany); Mueller–Hinton Broth (MHB) and Mueller–Hinton Agar (MHA) powders from Condalab (Spain); Na_2_HPO_4_·7H_2_O, EDTA, chloroform, ethanol, and methanol from VWR (USA); calcein, CaCl_2_, KCl, and Triton X-100 from Sigma-Aldrich (USA); DiD from Life Technologies (USA); RPMI from Gibco Thermo Fisher (USA); DMEM from Biosera (France); and human and murine cell culture materials from Corning (USA). Peptides were dissolved in phosphate-buffered saline (PBS), and phospholipids and DiD were dissolved in chloroform and stored at −20 °C. Bacteria were stored at −80 °C. Unless stated otherwise, buffer/media pH was adjusted to 7.4. Experiments were performed in triplicates.

### Dye Leakage Assay

Pore-forming activity was tested using a dye leakage assay measuring the leakage of self-quenching fluorescent dye calcein from POPC:POPG (1:1 mol/mol) LUVs. DiD was mixed with lipids (1:500 mol/mol), and lipid film was created by evaporating chloroform, followed by 4 h of desiccation. Dried lipid film was dissolved in calcein buffer (35 mM calcein, 25 mM NaH_2_PO_4_·H_2_O and Na_2_HPO_4_·7H_2_O (3:7 mol/mol), 20 mM NaCl, and 1 mM EDTA). After 10 freeze–thaw cycles, the sample was extruded 50 times through a 100 nm pore size polycarbonate membrane filter. Free calcein in solution was removed using a HiTrap Desalting column. The concentrations of LUVs and peptides were adjusted to 0.02 and 0.1 mM, respectively, in PBS (25 mM NaH_2_PO_4_·H_2_O and Na_2_HPO_4_·7H_2_O (3:7 mol/mol), 100 mM NaCl, and 1 mM EDTA). The investigated P/L ratio was from 1:100 to 1:10 (mol/mol). Fluorescence intensity was measured using a HORIBA Scientific Jobin Yvon FluoroLog-3 Modular Spectrofluorometer (USA). Excitation and emission wavelengths were set to 495 and 520 nm, respectively. Triton X-100 10% caused 100% leakage used for normalization.

### Antimicrobial Susceptibility Assay

Antimicrobial activity against *E. coli* TOP10 and *S. carnosus* CCM 4838T was tested using the broth microdilution assay. Bacteria were cultured in MHA Petri dish for 24 h, subcultured in an MHB test tube for 6 h at 37 °C, and diluted to 10^6^ CFU/mL (OD_600_). Stock solution of test compounds was prepared in PBS (1.8 mM NaH_2_PO_4_·H_2_O, 10 mM Na_2_HPO_4_·7H_2_O, 135 mM NaCl, and 4.5 mM KCl). Dilution series was prepared for concentrations 50 to 0.1 μM in a flat-bottom 96-well plate, inoculated with bacteria, and incubated for 24 h at 37 °C. Growth control wells were free from test compounds, and sterility control wells were free from bacteria. Bacterial MIC was determined as the lowest concentration of test compounds that inhibited visible growth of bacteria (i.e., no visible turbidity).

### Antimicrobial Susceptibility Assay against ESKAPEE Pathogens

(Protocol a) Antimicrobial activity test against ESKAPEE strains *S. aureus* NRS 1, *K. pneumoniae* ATCC 700603, *A. baumannii* GSAB 164, *P. aeruginosa* NCTC 13437, and *E. coli* ATCC 25922 was purchased from Evotec (UK). The protocol was similar to only a few exceptions: *E. faecium* was recovered on Columbia blood agar; buffer mixture of 1% (v/v) DMSO and 0.1 M PBS; bacteria ∼2–8 × 10^5^ CFU/mL; round-bottom well plate; the highest tested concentration of peptides ∼15 μM; incubation for 18 and 20 h for *A. baumannii*; and MIC was additionally confirmed by OD_600_.

(Protocol b) Test against ESKAPEE strains *E. faecalis* JH2-2 and UCN41, *E. faecium* ATCC 19434T and BM4147, *S. aureus* ATCC 25923, and *A. baumannii* CIP7010, was performed using a similar protocol with only a few exceptions: cation-adjusted MHB was used; bacterial inoculum was prepared directly from the 0.5 McFarland (McF)-adjusted colony suspension, diluted 1:20 to a final concentration of 5 × 10^5^ CFU/mL; the highest tested concentration of peptides ∼30 μM; incubation for 16 to 20 h at 35 ± 2 °C; and the quality control strain of *P. aeruginosa* (ATCC 27853) was used as the control using ceftazidime as the reference antibiotic.

(Protocol c) Test against ESKAPEE strains *E. faecium* Z906, *S. aureus* ATCC 29213, *S. epidermidis* 30 WT, *K. pneumoniae* E1120, E1267, and 4371, *A. baumannii* Z13, *P. aeruginosa* K11 and K12 and *E. coli* E1098, *S. aureus* protease mutants: NE1506, NE934, NE163, and NE1740,^49^ protease null mutant ANG 2038 (AH1919)^50^ and control strains ANG 1575 (AH1263),^101^ and *S. aureus* USA 300 derivative JE2 was performed using a similar protocol with only a few exceptions: bacteria were cultured for 18 h; several colonies were scraped and resuspended in 0.9% NaCl to 10^8^ CFU/mL (0.5 McF); the highest tested concentration of peptides ∼30 μM (128 mg/L); the MIC was considered as the concentration that reduced ≥80% bacterial growth (OD_600_) compared to control.

(Protocol d) Test against ESKAPEE strains *E. faecium* ATCC 700221 (vancomycin-resistant), *E. faecalis* ATCC 700802 (vancomycin-resistant), *S. aureus* ATCC 12600 and ATCC BAA-1556 (methicillin-resistant), *K. pneumoniae* ATCC 13883, *A. baumannii* ATCC 19606, *P. aeruginosa* PAO1 and PA14, *E. coli* ATCC 11775, and AIC221 and AIC222 (colistin-resistant) was performed using a similar protocol with only a few exceptions: bacteria ∼2 × 10^6^ cells mL^–1^, Luria–Bertani (LB) medium, and 20 h incubation before the visual assessment of MIC.

### Hemolysis Assay

Hemolysis toxicity was tested against HRBCs. Blood samples, collected from the university hospital, were centrifuged twice at 700 × *g* and once at 1000 × *g* for 8 min at 4 °C to obtain erythrocyte pellets. Supernatants were always discarded, and pellets were resuspended in PBS (the same as the antimicrobial susceptibility assay). The final RBC suspension was 0.5% (v/v). Dilution series was prepared for concentrations 50 to 0.1 μM in a V-bottom 96-well plate, followed by the addition of RBC suspension. Negative control wells were free from peptides (0% hemolysis), and positive control wells contained 5 μM melittin (100% hemolysis). After 1 h of incubation at 37 °C, the well plate was centrifuged at 1000 × *g* for 10 min at 4 °C. The supernatant, collected in a flat-bottom well plate, was measured for OD_415_ using Tecan Microplate Reader Infinite F500. The normalized absorbances of the released hemoglobin were used to calculate IC50 value, i.e., the concentration of peptides causing 50% hemolysis.

### MTT Assay

Cytotoxicity against human immortalized keratinocytes HaCaT, human alveolar basal epithelial A549 (derived from adenocarcinoma), and murine macrophages RAW 264.7 was tested using MTT [3-(4,5-dimethylthiazol-2-yl)-2,5-diphenyltetrazolium bromide] assay. Cells were cultured in Dulbecco’s modified Eagle’s medium (DMEM) containing 4 mM glutamine for HaCaT, or 2 mM glutamine for A549 and RAW 264.7 cells, supplemented with 10% heat-inactivated fetal bovine serum (FBS) and antibiotics (0.1 mg/mL penicillin and streptomycin). Nonessential amino acids (NEAA) and sodium pyruvate (1 mM) were added for macrophages. Cell lines were grown in 25 and 75 cm^2^ flasks at 37 °C in a humidified atmosphere with 5% CO_2_. Cells were suspended in DMEM, containing glutamine, NEAA, and sodium pyruvate (as described above), supplemented with 2% FBS, plated in 96-well plates at 4 × 10^4^ cells/well, and incubated at 37 °C in a 5% CO_2_ atmosphere. After overnight incubation, the medium was replaced with fresh DMEM supplemented with peptides at different concentrations: 2, 4, 8, 16, 32, 64, and 128 μM. Negative controls were cells without peptides. After 24 h of peptide treatment at 37 °C in a 5% CO_2_ atmosphere, medium was replaced with Hank’s buffer (136 mM NaCl, 0.34 mM Na_2_HPO_4_, 0.44 mM KH_2_PO_4_, 5.4 mM KCl, 4.1 mM NaHCO_3_, supplemented with 5.5 mM d-glucose) containing 0.5 mg/mL MTT. Mitochondrial dehydrogenases converted soluble yellow dye MTT to insoluble purple formazan. After 4 h of incubation, acidified isopropanol was added to dissolve formazan crystals and measured for OD_570_ using Tecan microplate reader Infinite M200 (Salzburg, Austria). OD values were expressed as percentage cell viability compared to negative controls and used to calculate IC50 values, i.e., the concentration of peptides inhibiting 50% cell growth.

### Skin Irritation Assay

Skin toxicity against reconstructed human epidermis, composed of functional epidermis and stratum corneum, was tested at the National Institute of Public Health (SZU, Czech Republic) following MTT-based OECD TG 439 guidelines. Tissues were transferred into a six-well plate containing assay medium and incubated for 1 h in the humidified incubator Heracell at 37 °C in 95% humidity and 5% CO_2_. Tissues were transferred into a new six-well plate with fresh medium and incubated overnight. After incubation, tissues were separately exposed to 30 μL of 50 μM peptides, PBS alone (negative control), and 5% SDS (positive control) for 1 h. Tissues were rinsed with PBS, blotted, dried with cotton swab, and incubated for 42 h. Tissues were placed into MTT medium for 3 h and rinsed with PBS, and the formazan crystals were extracted using isopropanol for 2 h. Aliquots of 3 × 200 μL were transferred to a 96-well plate and measured for OD_570_ using BioTek microplate reader EON. OD values were expressed as percentage tissue viability compared to negative control. According to UN GHS Category 2, peptides were considered to be skin irritants if tissue viability was ≤50% and considered to have “no category” (i.e., not skin irritant) if tissue viability was >50%.

### CellTiter-Glo Luminescent Assay

The CellTiter-Glo luminescent cell viability assay examining the impact of peptides on 22 different human cell lines was purchased from Reaction Biology Europe GmbH (Germany). Cells were cultured in different media (Table S1), seeded into a flat-bottom 96-well plate, and incubated at 37 °C. After overnight incubation, test compounds were added with 1% PBS/0.3% Tween 20 and the reference compound bortezomib was added with 0.1% DMSO. Negative controls were solvents alone, and positive control was 10 μM staurosporine. After 72 h of incubation at 37 °C, 5 or 10% CO_2_ (dependent on the medium), and 1 h equilibration at room temperature, well plates were treated with CellTiter-Glo reagent (Promega) for an hour and luminescence was measured using a luminometer. Raw data were expressed as percentage cell viability relative to controls and used to calculate IC50 values, i.e., the concentration of compounds inhibiting 50% cell growth.

### Viability by WST Assay

Anticancer activity of peptides against acute myeloid leukemia MOLM-13, chronic lymphocytic leukemia MEC-1, and diffuse large B-cell lymphoma SU-DHL-4 cell lines was tested using a WST cell viability assay. Cells were cultured in 75 cm^2^ culture flasks at 37 °C in 5% CO_2_ according to the DSMZ recommendations before the start of the experiment. Then, the cells were seeded into 96-well plates in quadruplicate at 2.5 × 10^4^ cells/well for SU-DHL-4, and 5 × 10^4^ cells/well for MEC-1 and MOLM-13. Cells were treated with either peptides or PBS alone (negative control) for 24–72 h at 37 °C in 5% CO_2_. After incubation, cell viability was measured by WST-1 (Roche) according to the manufacturer’s instructions using Tecan microplate reader Infinite M200 PRO Plex and expressed as percentage compared to control.

### Viability by Flow Cytometry DiOC6/7AAD Staining Assay

Effects of pH and culture medium on the anticancer activity of peptides against acute myeloid leukemia HL-60, chronic myeloid leukemia K-562, breast adenocarcinoma MCF-7, and colon cancer cells HCT-116 cultured in three different media RPMI (#21875034, Gibco), DMEM (#LM-D1110/500, Biosera), and PBS adjusted to three different pH values 8, 7, and 6, and a medium without pH adjustment (∼7.6), were tested using flow cytometry DiOC6/7AAD (3,3-dihexyloxacarbocyanine iodide/7-amino-actinomycin D) staining assay, as described previously.^102^ Media pH was adjusted twice, i.e., 1 day before the experiment and immediately before the experiment. Medium was supplemented with 10% heat-inactivated FBS, glutamine, sodium pyruvate, and antibiotics (100 U/mL penicillin and 100 μg/mL streptomycin). Cells were cultured in 75 cm^2^ culture flasks at 37 °C in 5% CO_2_ according to the DSMZ recommendations before the start of the experiment. In the case of HL-60 and K-562 cells, 3 × 10^5^ cells/well were seeded in 24-well plates and treated with 10 μM peptides or vehicle control (PBS) for the indicated time periods. In the case of HCT-116 and MCF-7 cells, 10^5^ cells/well were seeded in 24-well plates 1 day before the experiment; the next day, the cells were treated with 10 μM peptides or vehicle control (PBS) for the indicated time periods. To determine cell viability, cells were incubated with DiOC_6_ (Thermo Fisher Scientific) and 7AAD (Thermo Fisher Scientific) for 15 min at 37 °C and cell viability was measured using a BriCyte E6 flow cytometer (Mindray); viable cells were considered as DiOC_6_-positive and 7AAD-negative.

The effect of pH on peptide activity in cancer cells was evaluated by the multiway ANOVA followed by Tukey post hoc test. Regarding the comparison of the effect of the medium, we analyzed the data from Figure 6c by comparing RPMI medium and DMEM of the same pH, and statistical significance was assessed using the multiway ANOVA followed by Tukey post hoc test. The effect of peptides was compared using multiway ANOVA followed by Tukey post hoc test. Statistical evaluation and graphs were performed using Statistica 14 (StatSoft) and GraphPad Prism 8 (GraphPad Software).

### Viability by Flow Cytometric Staining in Whole Blood

The whole blood was seeded in a 96-well plate at 200 μL/well and treated with peptides for 16 h. The samples were then stained with anti-CD3 (FITC, Sony), anti-CD14 (APC-Cy7, Sony), and anti-CD19 (PE, Beckman Coulter) antibodies and incubated for 20 min at room temperature in the dark. The cells were then incubated with red blood cell lysis buffer for 10 min at room temperature, centrifuged, resuspended in PBS containing SYTOX Blue Dead Cell Stain (Thermo Fisher Scientific) and measured using a FACSVerse flow cytometer (BD Biosciences).

### Immunoblotting

MCF-7 cells were seeded at 2.5 × 10^5^ cells/well in a 24-well plate 1 day before the experiment. Cells were treated with either peptides or PBS alone (negative control) and cultured in different media for 1 h. Cells were lysed using a buffer consisting of 1% SDS, 50 mM Tris–HCl (pH 6.8), and 10% glycerol with phosphatase and protease inhibitors. The protein concentration was determined by using the DC Protein Assay (Bio-Rad). Proteins were separated by SDS-PAGE and transferred to the PVDF membrane with a 0.45 μm pore size. The membranes were incubated with primary antibodies to detect cleaved PARP (no. 5625, 1:1000, Cell Signaling), total PARP (no. 9542, 1:2000, Cell Signaling), cleaved caspase-3 (no. 9664, 1:1000, Cell Signaling), or β-actin (no. 4970, 1:2000; Cell Signaling). Secondary HRP-linked antirabbit antibody (1:2000, Cell Signaling) was used to detect primary antibodies. Signal was detected using Clarity Western ECL Substrate (BioRad) and UVITEC Alliance (Uvitec).

### Cytoplasmic Membrane Depolarization Assay

The cytoplasmic membrane depolarization activity of the peptides was determined by measuring the fluorescence of membrane-potential-sensitive dye DiSC_3_-(5). *A. baumannii* ATCC 19606, *E. coli* ATCC 11775, *K. pneumoniae* ATCC 13883, *P. aeruginosa* PAO1, *S. aureus* ATCC 12600, and *E. faecium* ATCC 700221 were grown to mid log phase (OD_600_ = 0.5) at 37 °C and then centrifuged (10,000 rpm at 4 °C for 10 min). Cells were washed twice with a buffer containing 20 mM glucose and 5 mM HEPES (pH 7.2) and diluted 1:10 (OD_600_ = 0.05) in a buffer with 0.1 M KCl, 20 mM^1^ glucose, and 5 mM HEPES (pH 7.2). Fluorescence was recorded at the excitation and emission wavelengths of 622 and 670 nm, respectively. Bacterial solution (100 μL) was incubated for 15 min with 20 nM of DiSC_3_-(5) until fluorescence reached a plateau. Changes in transmembrane potential were monitored by observing the difference in fluorescence emission intensity after adding peptides (100 μL at their MICs) for 45 min. Assay was performed in triplicate.

### Outer Membrane Permeabilization Assay

The outer membrane permeabilization activity of the peptides was determined using the NPN uptake assay. *A. baumannii* ATCC19606, *E. coli* ATCC 11775, *K. pneumoniae* ATCC 13883, and *P. aeruginosa* PAO1 were cultured to an optical density (OD_600_) of 0.4 and then centrifuged (10,000 rpm at 4 °C for 10 min), washed, and resuspended in a buffer containing 5 mM HEPES and 5 mM glucose (pH 7.4). NPN solution (4 μL of 10 mM) was added to 100 μL of the bacterial solution in a white 96-well plate. Fluorescence was recorded at the excitation and emission wavelengths of 350 and 420 nm, respectively. Peptides (100 μL at their MICs) were added to the well plate, and fluorescence was recorded for 20 min after reaching a plateau. Assay was conducted in triplicate.

### Peptide Stability in Human Serum Assay

To assess the peptide resistance to proteolytic degradation, we incubated the peptides (at 10 mg mL^–1^) in 25% human serum (in water) for 6 h at 37 °C. Aliquots (100 μL) were collected after 0, 0.5, 1, 3, and 6 h, and proteins from the serum were precipitated with 10 μL of 100% trifluoroacetic acid (TFA) and incubated for 10 min on ice. Samples were then processed in a Waters ACQUITY UPLC-MS equipped with a photodiode array detector (190–400 nm data collection) and a Waters TQD triple quadrupole MSMS, with 5 μL injections. The column used was a Waters ACQUITY UPLC HSS C_18_, 1.8 μm (2.1 mm × 50 mm). The mobile phases used were A (100% water with 0.1%, v/v, formic acid) and B (100% acetonitrile with 0.1%, v/v, formic acid), Fisher optima grades. Gradient is shown in Table 3. Measurements were made by ionization ESI ± simultaneous over *m*/*z* 100–2000 Da. The percentage of remaining peptide was calculated by tracking the most abundant ion related to the peptide and comparing it to its abundance at time point zero. Experiments were performed in three independent replicates.

**Table 3:** Mobile Phase Gradient Used to Assess the Resistance of Peptide to Proteolytic Degradation

time (minutes)	A (%)	B (%)	flow rate (mL min^–1^)
0	95	5	0.5
0.5	95	5	0.5
2.5	5	95	0.5 (linear gradient)
3	5	95	0.5
3.25	5	95	0.5

### Neutropenic Murine Thigh Infection Model

Six-week-old female CD-1 mice (obtained from Charles River; stock number 18679700-022) underwent neutropenia induction via two intraperitoneal doses of cyclophosphamide (150 mg kg^–^^1^) administered with a 72 h interval. One day later, the mice received an intramuscular injection containing *A. baumannii* ATCC 19606 (10^6^ CFU mL^–1^) in the right thigh. Bacteria were cultured in LB broth, washed twice with PBS (pH 7.4), and resuspended to the desired concentration. Two hours postinfection, peptides were administered (LP1:0.16 mg/kg, LP18:0.165 mg/kg, LP28:0.176 mg/kg, LP40:1.33 mg/kg) intraperitoneally to the mice. Polymyxin B (0.006 mg/kg) and levofloxacin (0.014 mg/kg) were used as the reference antibiotics. Prior to each injection, mice were anesthetized with isoflurane and their respiratory rate and pedal reflexes were monitored. Subsequently, we observed the infection establishment and euthanized the mice. The infected area was excised 4 days postinfection (i.e., 8 days after the beginning of the experiment), homogenized using a bead beater for 20 min (25 Hz), and 10-fold serially diluted for CFU quantification on MacConkey agar plates. Each experimental group comprised three mice. All experiments were conducted blindly, and no animals were excluded from the analysis. Statistical significance was assessed using one-way ANOVA followed by Dunnett’s test on log_10_-transformed data to mitigate outlier effects; *p* values are reported for each group, with all groups compared to the untreated control group. The experiment was approved by the University Laboratory Animal Resources (ULAR) at the University of Pennsylvania (Protocol 807055).

### Murine Excisional Dorsal Wound Infection Model

Eight-to-ten-week-old female Balb/c mice (obtained from Envigo, Netherlands) were subjected to immunosuppression induction by two intraperitoneal doses of 150 and 100 mg/kg cyclophosphamide on days −4 and −1, respectively, before wounding. On day 0 of wounding, mice were anesthetized with isoflurane and the dorsal region was shaved and scrubbed with iodine solution. A full-thickness skin defect was created over the thoracic spine using a 6 mm disposable skin biopsy punch (KAI Industries Co., Ltd., Japan). Infection was established by applying 25 μL of *A. baumannii* NCTC 13301 (5 × 10^4^ CFU/mL) applied directly to the wound and allowed to absorb for 2 min. Wound was covered with a transparent sterile dressing (Tegaderm, Deutschland GmbH, Germany) and secured with tissue adhesive (Surgibond, SMI AG, Belgium). Afterward, mice received an intramuscular injection of buprenorphine (0.05 mg/kg). Starting 4 h postinfection, mice were treated once daily for 7 days as follows: groups 1 and 2 received two different peptides at 1000 times their MICs dissolved in 20 μL of PBS (∼10 mg/kg), group 3 was the control group treated with only 20 μL of PBS, and group 4 was the control group without any treatment. Each group comprised 10 mice. The solution was applied directly to the wound by puncturing the dressing with a sterile Hamilton syringe. On day 7, treatment was discontinued and dressing was removed. To monitor the wound size/closure, caliper-based measurements were performed. Photographs of the wound were taken using a 5-megapixel camera (Samsung, South Korea) starting from the day of wounding and at subsequent time points until a complete wound closure was achieved. To assess the number of viable bacterial cells in the wound, one mouse from each group was euthanized by cervical dislocation under isoflurane anesthesia on days 1, 3, and 7 postinfections. Wound bed material and the surrounding tissues were excised, weighed, placed in 1 mL sterile PBS, mechanically homogenized (ULTRA-TURRAX T 10 basic, IKA-Werke GmbH & Co. KG, Germany) and serially diluted 10-fold. From 10^–3^, 10^–5^, and 10^–7^ dilutions, 100 μL of the sample was collected three times, incubated on MacConkey agar plates (Trios, Czech Republic), and analyzed for viable cell counts. The experiment was conducted in accordance with the regulations and guidelines of the Czech Animal Protection Act (No. 246/1992) with the approval of the Czech Ministry of Education, Youth, and Sports MSMT-2963/2023-5 and the Institutional Animal Welfare Committee of the Faculty of Medicine and Dentistry, Palacký University Olomouc.

### AFM

Effects of peptides on the SLBs composed of POPC:POPG (1:1 mol/mol) lipids was investigated using AFM. LUVs were prepared in lipid dissolution buffer (50 mM Tris–HCl and 50 mM KCl) following a dye leakage assay protocol without calcein. Vesicles were sonicated for 30 min at 25 °C and extruded through a 50 nm pore size filter to form small unilamellar vesicles (SUVs). Following the vesicle deposition method,^103^ SLBs were formed on mica using SUVs in SLB-forming buffer (50 mM Tris–HCl and 300 mM KCl) and 5 mM CaCl_2_, mounting a total volume of 150 μL on mica. After 1 h incubation at 25 °C, mica was washed 10 times with SLB-forming buffer and subjected to AFM analysis. The investigated P:L ratio was 1:500 (mol/mol). Bruker Dimension Icon AFM (USA) equipped with a PeakForce-HIRS-F-B silicon nitride probe (spring constant 0.12 N/m) was used to capture topographic images. PeakForce Quantitative NanoMechanic hybrid mode was used with 2 kHz frequency and 25 nm amplitude using set point <100 pN. The scan rate was 1 Hz using 512 samples/line. Gwyddion software (version 2.58)^104^ was used for line-by-line background subtraction (flattening) and plane fitting of the images.

## Supporting Information Available

Simulated water density profiles through the pores, interaction strengths, *in vitro* antimicrobial, hemolysis, serum stability, *in vivo* wound healing, *in vitro* anticancer results, correlation study, and supplementary materials (PDF)
Molecular formula strings (CSV)

## Abbreviations

AFM: atomic force microscopy
AMP: antimicrobial peptide
CFU: colony forming unit
CG: coarse-grained
DiSC_3_-(5): 3,3′-dipropylthiadicarbocyanine iodide
HPLC: high-performance liquid chromatography
HRBC: human red blood cell
IC50: half-maximal inhibitory concentration
LP: long pore-forming peptide
LUV: large unilamellar vesicle
MD: molecular dynamics
MIC: minimum inhibitory concentration
NPN: 1-(*N*-phenylamino) naphthalene
P:L: peptide-to-lipid ratio
POPC: 1-palmitoyl-2-oleoyl-*sn*-glycero-3-phosphocholine
POPG: 1-palmitoyl-2-oleoyl-*sn*-glycero-3-phospho-1′-rac-glycerol
SLB: supported lipid bilayer
TBP: transmembrane barrel-stave pore
