# Supplementary material for: Computational Design of Pore-Forming Peptides with Potent Antimicrobial and Anticancer Activities
Source: J Med Chem. 2024 Aug 8;67(16):14040–61. doi: 10.1021/acs.jmedchem.4c00912 (PMC11345766; doi:10.1021/acs.jmedchem.4c00912)
Supplement: Supplementary file 1 — jm4c00912_si_001.pdf [file jm4c00912_si_001.pdf]

Supplementary information for

Computational design of pore-forming peptides with potent antimicrobial and anticancer activities

Rahul Deb[1,2], Marcelo D. T. Torres[3,4,5,6], Miroslav Boudný[1,7], Markéta Kobĕrská[8], Floriana Cappiello[9], Miroslav Popper[10], Kateřina Dvořáková Bendová[10], Martina Drabinová[1], Adelheid Hanáčková[1], Katy Jeannot[11,12*], Miloš Petřík[10,13*], Maria Luisa Mangoni[9*], Gabriela Balíková Novotná[8*], Marek Mráz[1,7*], Cesar de la Fuente-Nunez[3,4,5,6*], and Robert Vácha[1,2,14*]

[1]CEITEC − Central European Institute of Technology, Masaryk University, Brno 625 00, Czech Republic;
[2]National Centre for Biomolecular Research, Faculty of Science, Masaryk University, Brno 625 00, Czech Republic;
[3]Machine Biology Group, Departments of Psychiatry and Microbiology, Institute for Biomedical Informatics, Institute for Translational Medicine and Therapeutics, Perelman School of Medicine, University of Pennsylvania, Philadelphia, Pennsylvania 19104, USA;
[4]Departments of Bioengineering and Chemical and Biomolecular Engineering, School of Engineering and Applied Science, University of Pennsylvania, Philadelphia, Pennsylvania 19104, USA;
[5]Penn Institute for Computational Science, University of Pennsylvania, Philadelphia, Pennsylvania 19104, USA;
[6]Department of Chemistry, School of Arts and Sciences, University of Pennsylvania, Philadelphia, Pennsylvania 19104, USA;
[7]Department of Internal Medicine, Hematology and Oncology, University Hospital Brno and Faculty of Medicine, Masaryk University, Brno 625 00, Czech Republic;
[8]Institute of Microbiology, Czech Academy of Sciences, BIOCEV, Vestec 252 50, Czech Republic;
[9]Department of Biochemical Sciences, Laboratory Affiliated to Istituto Pasteur Italia-Fondazione Cenci Bolognetti, Sapienza University of Rome, Rome 00185, Italy;
[10]Institute of Molecular and Translational Medicine, Faculty of Medicine and Dentistry, Palacký University, Olomouc 779 00, Czech Republic;
[11]University of Franche-Comté, CNRS, Chrono-environment, Besançon 25030, France;
[12]National Reference Centre for Antibiotic Resistance, Besançon 25030, France;
[13]Czech Advanced Technology and Research Institute, Palacký University, Olomouc 779 00, Czech Republic;
[14]Department of Condensed Matter Physics, Faculty of Science, Masaryk University, Brno 611 37, Czech Republic.

*Corresponding author emails -
Robert Vácha: robert.vacha@muni.cz
Cesar de la Fuente-Nunez: cfuente@upenn.edu
Marek Mráz: marek.mraz@email.cz
Gabriela Balíková Novotná: gnovotna@biomed.cas.cz
Maria Luisa Mangoni: marialuisa.mangoni@uniroma1.it
Miloš Petřík: milos.petrik@upol.cz
Katy Jeannot: katy.jeannot@univ-fcomte.fr

Table of contents **Pages**

Supplementary Tables

Table S1. *In vitro* activity against 22 different human cell lines……………………………..........S3

Supplementary Figures

Figure S1. Water channels through the stabilized pores in 'scaled' Martini simulations……….S4

Figure S2. Intramolecular salt bridge formation at the transmembrane peptide ends in pores….S5

Figure S3. *In vitro* antimicrobial activity……………………………………………………….S6

Figure S4. *In vitro* toxicity against human red blood cells………………………………........S8

Figure S5. *In vitro* stability in human serum ……………………………………………........S9

Figure S6. *In vivo* antimicrobial and wound-healing activity in mice…………….………........S10

Figure S7. *In vitro* dye leakage activity…………………………………………..…............S16

Figure S8. *In vitro* anticancer activity………………………………......…………………..S17

Figure S9. Effect of media on anticancer activity………………………………...................S18

Figure S10. Effect of pH and media on anticancer activity……………………….....................S19

Figure S11. Flow cytometric analysis of whole blood……………………...............................S20

Figure S12. Correlations between pore stability, *in vitro* activity, and peptide net charge.........S21

Figure S13. Comparison of antimicrobial activity against Gram-positive and Gram-negative bacteria...... …………………........................………………...S22

Supplementary Materials

HPLC and MS certificates of the synthesized peptides………………….......................S23 – S70

Supplementary Tables

Table S1. ***In vitro*** **activity against 22 different human cancer cell lines.**

Sr. no.	Cell line	Cancer type	Culture medium	Activity (in M)		
				LP1	LP40	Bortezomib
1	A498	Kidney	DMEM + 10% FCS	3.6E-06	2.0E-05	9.1E-09
2	A549	Lung	DMEM + 10% FCS	8.4E-06	9.7E-05	2.5E-08
3	Caki-1	Kidney	DMEM + 10% FCS	6.6E-06	2.3E-05	1.2E-08
4	DU-145	Prostate	DMEM + 10% FCS	5.9E-06	6.0E-05	1.6E-08
5	H460	Lung	DMEM + 10% FCS	3.7E-06	> 2.0E-04	2.6E-08
6	HCT116	Colorectal	DMEM + 10% FCS	7.1E-06	7.9E-05	6.9E-09
7	HCT-15	Colorectal	RPMI-1640 +10% FCS	4.7E-06	7.1E-06	9.8E-09
8	HL-60	Leukemia	RPMI-1640 + 10% FCS	6.5E-06	7.2E-06	4.1E-09
9	K562	Leukemia	RPMI-1640 + 10% FCS	6.4E-06	6.4E-06	9.1E-09
10	MCF-7	Breast	DMEM + 10% FCS	8.3E-06	7.0E-05	2.1E-08
11	MDA MB 231	Breast	DMEM + 10% FCS	6.7E-06	6.1E-05	1.2E-08
12	MDA MB 435	Melanoma	RPMI-1640 +10% FCS	1.4E-06	1.9E-04	4.3E-09
13	MOLT-4	Leukemia	DMEM + 10% FCS	1.6E-05	> 2.0E-04	4.0E-09
14	MSC_UC	Non-cancerous	Promocell MSC-Medium C-28010 + Sup. C39810	2.0E-06	1.4E-04	4.7E-09
15	NHDF	Non-cancerous	Promocell NHDF-Medium C-23010 + Sup. C39315	7.2E-06	> 2.0E-04	1.4E-08
16	OVCAR-3	Ovarian	RPMI-1640 +10% FCS	4.9E-06	9.0E-06	4.0E-09
17	PC3	Prostate	DMEM + 10% FCS	1.6E-05	9.1E-05	1.6E-08
18	RPMI 8226	Myeloma	RPMI-1640 +10% FCS	4.4E-06	5.6E-06	3.2E-09
19	SK-OV3	Ovarian	DMEM + 10% FCS	1.3E-05	1.1E-04	1.9E-08
20	SW620	Colorectal	DMEM + 10% FCS	2.9E-05	1.4E-04	1.0E-08
21	T-47D	Breast	RPMI-1640 +10% FCS + 0,2U/mL Insulin	3.2E-06	4.6E-06	8.6E-09
22	U251MG	Brain	DMEM + 10% FCS	2.0E-05	> 2.0E-04	1.9E-08

The effect of investigated compounds on the proliferation of 22 different human cell lines is reported as the half maximal inhibitory concentration, i.e., IC50 value, after 72 hours of incubation. Twenty of these 22 cell lines represent specific cancer types and 2 were non-cancerous. Peptide sequences are provided in Table 1 in the main text. Anticancer drug bortezomib was used as the reference.

Supplementary Figures

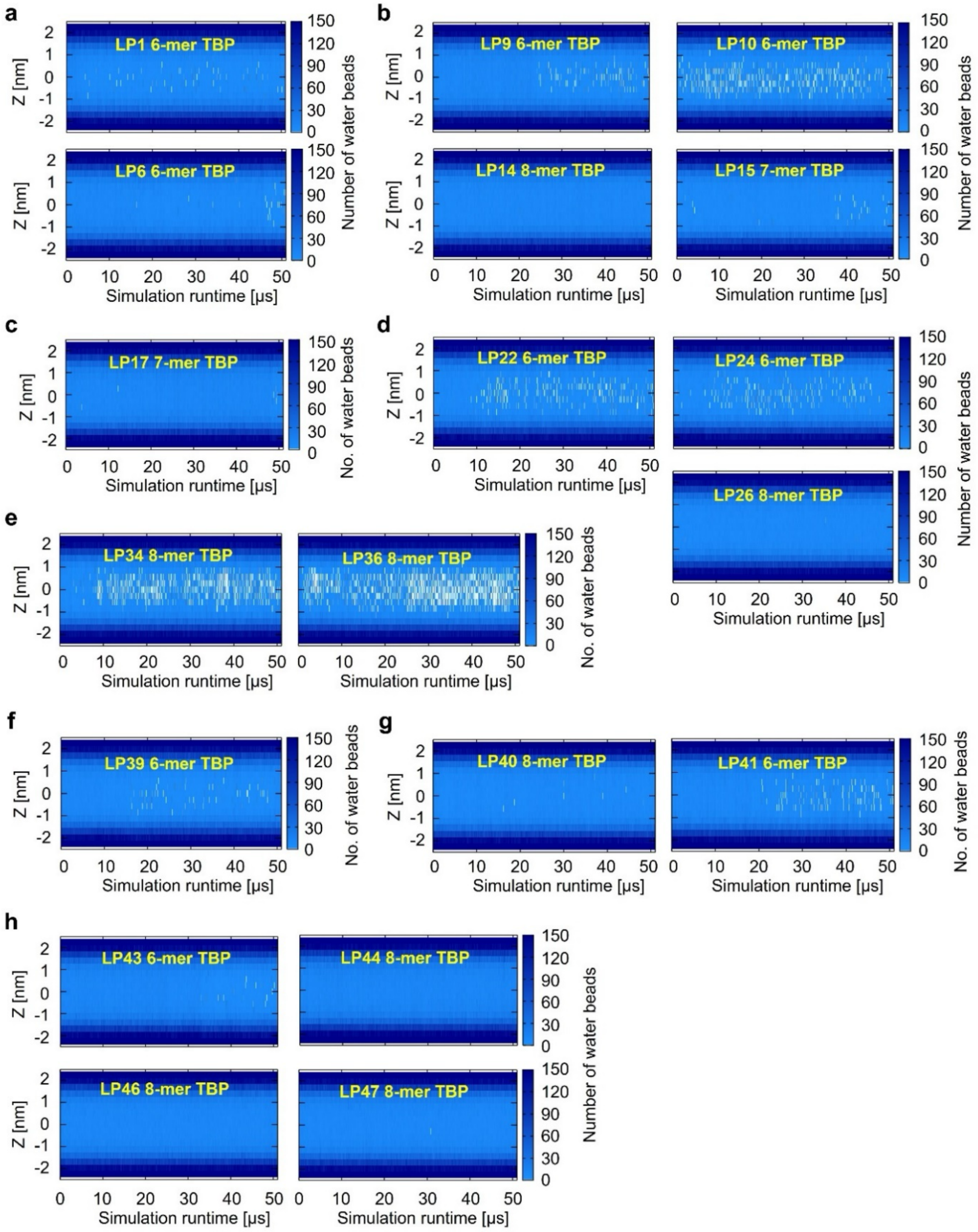

Figure S1. Water channels through the stabilized pores in 'scaled' Martini simulations. Water density profiles were calculated as the average number of CG water beads passing through the transmembrane barrel pores (TBPs) stabilized by the peptides over 51 μs long MD simulations using 'scaled' Martini force field (see Table 1 in the main text).

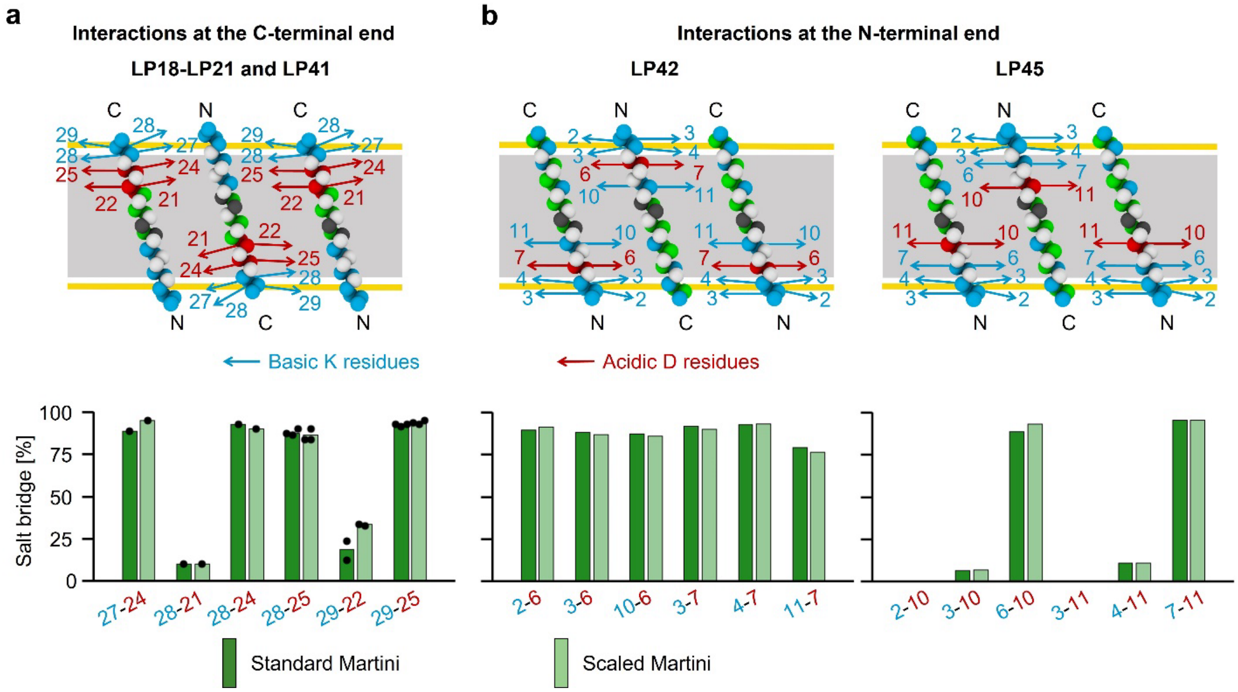

Figure S2. Intramolecular salt bridge formation at the transmembrane peptide ends in pores. Schematic illustrations of the formation of intramolecular salt bridges at the peptide ends and the strength of such interactions in the transmembrane barrel pores. Illustrations depict three antiparallel neighboring transmembrane peptides showing two representative peptide-peptide interfaces from an octameric transmembrane barrel pore (in the side view). The stability of these interactions was calculated as the percentage of designed interaction contacts averaged over 51 µs simulation run using the standard and 'scaled' Martini force fields. Color coding: peptide hydrophilic and hydrophobic residues in green and white, respectively; basic and acidic in residues blue and red, respectively; aromatic residues in grey; yellow horizontal lines demarcate the position of lipid phosphates; and grey panel represents the lipid tails. Peptide sequences are provided in Table 1 in the main text.

a

Species	Strain	Antibiotic resistance / strain details	LP1	LP18	LP28	AMC	POL	LEV	Prot.
E. faecalis	JH2-2	WT	>32	>32	>32	0.7	>25	1.4	b
E. faecalis	UCN41	VAN	>32	>32	>32	1.4	>25	1.4	b
E. faecalis	ATCC 700802	VAN	>60	>60	>60		>60	0.8	d
E. faecium	ATCC 19434T	WT	>32	15	28	1.4	>25	6	b
E. faecium	BM4147	AMX, VAN	16	4	7	>23	>25		b
E. faecium	Z906	VAN, TEI, LNZ	>32	16	4	>360			c
E. faecium	ATCC 700221	VAN, TEI	12.5	>60	0.8		>60	50	d
S. aureus	ATCC 25923	WT	>8	>8	>8	1.4	>25	3	b
S. aureus	NRS1	MRSA or VISA	>16	>16	>16	46			a
S. aureus	ATCC 29213	Clinical	>32	>32	>32	46			c
S. aureus	ATCC 12600	MSSA	50	50	25		50	1.6	d
S. aureus	ATCC BAA-1556	MET	>60	>60	12.5		>60	3.1	d
K. pneumoniae	ATCC 700603	ESBL	4	4	7	>180			a
K. pneumoniae	E1120	GEN	8	8	2	>360			c
K. pneumoniae	4371	GEN, AMI, IMI, MER	16	8	8	>360			c
K. pneumoniae	E1267	GEN, AMI	>32	>32	>32	>360			c
K. pneumoniae	ATCC 13883		0.2	0.8	0.8		0.1	0.4	d
A. baumannii	CIP 7010	WT	2	0.5	2	>92	1.5	0.3	b
A. baumannii	GSAB 164	COL, MER	1	1	2	>183			a
A. baumannii	Z13	GEN, AMI, IMI, MER	4	8	1	>360			c
A. baumannii	ATCC 19606		0.8	0.8	0.8		0.1	0.8	d
P. aeruginosa	NCTC 13437	ESBL	>16	11	11	>180			a
P. aeruginosa	K12	AMI, IMI, MER, CTZ, PIT	>32	>32	8	>360			c
P. aeruginosa	K11	IMI, MER, CTZ, PIT	>32	16	8	>360			c
P. aeruginosa	PA01	WT	3.1	3.1	0.8		0.1	6.3	d
P. aeruginosa	PA14	WT	3.1	3.1	0.8		0.4	0.8	d
E. coli	ATCC 25922	WT	1	1	3	23			a
E. coli	E1098	GEN, AMI, IMI, MER	1	2	4	>360			c
E. coli	ATCC 11775		1.6	1.6	0.8		0.1	0.2	d
E. coli	AIC221		0.8	1.6	0.8		0.2	0.4	d
E. coli	AIC222	COL	1.6	1.6	1.6		0.2	0.4	d

b

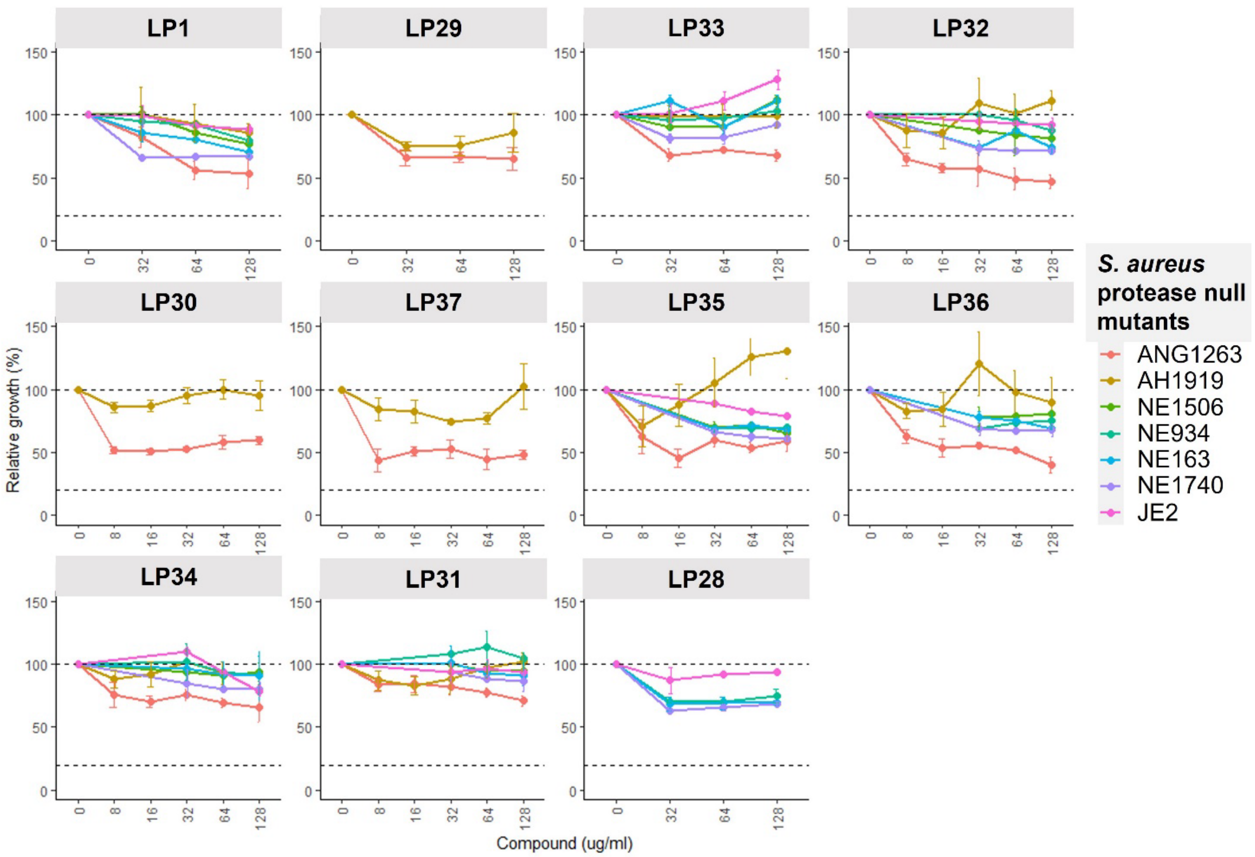

Figure S3. ***In vitro*** **antimicrobial activity.**

(a) Minimum inhibitory concentrations (MICs in μM) of selected peptides and reference antibiotics (AMC, ampicillin; POL, polymyxin B; LEV, levofloxacin) against different ESKAPEE pathogens. The third column indicates either antibiotic resistance or strain details as follows: WT, wild type; VAN, vancomycin; AMX, amoxicillin; TEI, teicoplanin; =LNZ, linezolid; MET, methicillin; GEN, gentamicin; COL, colistin (polymyxin E); MER, meropenem; AMI, amikacin; IMI, imipenem; CTZ, chloramphenicol-tetracycline-zinc eugenol oxide; PIT, piperacillin; CTL, ceftezole; MSSA, methicillin-susceptible *Staphylococcus aureus*; MRSA, methicillin-resistant *Staphylococcus aureus*; VISA, vancomycin-Intermediate *Staphylococcus aureus*; ESBL, extended-spectrum beta-lactamases. The MICs were obtained using four slightly different protocols, labelled a-d (see Experimental Section in the main text). (b) Antimicrobial activity against *S. aureus* null mutant strains. The plots depict growth of protease single mutants NE163 (USA300 JE2 aur::Tn::Erm), NE 1506 (USA300 JE2 sspA::Tn::Erm), NE934 (USA300 JE2 sspB::Tn::Erm), NE1740 (USA300 JE2 scpA::Tn::Erm) and protease null mutant strain AH1919 (LAC* Δ*aur* Δ*sspAB* Δ*scpA* spl::erm) together with control strains ANG1263 (ANG1575) and *S. aureus* USA300 (after the peptide treatment relative to the bacterial growth without peptides) as a function of peptide concentrations. The highest tested peptide concentration of 128 μg/mL corresponds to ~ 30 μM and that of the reference antibiotic ampicillin corresponds to ~ 366 μM. Peptide sequences are provided in Table 1 in the main text.

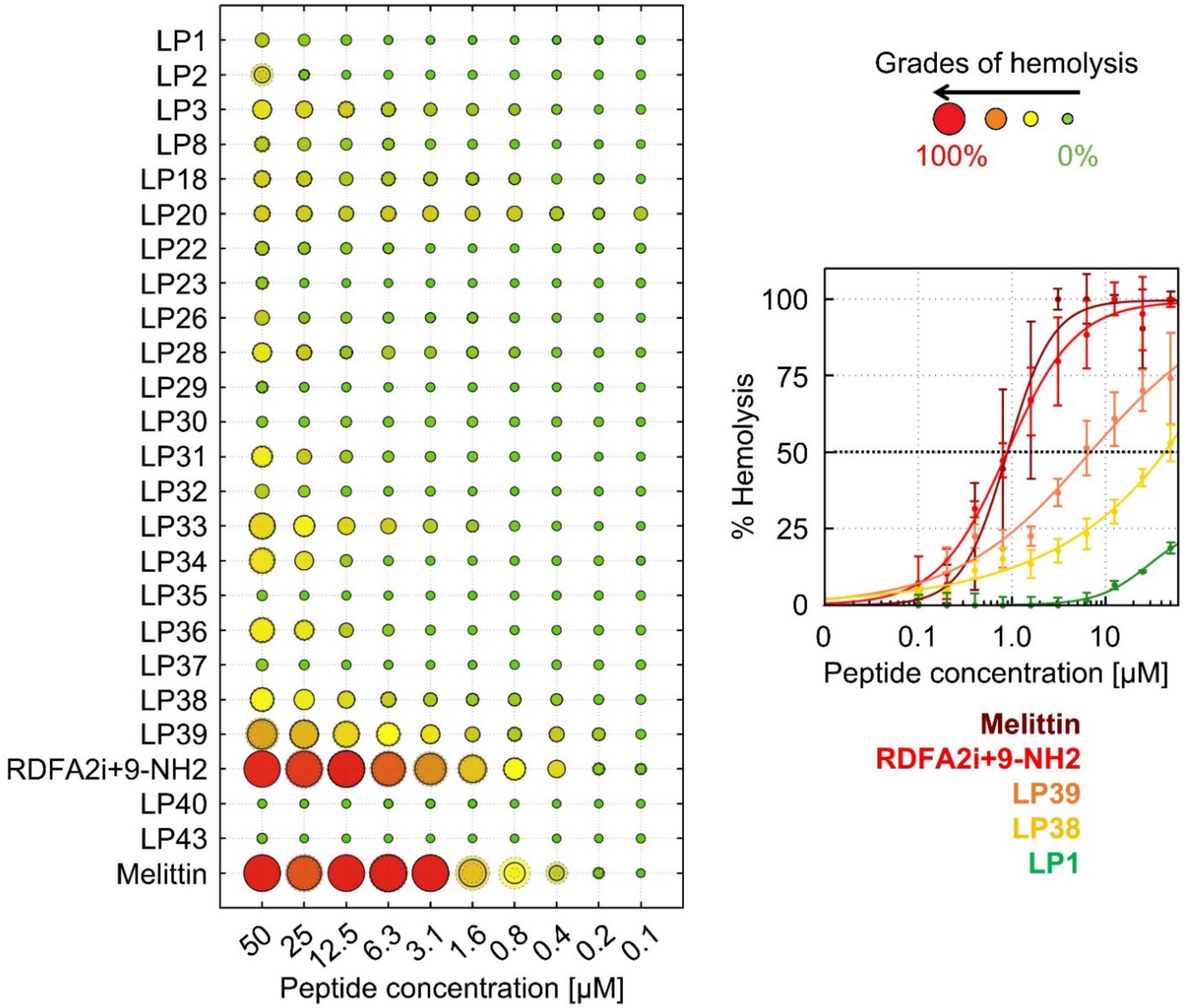

Figure S4. ***In vitro* toxicity against human red blood cells.**
(**Left**) Dilution series demonstrating peptide toxicity against the human red blood cells as a function of peptide concentration. Grades of hemolysis are as follows: red (100% hemolysis), orange to yellow (intermediate hemolysis), and green (0% hemolysis). Solid circles represent averages over triplicate measurements and dashed circles represent standard deviations. (**Right**) The normalized hemolysis plot, indicating the toxic peptide concentration that caused 50% lysis of human cells, i.e., IC50 values. Peptide sequences are provided in Table 1 in the main text. Hemolytic peptide melittin was used as the reference.

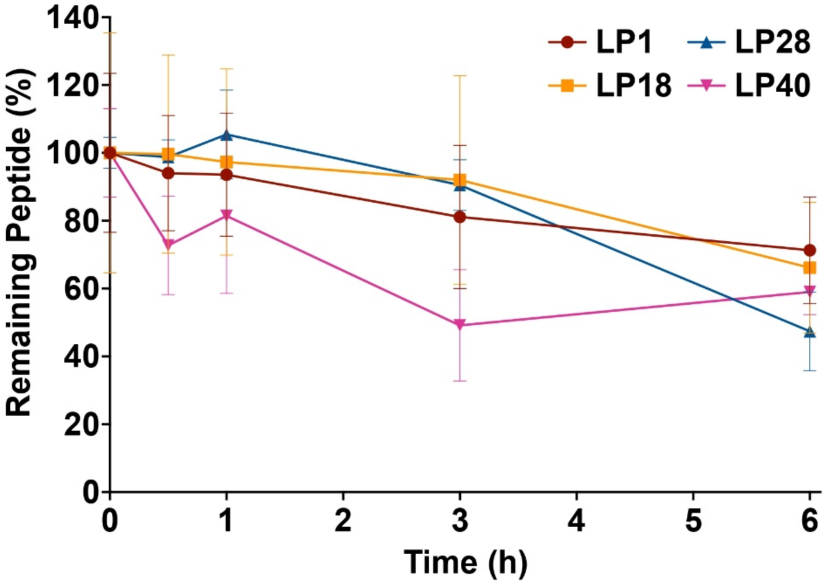

Figure S5. *In vitro* stability in human serum.

Peptide resistance to proteolytic degradation was assessed in 25% human serum and reported as the percentage of peptides remaining at various intervals over 6 hours. Peptide sequences are provided in Table 1 in the main text.

a

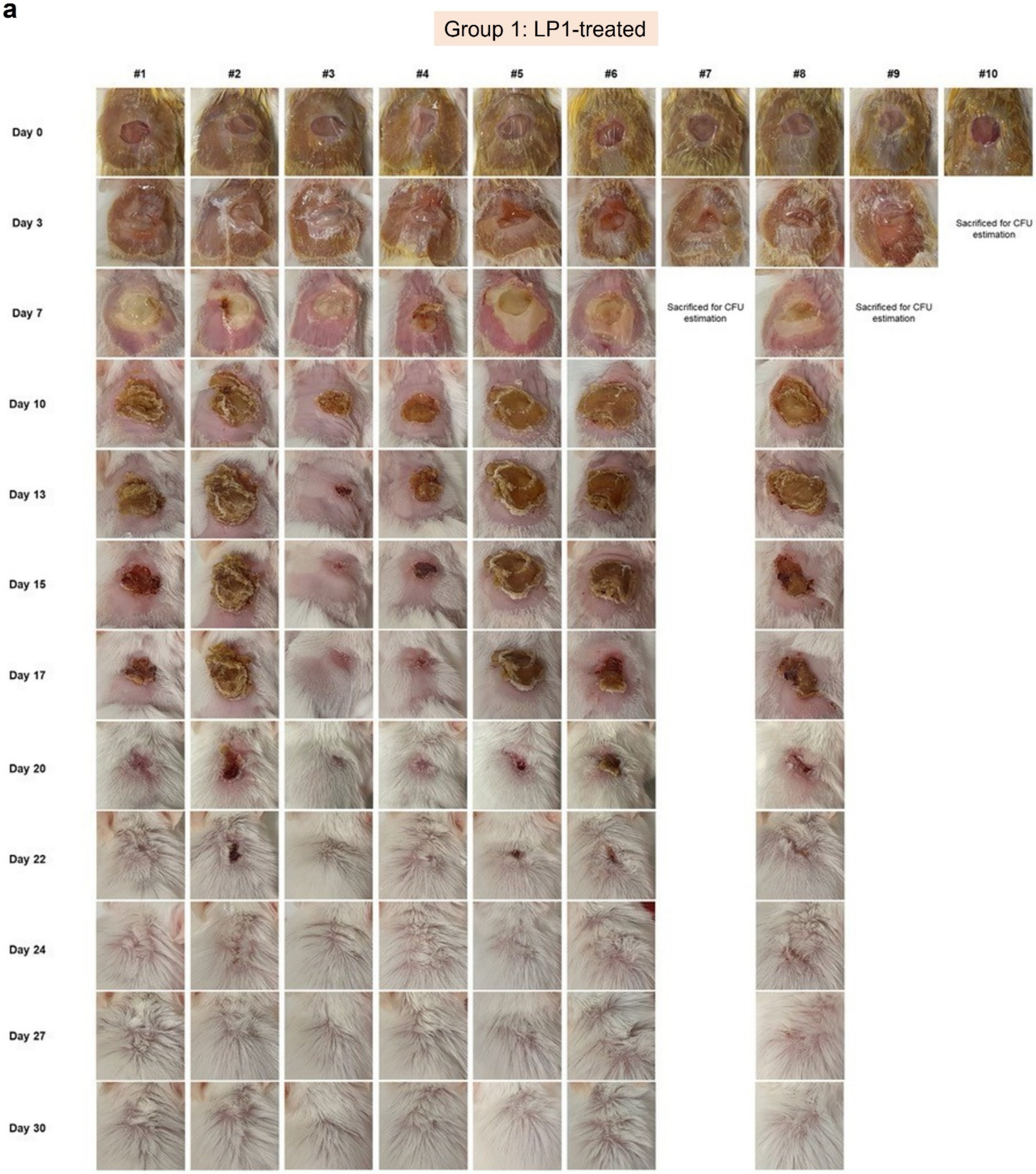
Group 1: LP1-treated
#1
#2
#3
#4
#5
#6
#7
#8
#9
#10
Day 0
Day 3
Day 7
Day 10
Day 13
Day 15
Day 17
Day 20
Day 22
Day 24
Day 27
Day 30
Sacrificed for CFU estimation
Sacrificed for CFU estimation
Sacrificed for CFU estimation

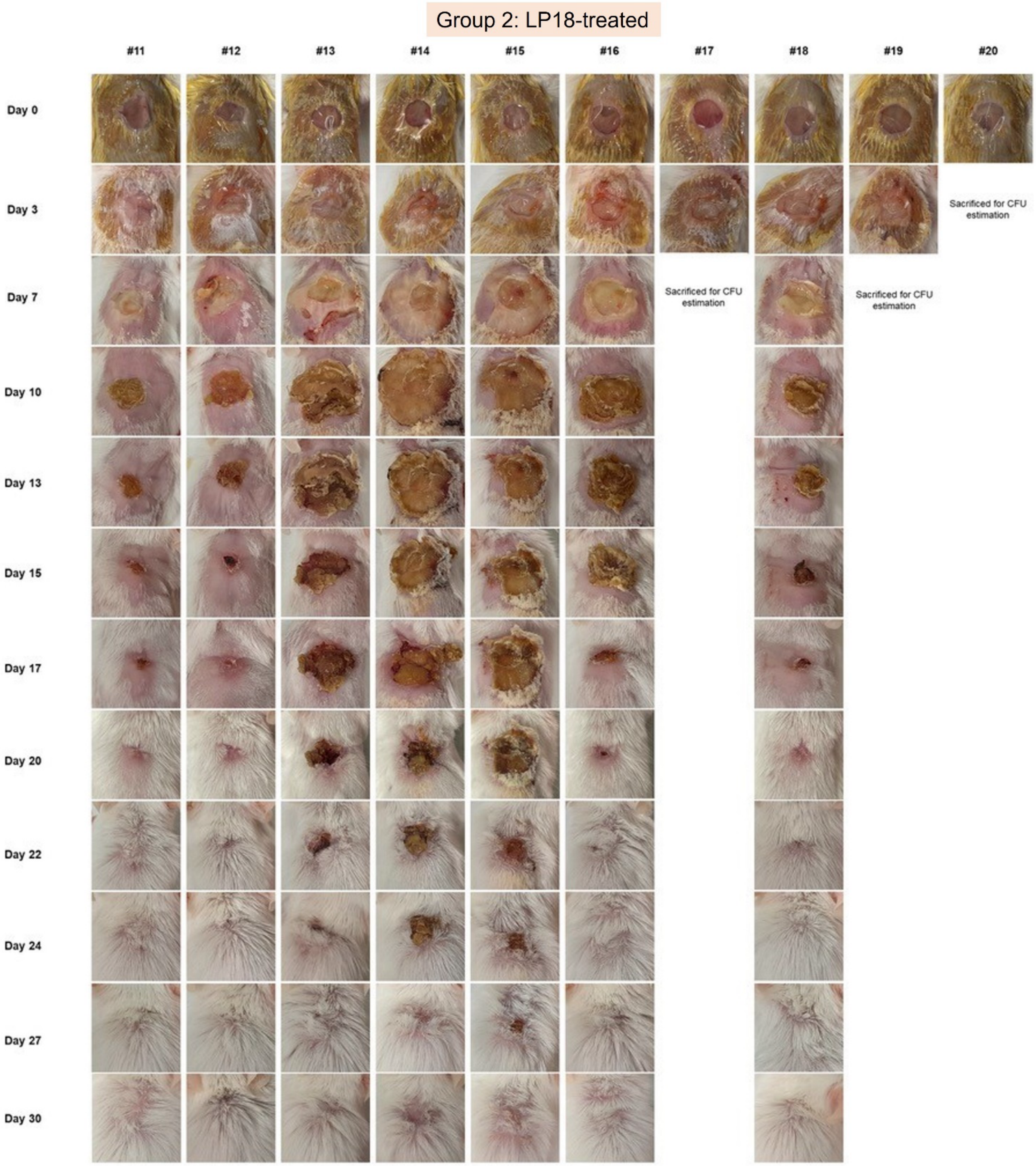

Group 2: LP18-treated
#11
#12
#13
#14
#15
#16
#17
#18
#19
#20
Day 0
Day 3
Day 7
Day 10
Day 13
Day 15
Day 17
Day 20
Day 22
Day 24
Day 27
Day 30
Sacrificed for CFU estimation
Sacrificed for CFU estimation
Sacrificed for CFU estimation

Group 3: PBS-treated

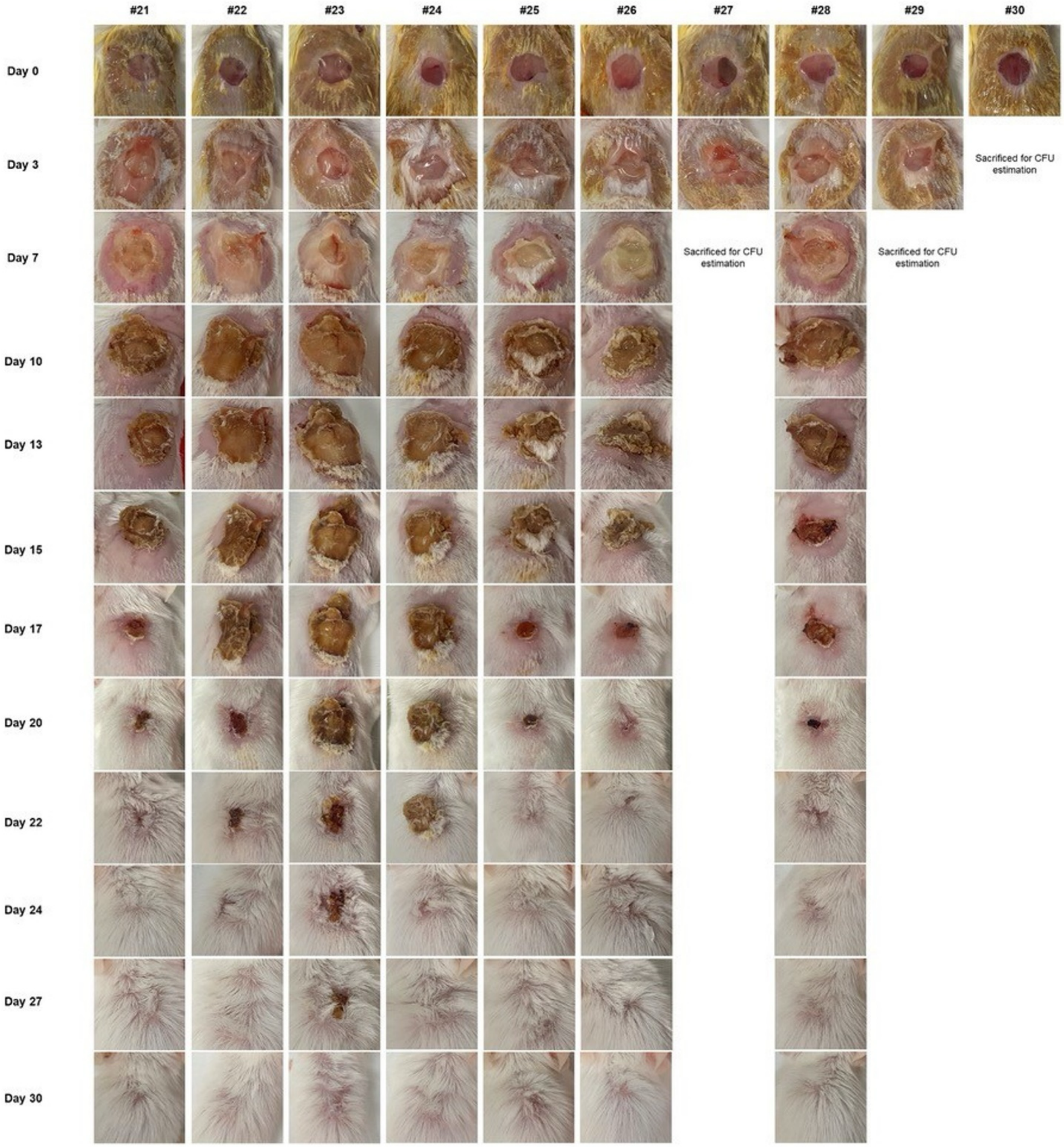

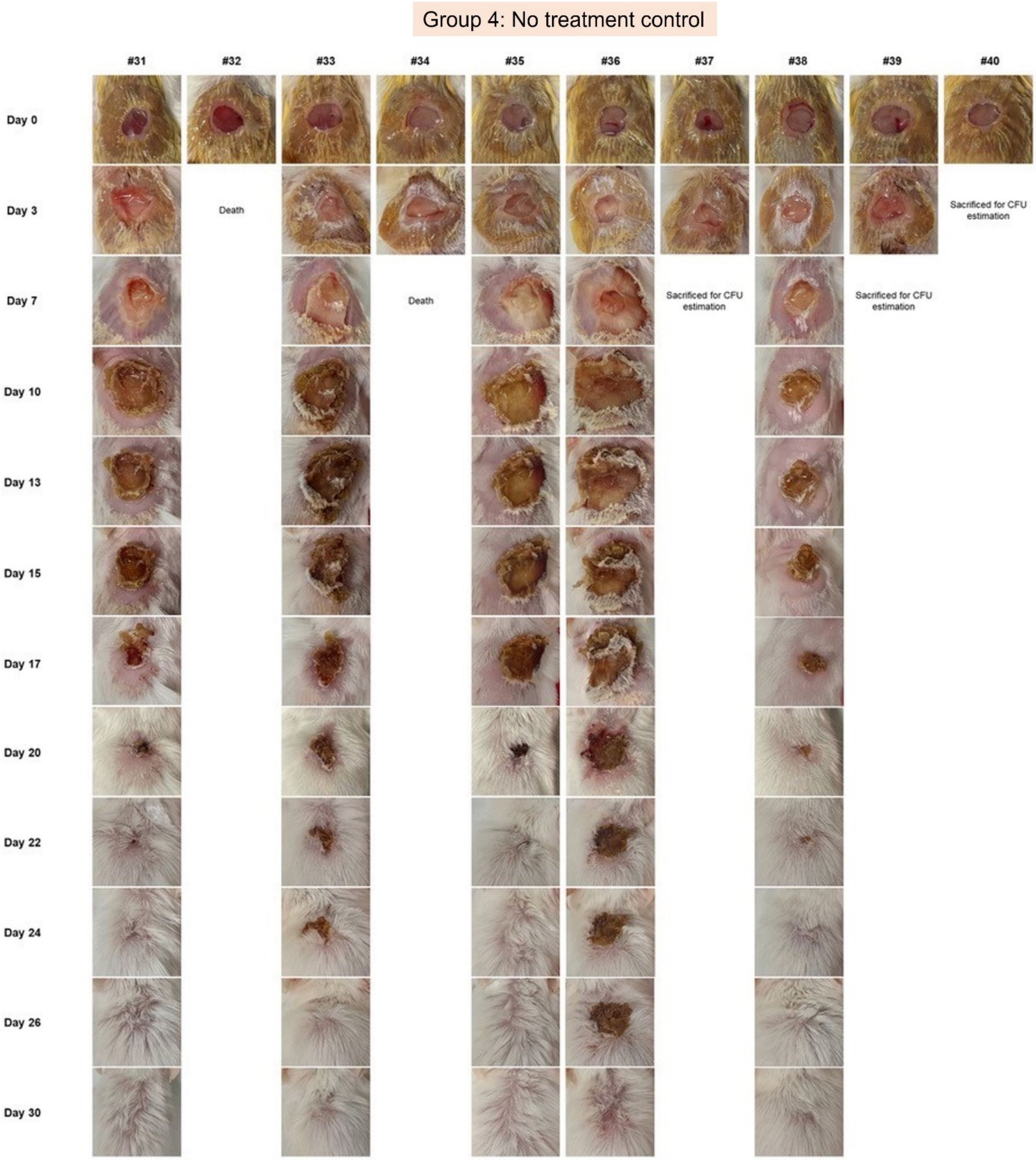
Group 4: No treatment control
#31
#32
#33
#34
#35
#36
#37
#38
#39
#40
Day 0
Day 3
Day 7
Day 10
Day 13
Day 15
Day 17
Day 20
Day 22
Day 24
Day 26
Day 30
Death
Sacrificed for CFU estimation
Death
Sacrificed for CFU estimation
Sacrificed for CFU estimation

b

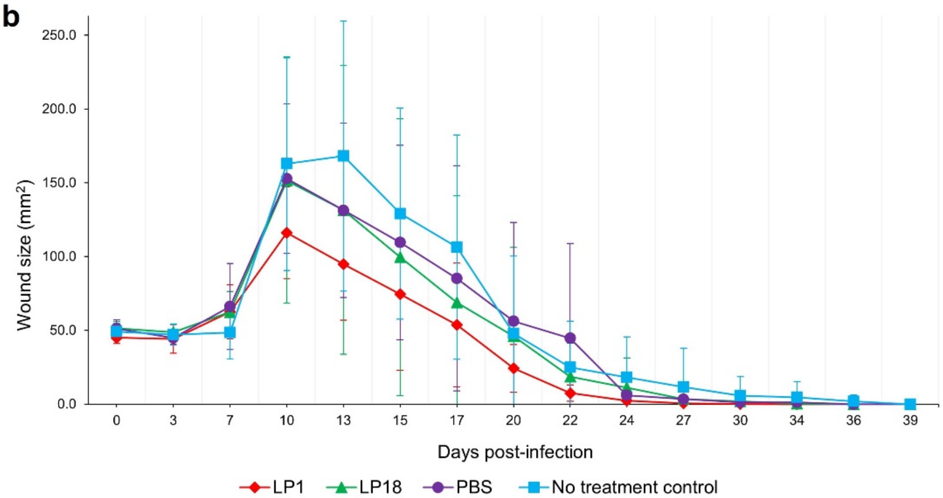
Wound size (mm²)
Days post-infection
LP1
LP18
PBS
No treatment control

c

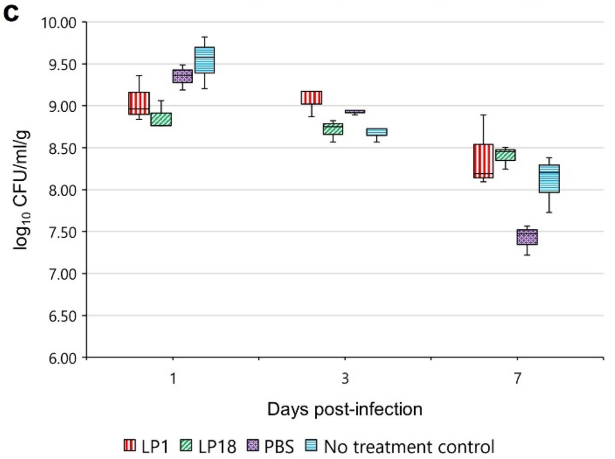
log10 CFU/ml/g
Days post-infection
LP1
LP18
PBS
No treatment control

d

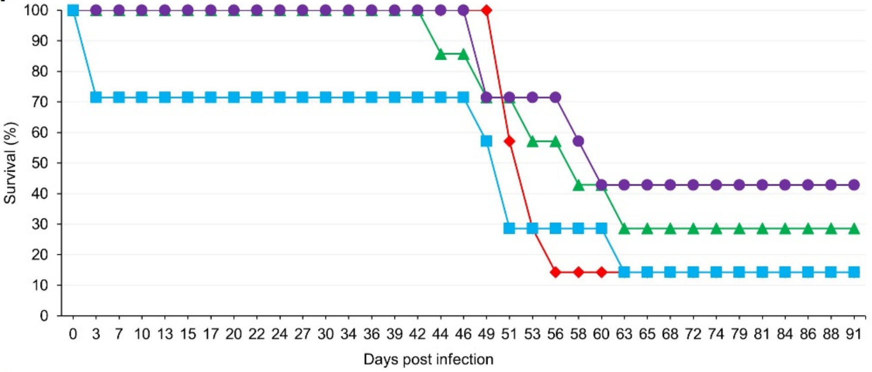
Survival (%)
Days post infection

e

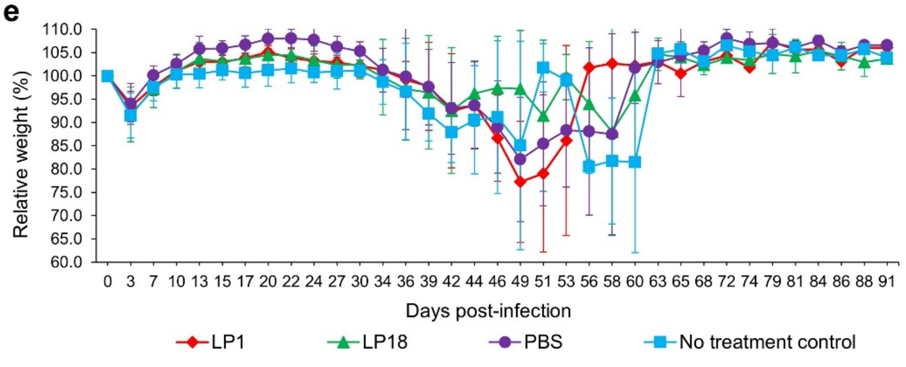
Relative weight (%)
Days post-infection
LP1
LP18
PBS
No treatment control

Figure S6. ***In vivo*** **antimicrobial and wound-healing activity in mice.**
(a) Excisional dorsal wounds were photographed with a 5-megapixel camera from the day of wounding (i.e., day 0) and on subsequent days until a complete wound closure was observed. (b) Wound size was measured using calipers based on the height and width of the wounds. (c) Box-and-whisker plots show the CFU tissue load, normalized to mouse weight, recovered from the wound beds on days 1, 3, and 7 postinfection. Boxes show medians and interquartile ranges, and whiskers show the lowest and highest values not classified as outliers. (d) Survival rate and (e) the relative change in body weight over the course of the experiment. Peptides were applied directly to the wound by puncturing the dressing with a sterile Hamilton syringe at 10000-fold MICs (i.e., 2.43 mM for LP1, and 0.95 mM for LP18). Peptide sequences are provided in Table 1 in the main text.

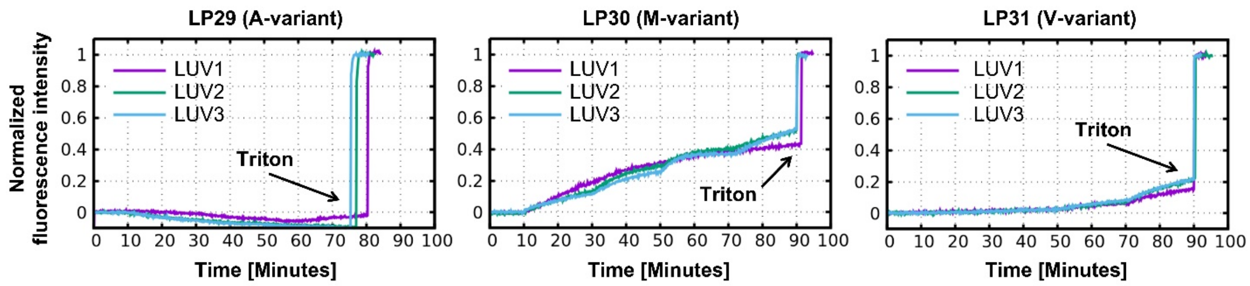

Time (Minutes)	Peptide:Lipid ratio (mol/mol)
10	1:100
30	1:50
50	1:20
70	1:10

Figure S7. ***In vitro*** **dye leakage activity.**

Peptide-induced leakage (efflux) of the self-quenched fluorescent dye calcein from the large unilamellar vesicles composed of POPC:POPG (1:1 mol/mol) lipids, shown as the normalized fluorescent intensity as a function of the investigated peptide:lipid ratios over the time. Surfactant triton was used as the control, causing 100% leakage in the end.

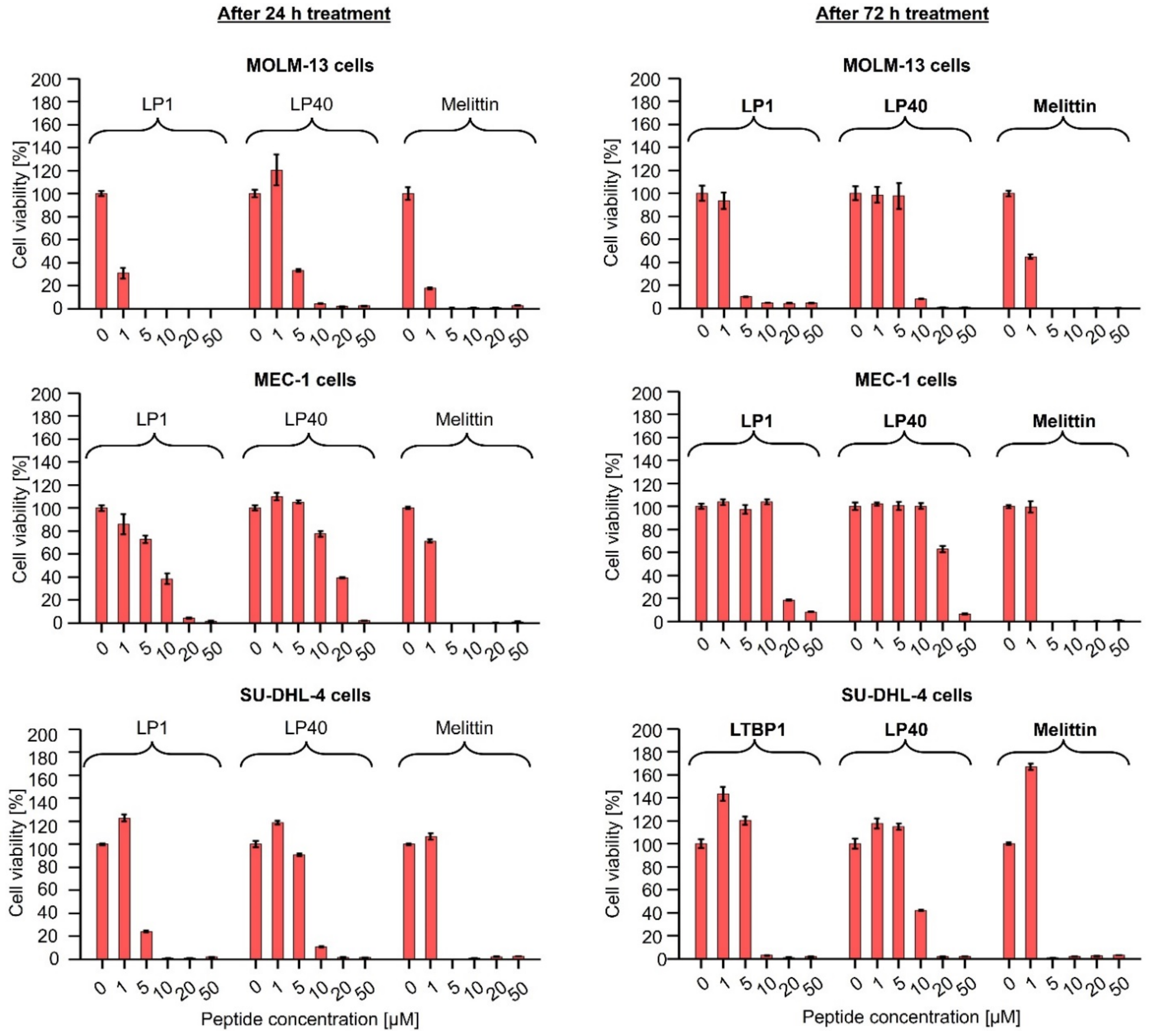

Figure S8. ***In vitro* anticancer activity.**
Activity of peptides against the leukemia and lymphoma cancer cell lines is reported as the percentage of cell viability (measured in quadruplicates) as a function of peptide concentration. Values are normalized to the control (set to 100%). Peptide sequences are provided in Table 1 in the main text.

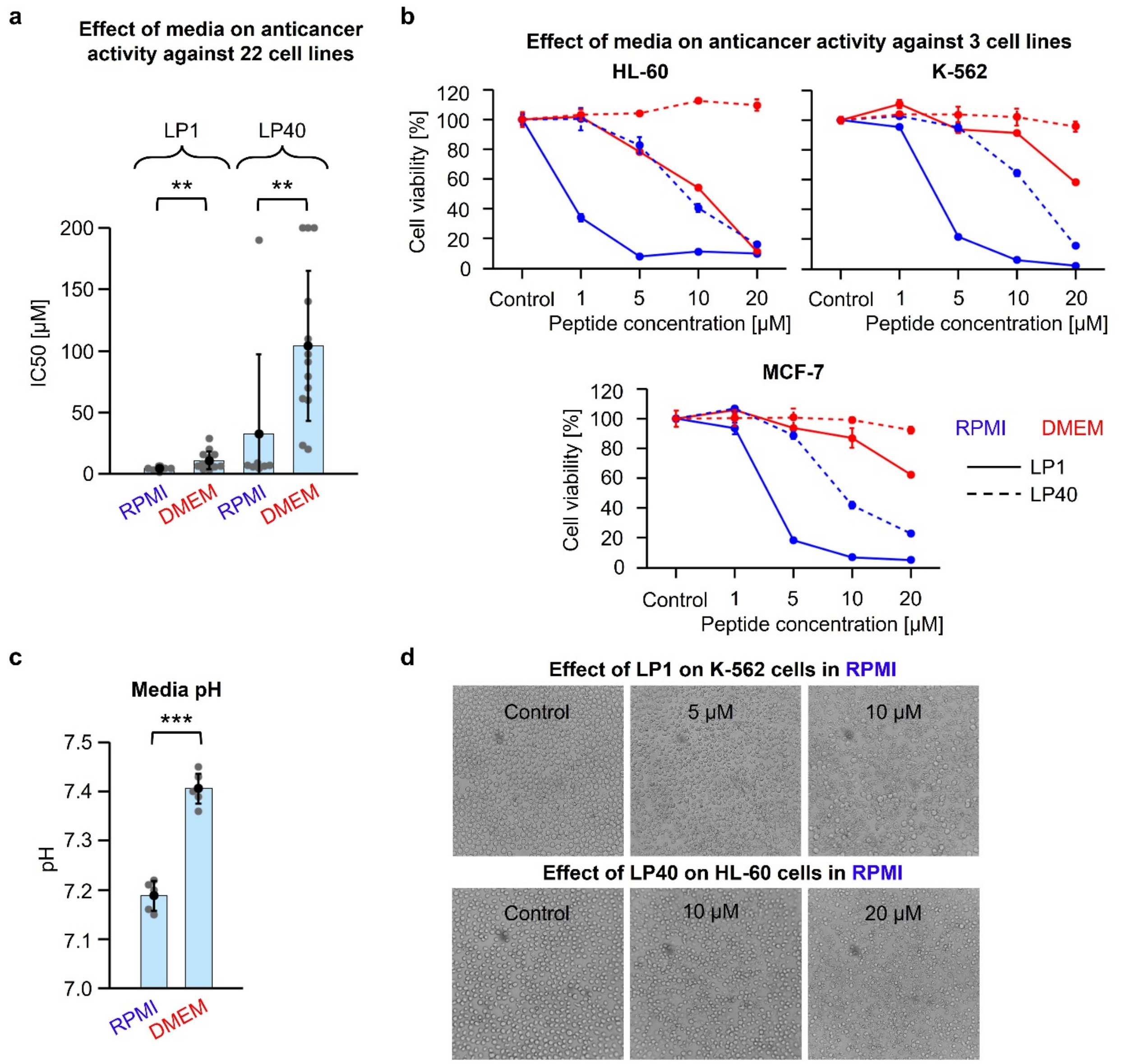

Figure S9. Effect of media on anticancer activity.

(a) Comparison of the activity (reported as the half maximal inhibitory concentration, IC50 value) of the peptides against the viability of 22 different human cell lines cultured in either RPMI or DMEM media for 72 hours (the main text Figure 6a). (b) Comparison of the activity (reported as the percentage cell viability measured in quadruplicates) of the peptides against three cancer cell lines, HL-60, K-562 and MCF-7, cultured in RPMI and DMEM media for 4 hours. (c) Comparison of pH between RPMI and DMEM medium, measured in five repetitions. (d) Images showing the rapid effect of the peptides against cancer cell lines after 30 (LP1) or 60 (LP40) minutes of treatment. PBS buffer was used as the control. Peptide sequences are provided in Table 1 in the main text. (a and c) The effect of media was compared using t-test or a two-way ANOVA followed by the Tukey post hoc test. $^{**}p < 0.01$ and $^{***}p < 0.001$.

a

pH	RPMI Control	RPMI LP1	RPMI LP40	DMEM Control	DMEM LP1	DMEM LP40	PBS Control	PBS LP1	PBS LP40
6 vs. 8.0	0.991	0.020	< 0.001	0.981	< 0.001	0.002	0.225	0.157	< 0.001
6 vs. 7.6	0.959	0.036	< 0.001	0.771	< 0.001	0.001	0.189	0.464	< 0.001
6 vs. 7.0	0.654	0.180	< 0.001	0.991	< 0.001	0.001	0.994	0.562	0.054

b

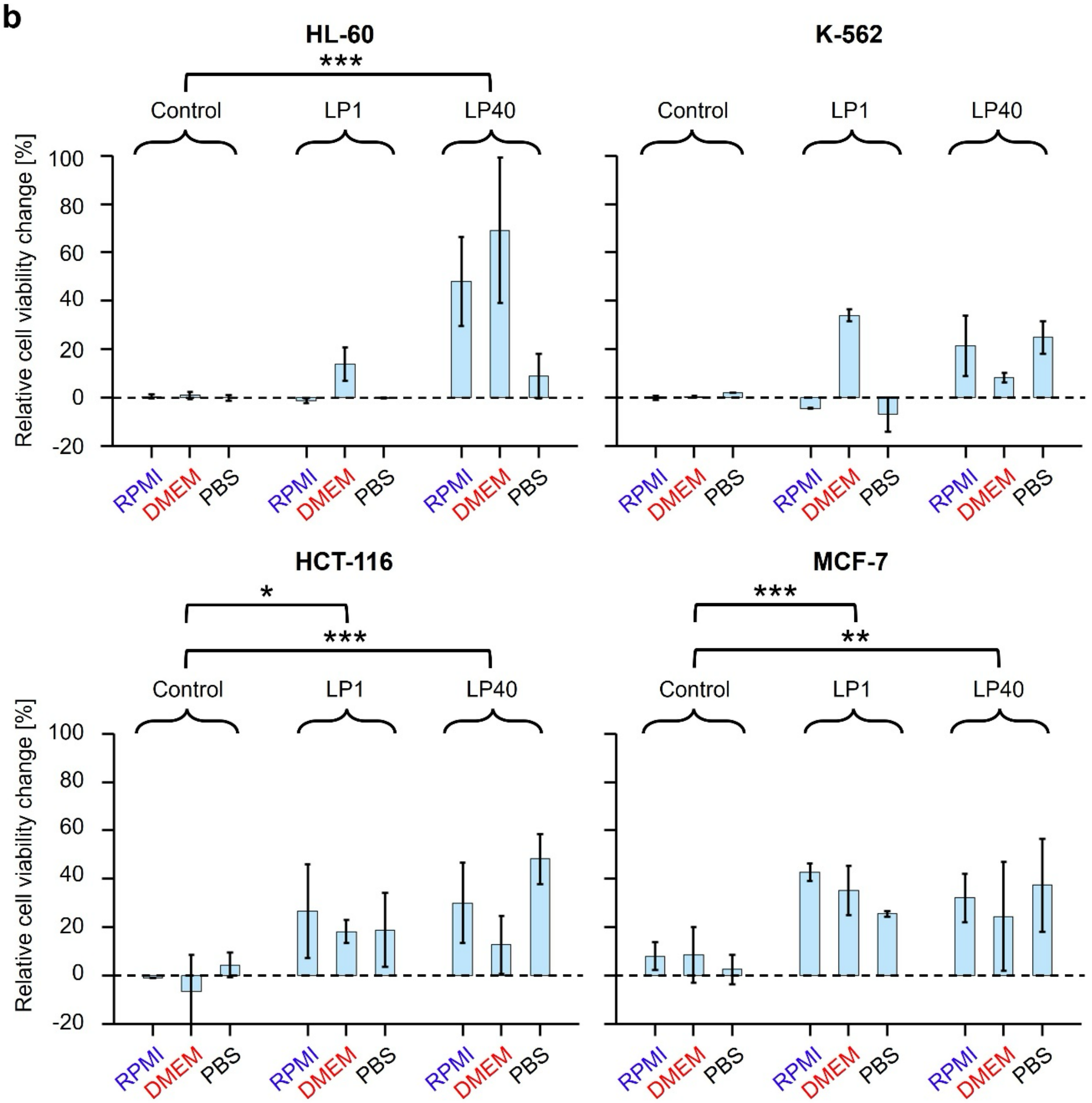

c

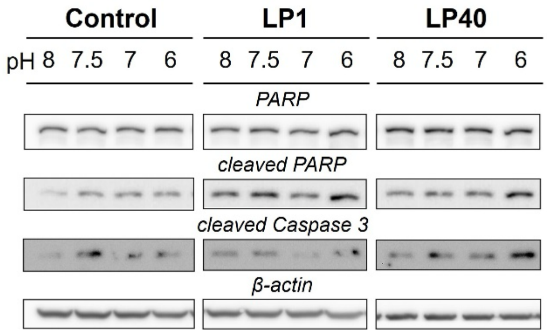

Figure S10. Effect of pH and media on anticancer activity.
(a) Statistical evaluation of peptide anticancer activity at 10 μM concentration for 1 hour against four different cancer cell lines cultured in three different media, RPMI, DMEM and PBS, adjusted to different pH values (measured in duplicates; the main text Figure 6c). The analysis was done using a three-way ANOVA followed by the Tukey post hoc test. The values indicate the p-value of statistical significance, which is marked in red when less than 0.05. (b) Relative changes in the percentage cell viability with the change in pH from 7 to 6 in HL-60, K-562, HCT-166, and MCF-7 cells. Values are calculated as viability at pH 7 minus viability at pH 6. PBS buffer was used as the control. The overall effect of peptides across the cell lines was compared using four-way ANOVA followed by Tukey post hoc test which showed LP40 activity as more dependent on pH compared to LP1 ($p = 0.002$). $*p < 0.05$; $**p < 0.01$; $***p < 0.001$; not indicated = not significant. (c) Western blot showing that the ongoing apoptosis of MCF-7 cells treated with LP1 (5 μM) and LP40 (10 μM) peptides is dependent on the pH of the DMEM medium. Peptide sequences are provided in Table 1 in the main text.

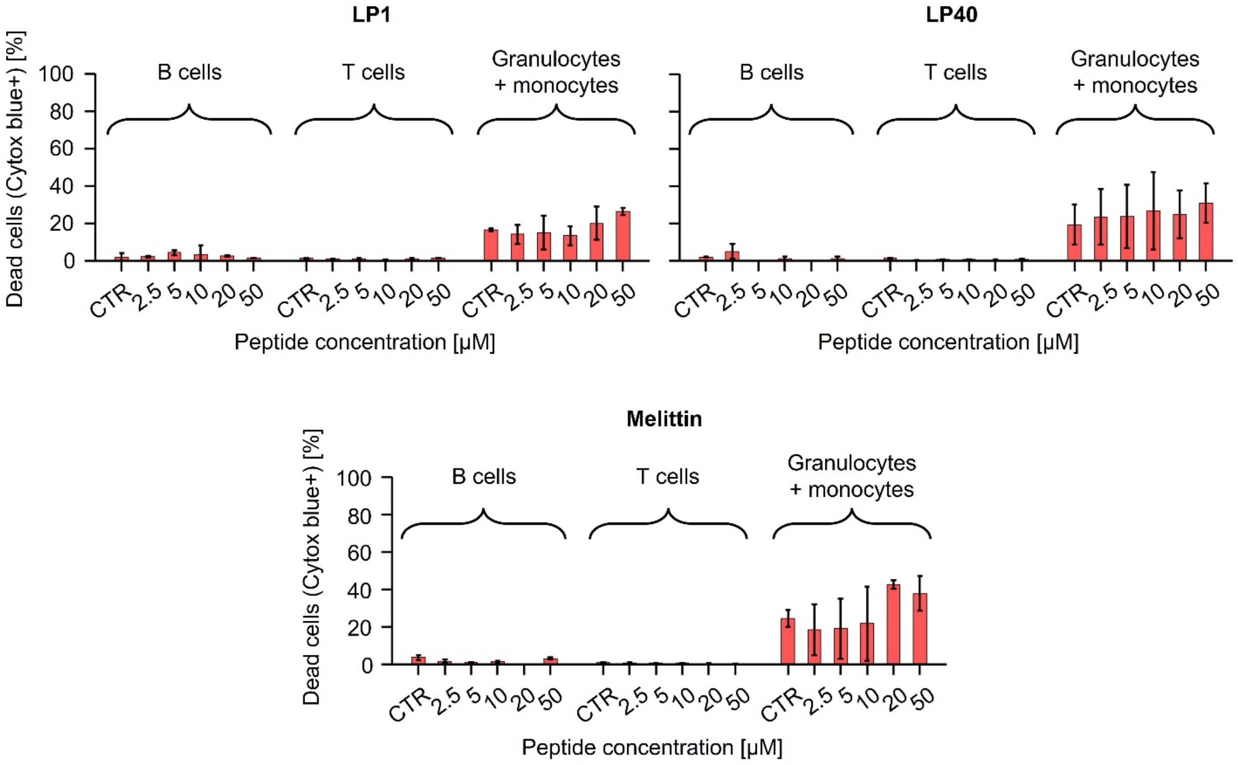

Figure S11. Flow cytometric analysis of whole blood.
The effect of peptides on the healthy primary cells in whole blood from a healthy donor was measured in duplicates. Peptide melittin and PBS buffer were used as controls.

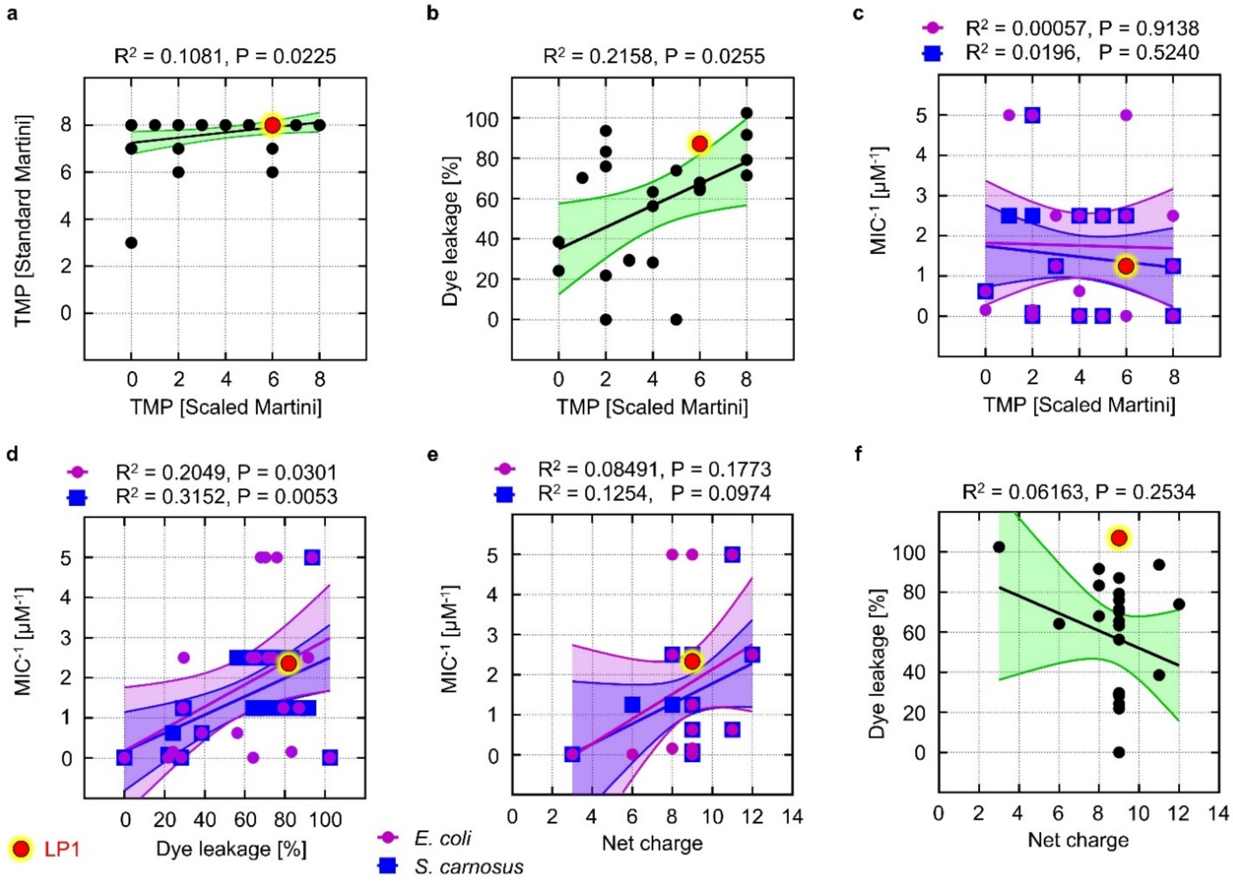

Figure S12. Correlations between pore stability, *in vitro* activity, and peptide net charge. Linear regression analysis was performed - **(a)** between the number of transmembrane peptides after 51 µs simulation run (denoted as TMP) using the standard and 'scaled' Martini force fields; **(b-c)** between the TMP using the 'scaled' Martini force field and (b) dye leakage and (c) antimicrobial activity; **(d)** between dye leakage and antimicrobial activity; and **(e-f)** between peptide net charge and (e) antimicrobial activity and (f) dye leakage. GraphPad Prism software was used for analysis and 95% confidence intervals are shown. Note that TMP indicates the stability of transmembrane barrel pores (TBPs). The pore was considered a 'stable' TBP if it consisted of at least six TMPs, i.e., hexamer; in the case of pentamer and tetramer, the pore was considered 'deformed'; and finally, if there were less than four TMPs, the pore was considered 'closed'. The percentage leakage of the fluorescent dye calcein at a peptide-to-lipid molecular ratio of 1:100 from the POPC:POPG (1:1 mol/mol) large unilamellar vesicles (see Table 2 in the main text) was used for analysis. Antimicrobial activity was used as the inverse of the MIC values (see Table 2 in the main text) for the analysis.

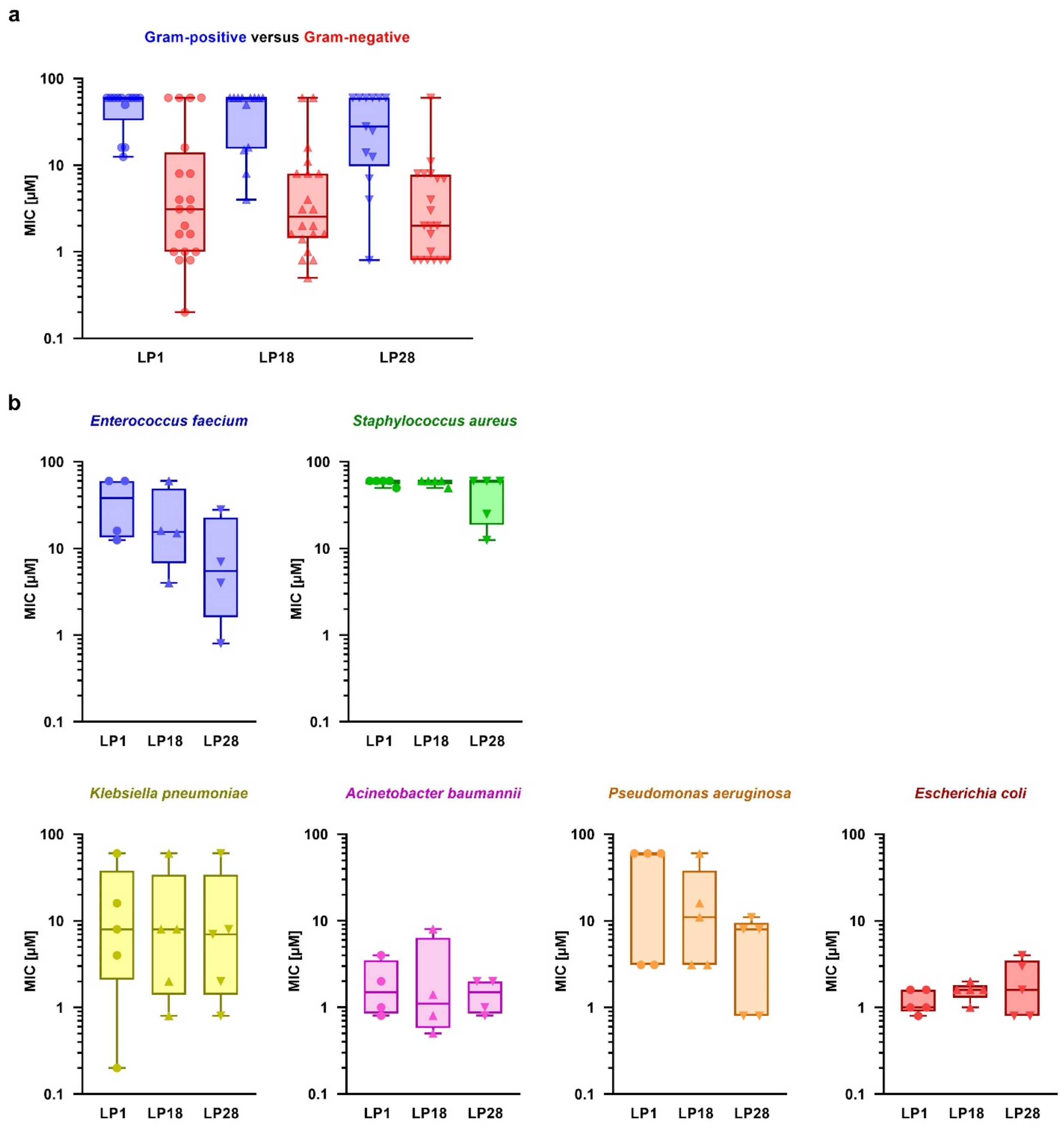

Figure S13. Comparison of antimicrobial activity against Gram-positive and Gram-negative bacteria. Box-whisker plots, showing the median, upper quartile, lower quartile, and whiskers from minimum to maximum, indicate the difference in antimicrobial activity of three peptides, LP1, LP18 and LP28, against Gram-positive and Gram-negative bacteria. The comparison was made considering all MIC values against a panel of 33 ESKAPEE pathogens tested in this study (see Figure 3a-d in the main text) using GraphPad Prism software. Note that the MIC value of the inactive cases, i.e., where the peptides remained inactive up to the highest concentrations tested, was uniformly considered to be 60 μM (the highest concentration tested in Figure 3a-d in the main text). Peptide sequences are given in Table 1 in the main text.

Supplementary Materials

Peptide LP1 = **KKKK**L**KK**IL**KK**L**F**Q**F**INQL**D**NQLQ**D**I**K**QN**K**-NH2

HPLC Certificate

**HPLC Column**	(250×4.6mm I.D.) C18
**Detection wavelength**	220 nm
**Gradient**	20-50%B in 30 min
**Buffer A**	0.05%TFA +2%CH_3CN
**Buffer B**	0.05%TFA +90%CH_3CN

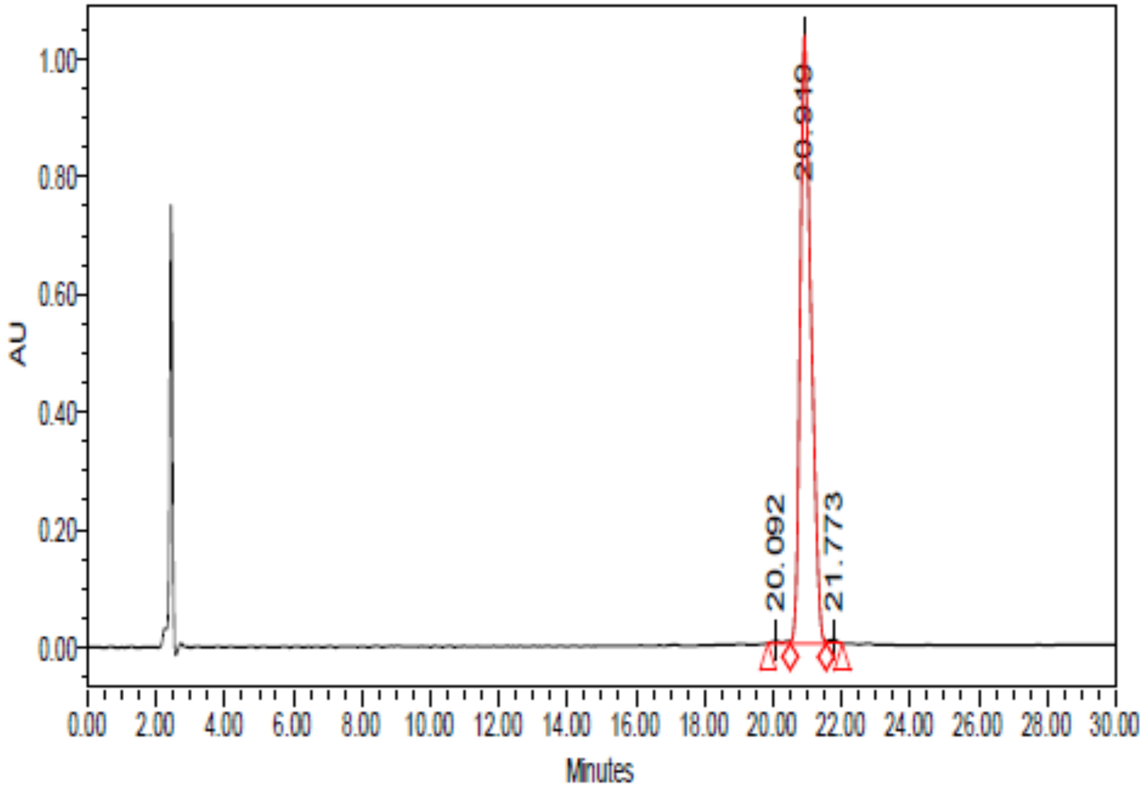

	Retention Time	Area	% Area	Height
1	20.092	106399	0.46	4330
2	20.919	22791833	98.97	1032662
3	21.773	131817	0.57	7096

Mass Spectrometry Certificate

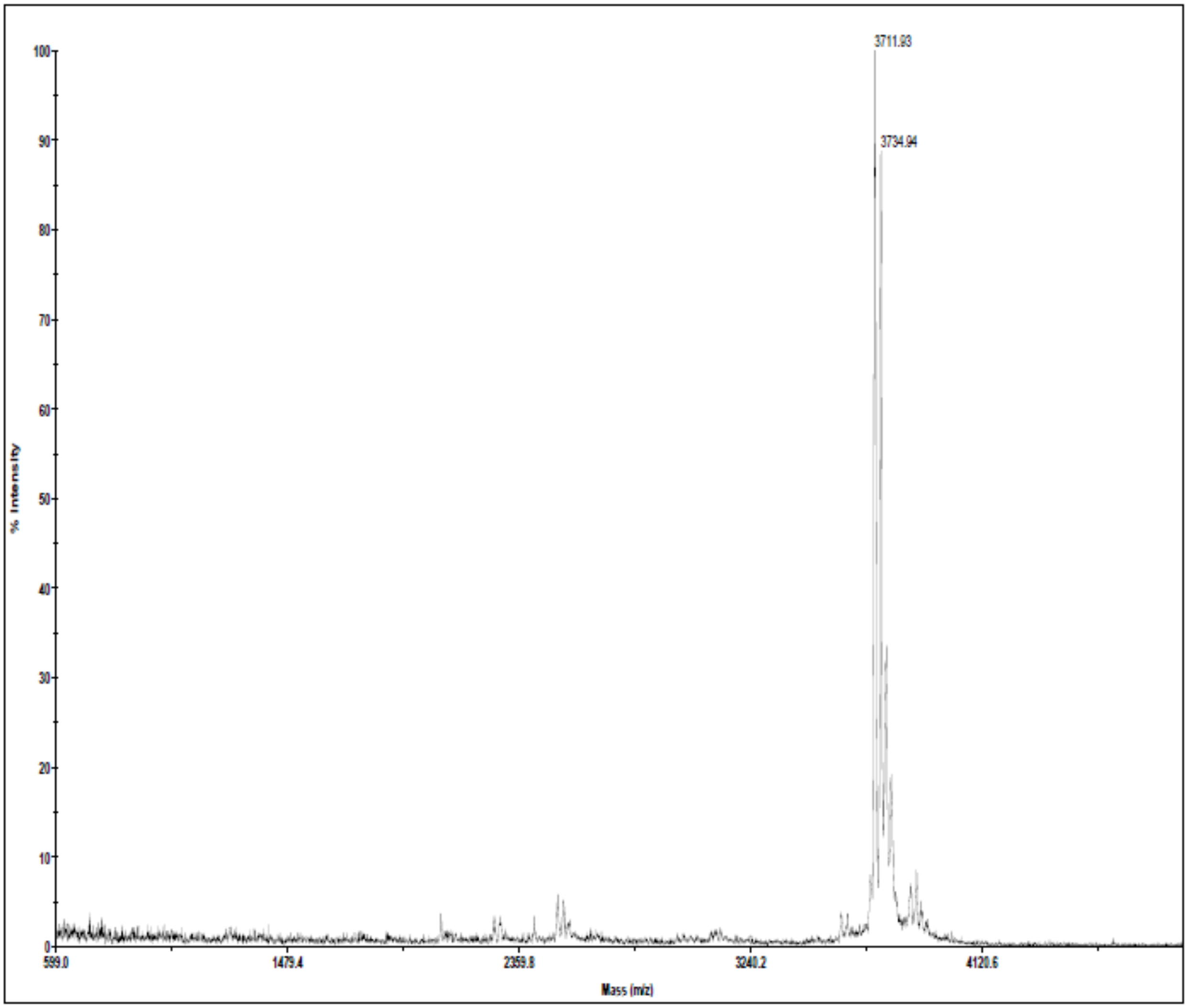

Lot No.: P111019-01-01
Method: MALDI-TOF
Main Peak: 3711.93
MW [M+H$^+$]: 3711.93
MW: 3710.93
Theoretical MW: 3711.58
Match: Approved
Z=1

Peptide LP2 = KKKKLKKILKKLIQLINQLDNQLQDIKQNK-NH2

HPLC Certificate

HPLC Column	(250×4.6mm I.D.) C18
Detection wavelength	220 nm
Gradient	20-50%B in 30 min
Buffer A	0.05%TFA +2%CH_3CN
Buffer B	0.05%TFA +90%CH_3CN

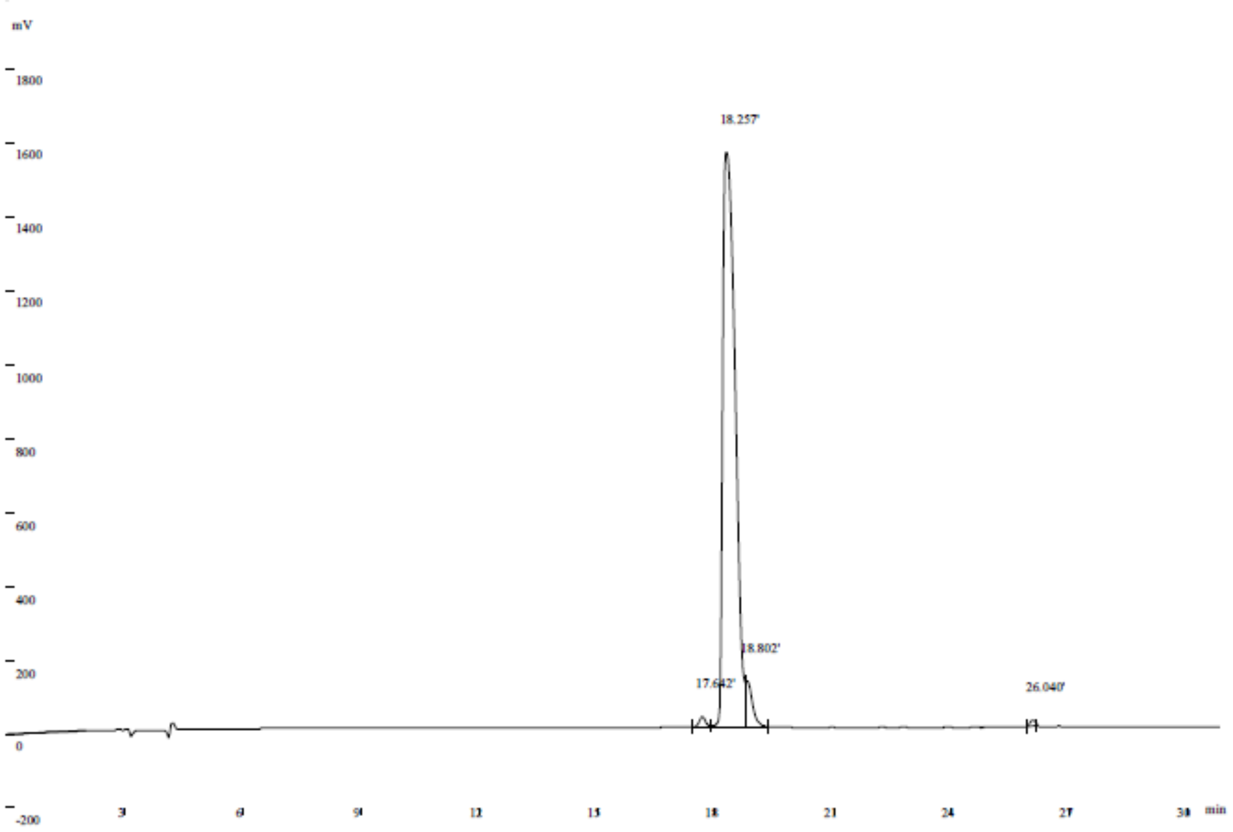

Peak Results

Rank	Time	Quantity	Area	Height
1	17.642	0.788	261152	26323
2	18.257	95.23	31564531	1553152
3	18.802	3.636	1205236	119613
4	26.040	0.3421	113367	14184
Total		100	33144286	1713272

Mass Spectrometry Certificate

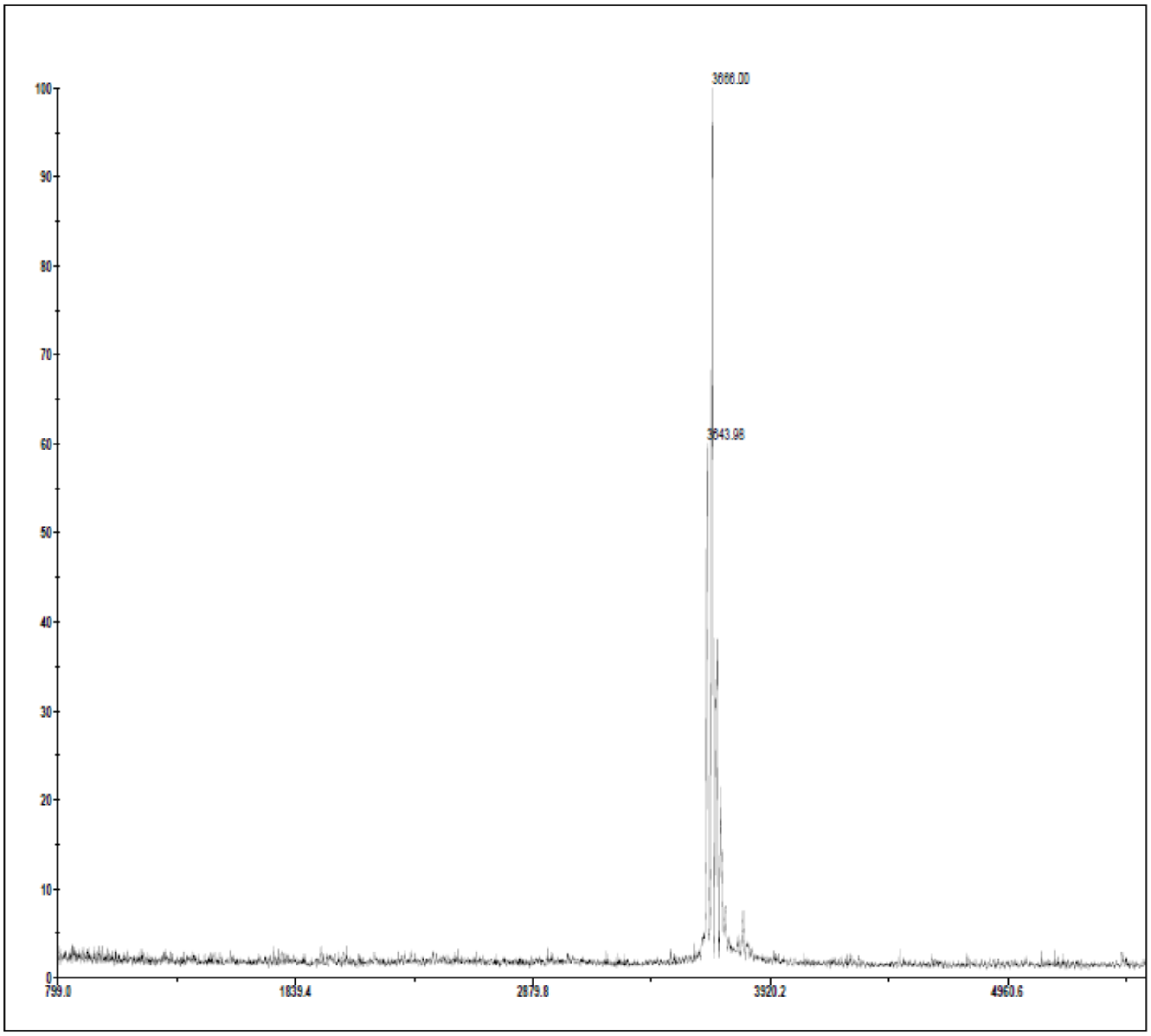

Lot No.: P061021-02-06
Method: MALDI-TOF
Main Peak: 3666.00
MW [M+Na$^+$]: 3666.00
MW: 3643.00
Theoretical MW: 3643.54
Match: Approved
Z=1

Peptide LP3 = KKKKLKKILKKLWQWINQLDNQLQDIKQNK-NH2

HPLC Certificate

**HPLC Column**	(250×4.6mm I.D.) C18
**Detection wavelength**	220 nm
**Gradient**	30-60%B in 30 min
**Buffer A**	0.05%TFA +2%CH_3CN
**Buffer B**	0.05%TFA +90%CH_3CN

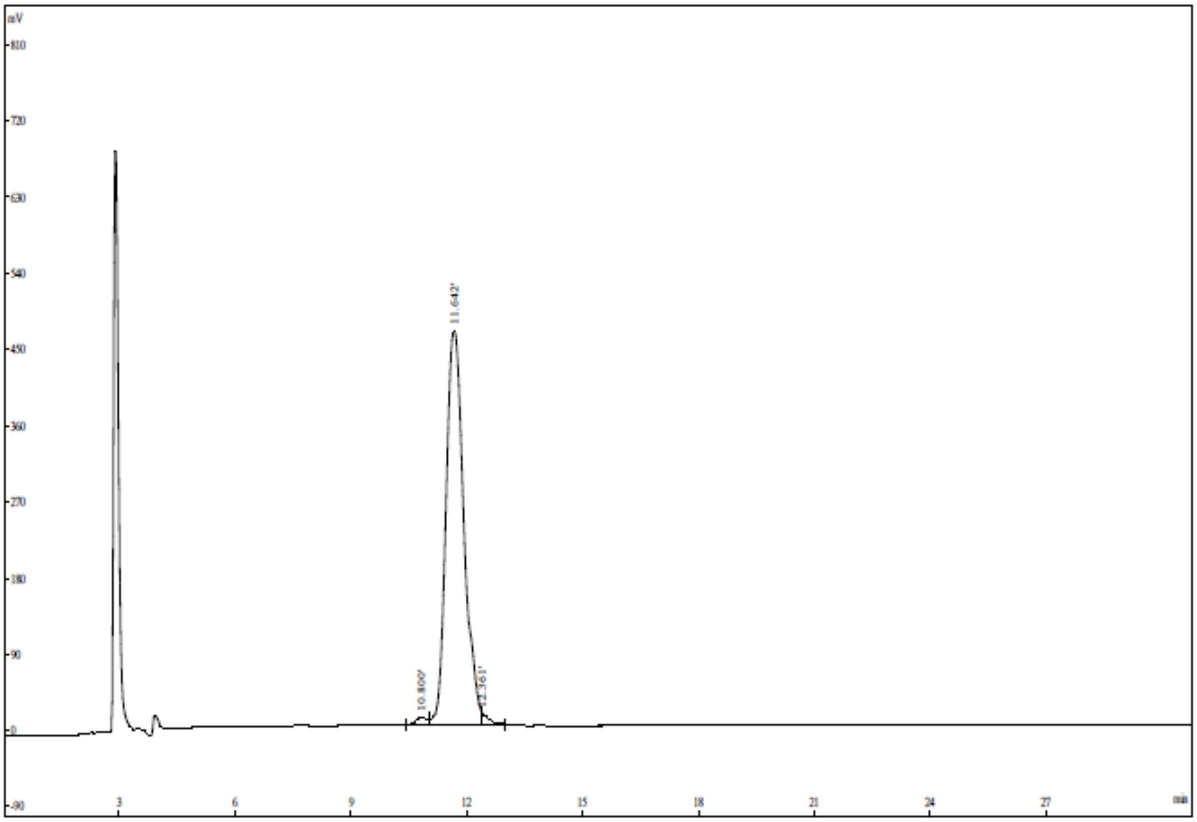

Peak Results

Rank	Time	Quantity	Area	Height
1	10.800	1.187	173204	8539
2	11.642	97.73	14257872	463926
3	12.361	1.078	157240	13178
Total		100	14588316	485643

Mass Spectrometry Certificate

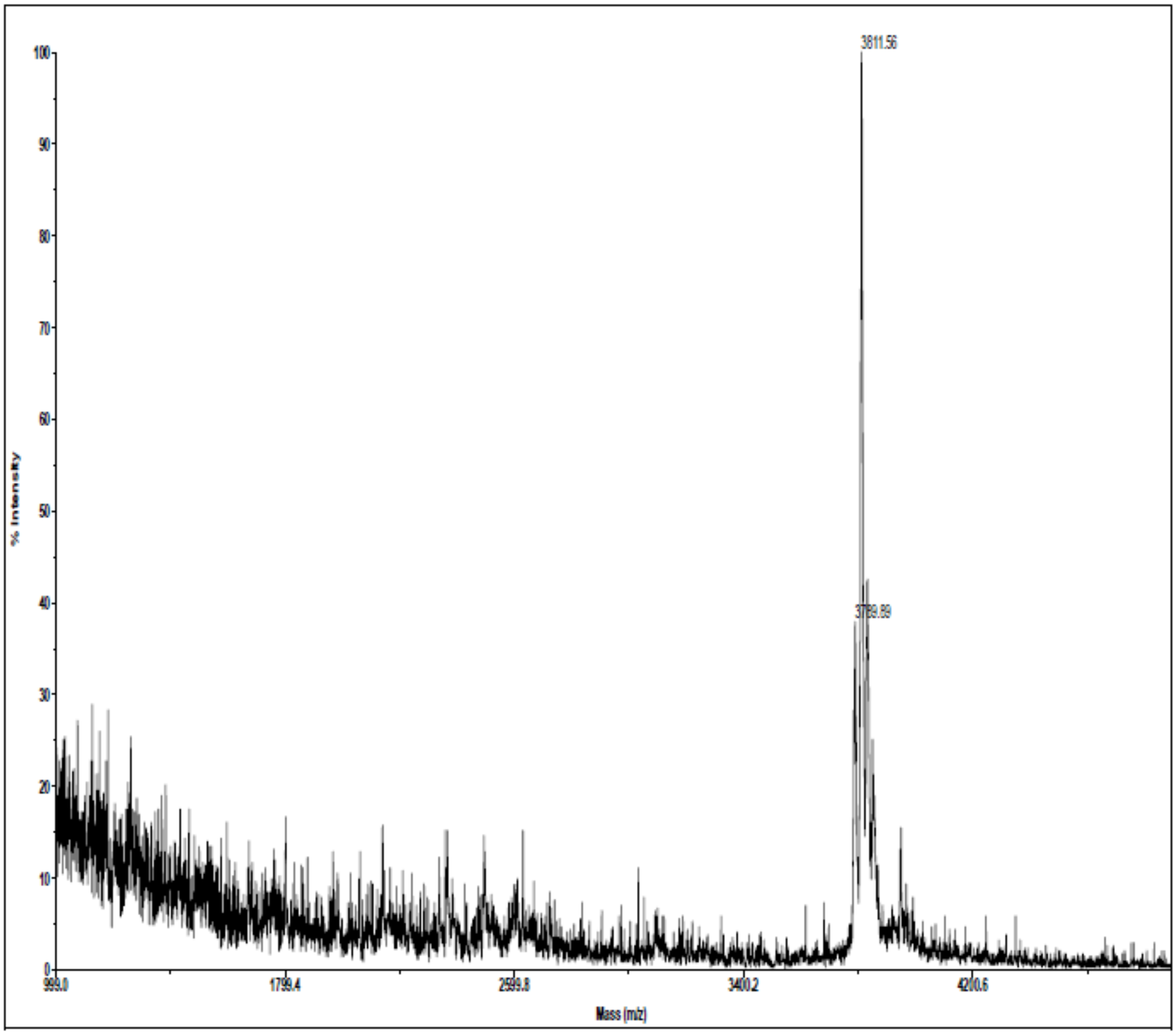

Lot No.: P300720-01-01
Method: MALDI-TOF
Main Peak: 3811.56
MW [M+Na$^+$]: 3811.56
MW: 3788.56
Theoretical MW: 3789.65
Match: Approved
Z=1

Peptide LP8 = KKKKLKKILKKLFQFINQLENQLQEIKQNK-NH2

HPLC Certificate

**HPLC Column**	(250×4.6mm I.D.) C18
**Detection wavelength**	220 nm
**Gradient**	30-60%B in 30 min
**Buffer A**	0.05%TFA +2%CH_3CN
**Buffer B**	0.05%TFA +90%CH_3CN

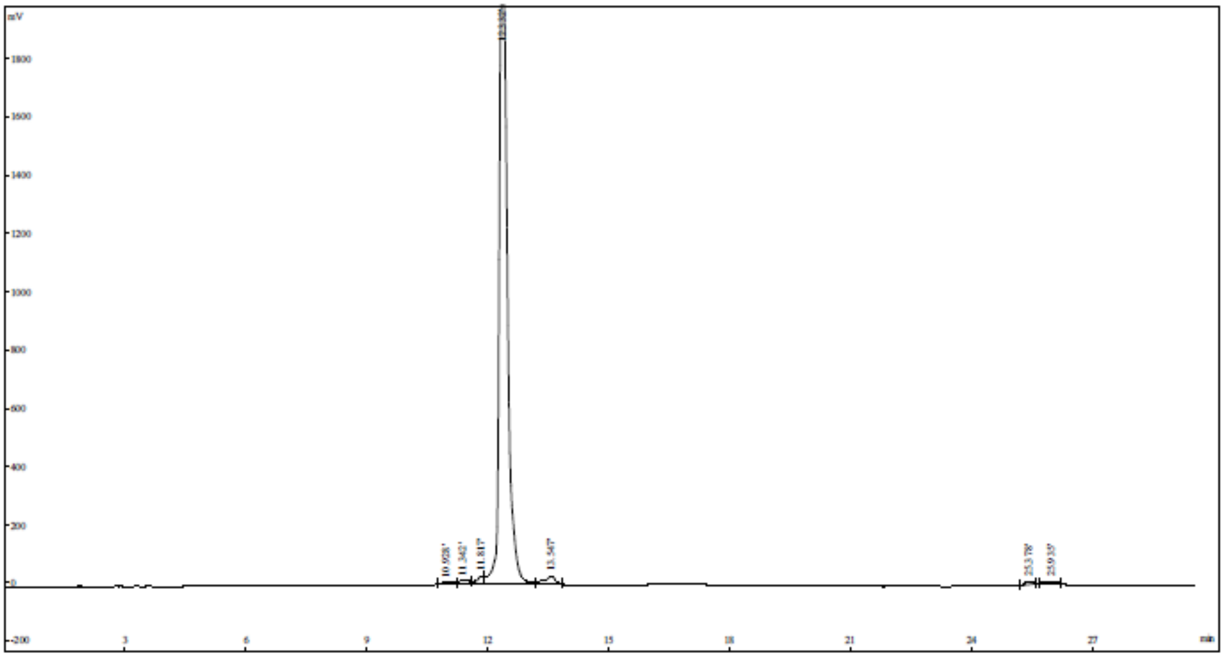

Peak Results

Rank	Time	Quantity	Area	Height
1	10.928	0.3776	106910	3671
2	11.342	0.6806	192716	11808
3	11.817	1.138	322298	28013
4	12.332	95.52	27047110	2003854
5	13.547	1.599	452735	28526
6	25.378	0.2992	84724	7332
7	25.935	0.3831	108463	6016
Total		100	28314956	2089220

<u>**Mass Spectrometry Certificate**</u>

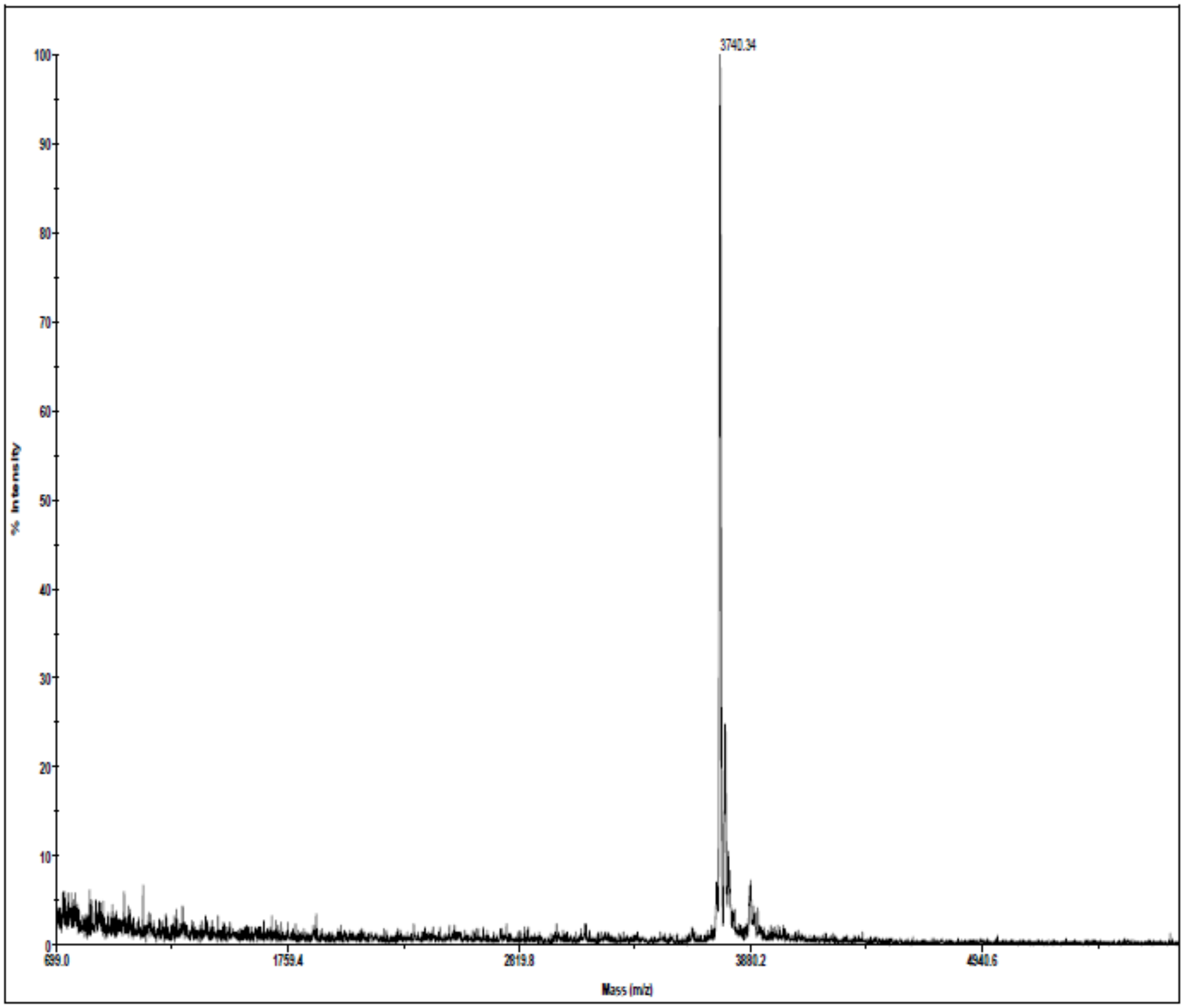

Lot No.: P300720-01-08
Method: MALDI-TOF
Main Peak: 3740.34
MW [M+H^{+}]: 3740.34
MW: 3739.34
Theoretical MW: 3739.63
Match: Approved
Z=1

Peptide LP18 = KKKKLKKILKKLFQFINQLDNQLQDIKKKK-NH2

HPLC Certificate

**HPLC Column**	(250×4.6mm I.D.) C18
**Detection wavelength**	220 nm
**Gradient**	30-60%B in 30 min
**Buffer A**	0.05%TFA +2%CH_3CN
**Buffer B**	0.05%TFA +90%CH_3CN

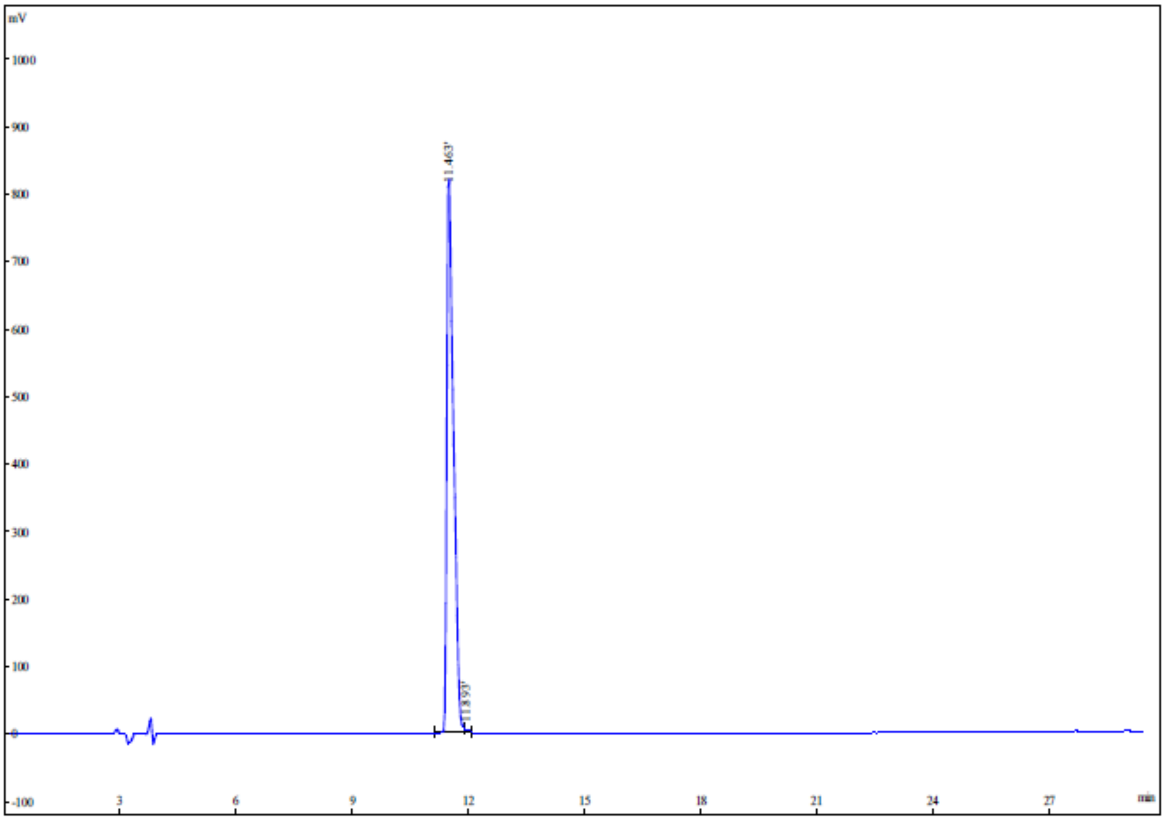

Peak Results

Rank	Time	Conc.	Area	Height
1	11.463	99.72	9898025	844758
2	11.893	0.281	27888	4755
Total		100	9925913	849513

Mass Spectrometry Certificate

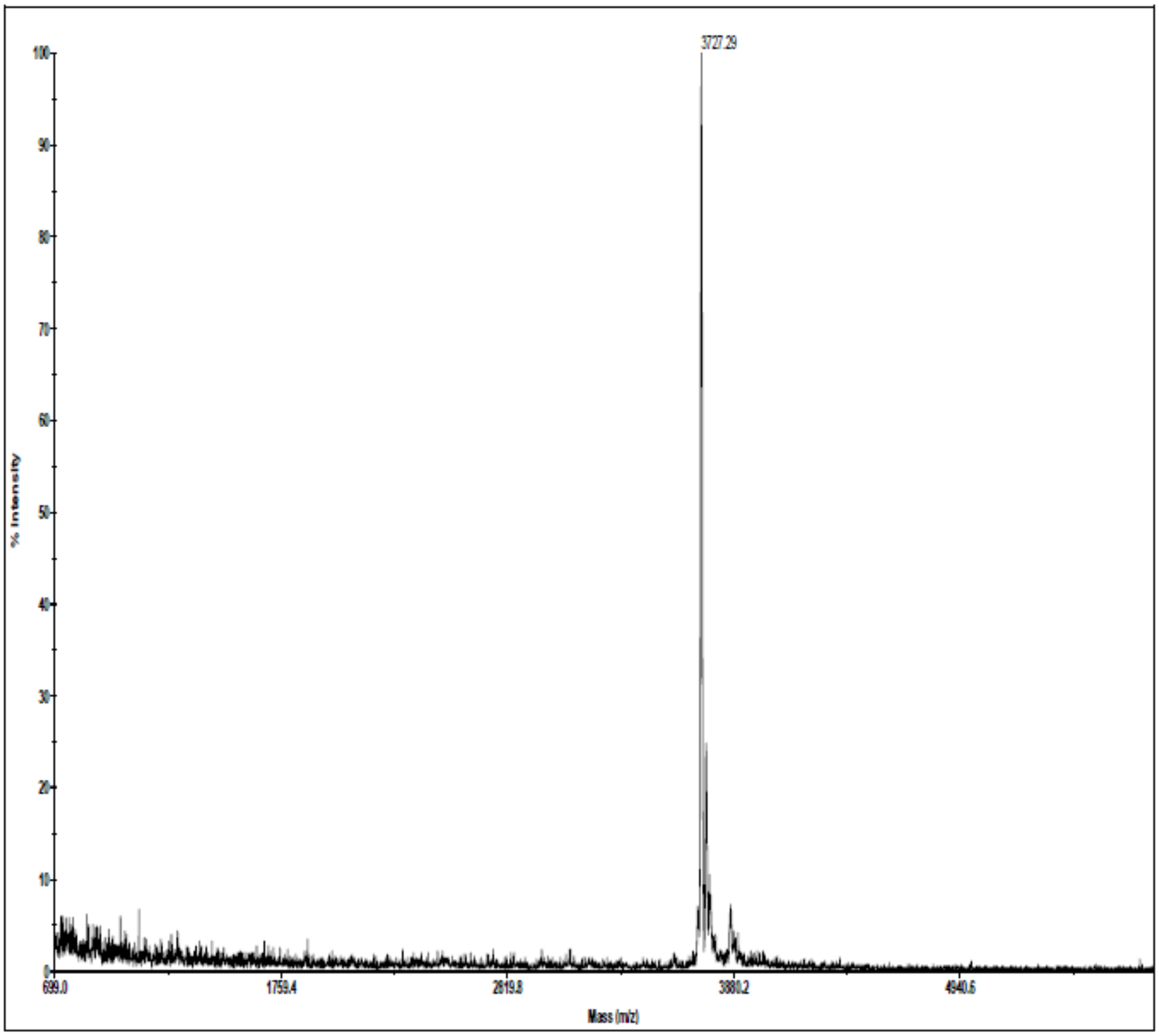

Lot No.: P300720-01-04
Method: MALDI-TOF
Main Peak: 3727.29
MW [M+H$^+$]: 3727.29
MW: 3726.29
Theoretical MW: 3725.69
Match: Approved
Z=1

Peptide LP20 = KKKKLKKILKKLFQFIDQLINDLQNIKKKK-NH2

HPLC Certificate

**HPLC Column**	(250×4.6mm I.D.) C18
**Detection wavelength**	220 nm
**Gradient**	28-58%B in 30 min
**Buffer A**	0.05%TFA +2%CH_3CN
**Buffer B**	0.05%TFA +90%CH_3CN

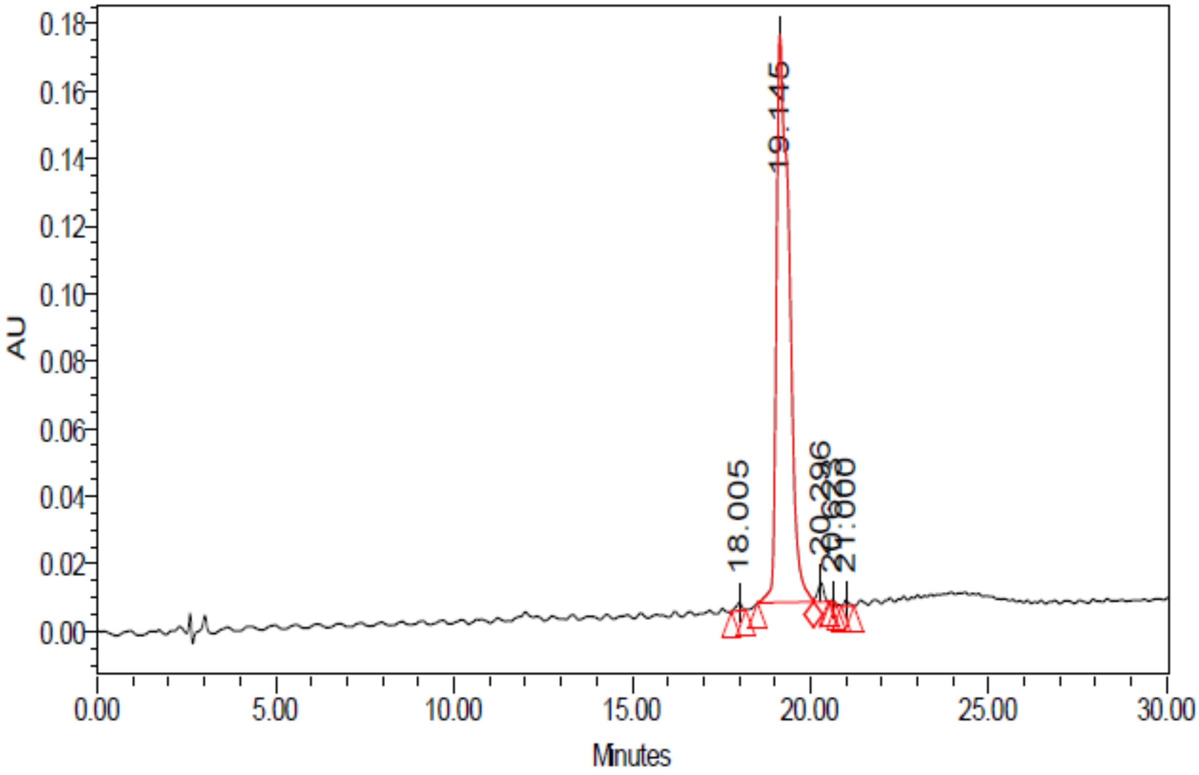

	Retention Time	Area	% Area	Height
1	18.005	25976	0.61	2365
2	19.145	4181772	97.43	168064
3	20.296	64828	1.51	5563
4	20.623	4426	0.10	740
5	21.000	14874	0.35	1755

Mass Spectrometry Certificate

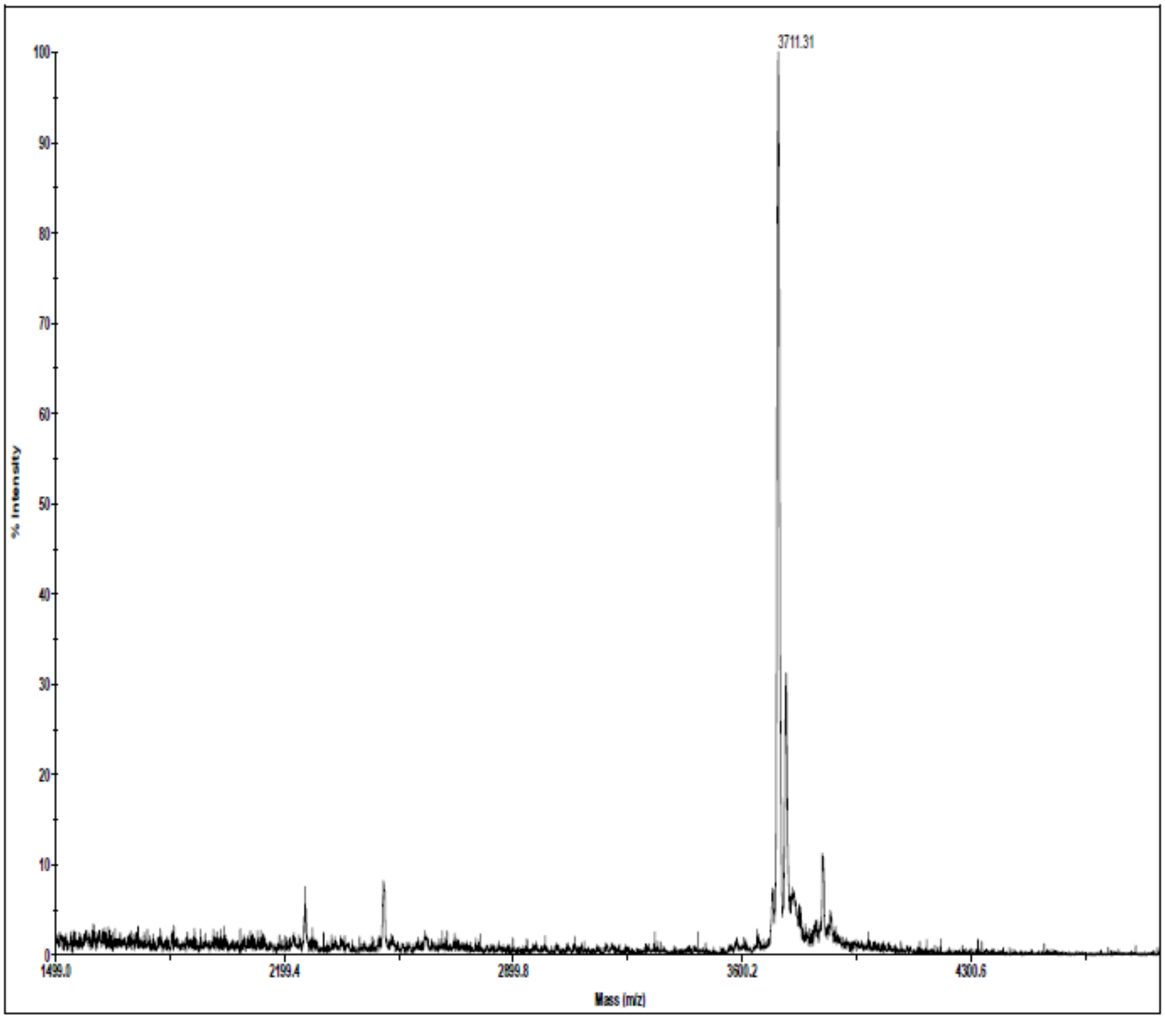

Lot No.: P300720-01-05
Method: MALDI-TOF
Main Peak: 3711.31
MW [M+H$^+$]: 3711.31
MW: 3710.31
Theoretical MW: 3710.72
Match: Approved
Z=1

Peptide LP22 = KKKKLKKILKKLFQFINQLDNQLQDIKQNK

HPLC Certificate

**HPLC Column**	(250×4.6mm I.D.) C18
**Detection wavelength**	220 nm
**Gradient**	15-45%B in 30 min
**Buffer A**	0.05%TFA +2%CH_3CN
**Buffer B**	0.05%TFA +90%CH_3CN

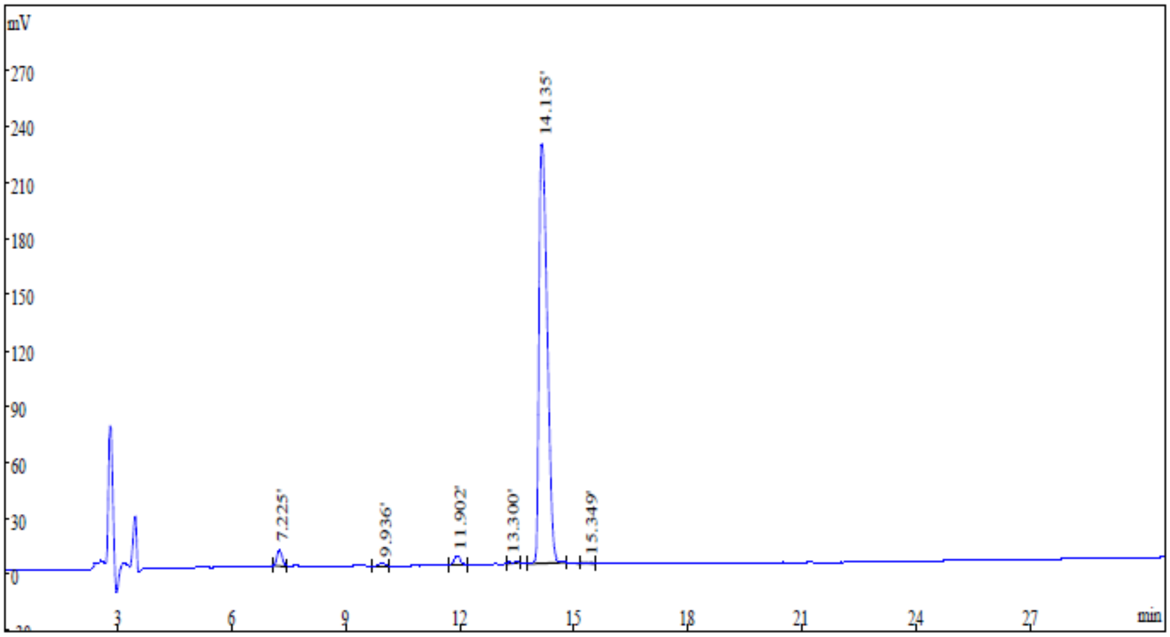

Peak Results

Rank	Time	Conc.	Area	Height
1	7.225	2.139	67938	8225
2	9.936	0.3553	11285	1279
3	11.902	1.716	54493	4952
4	13.300	0.5271	16742	1240
5	14.135	95.06	3019533	223829
6	15.349	0.1988	6313	508
Total		100	3176304	240033

Mass Spectrometry Certificate

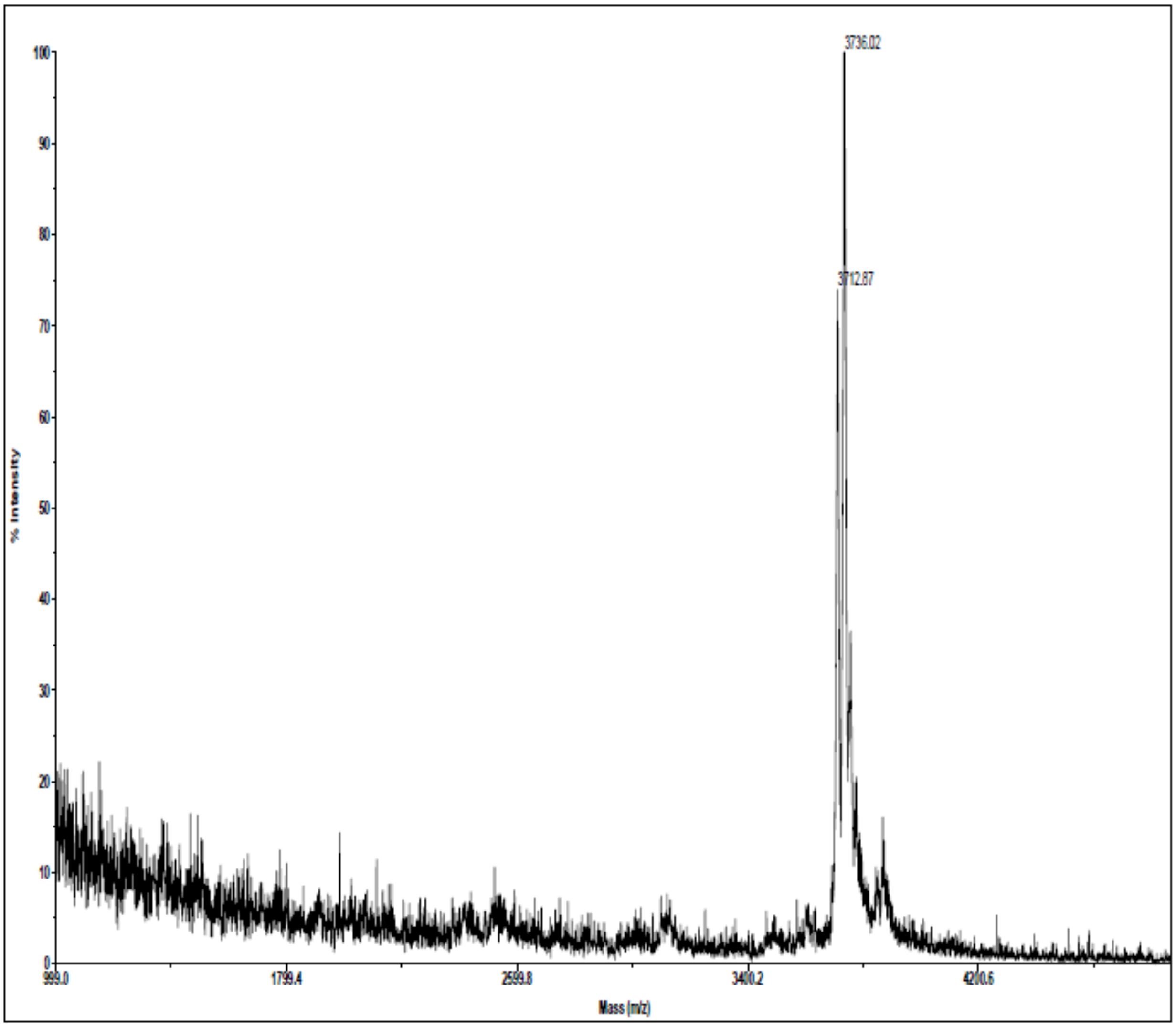

Lot No.: P300720-01-02
Method: MALDI-TOF
Main Peak: 3736.02
MW [M+ Na^+]: 3736.02
MW: 3713.02
Theoretical MW: 3712.56
Match: Approved
Z=1

Peptide LP23 = Ac-**KKKK**L**KK**IL**KK**L**F**Q**F**INQL**D**NQLQ**D**I**K**QN**K**-NH2

HPLC Certificate

HPLC Column	(250×4.6mm I.D.) C18
Detection wavelength	220 nm
Gradient	32-62%B in 30 min
Buffer A	0.05%TFA +2%CH_3CN
Buffer B	0.05%TFA +90%CH_3CN

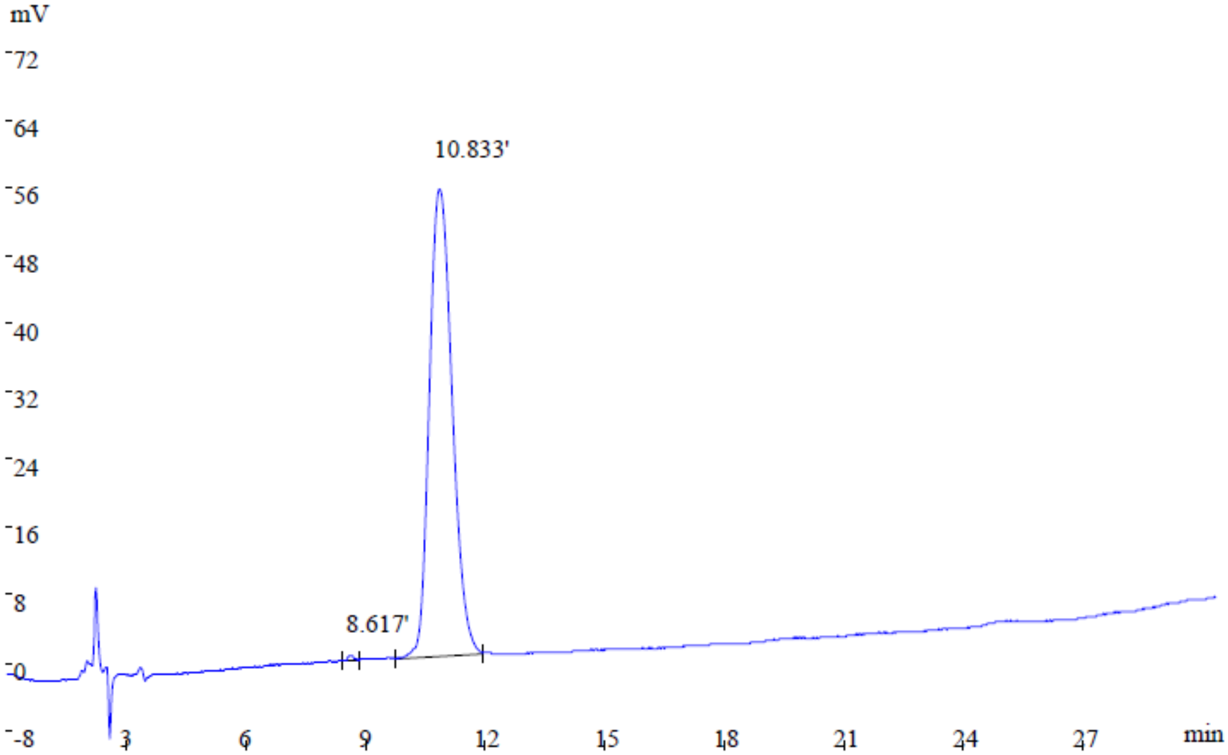

Peak Results

Rank	Time	Conc.	Area	Height
1	8.617	0.2548	5400	707
2	10.833	99.75	2113573	54859
Total		100	2118973	55566

Mass Spectrometry Certificate

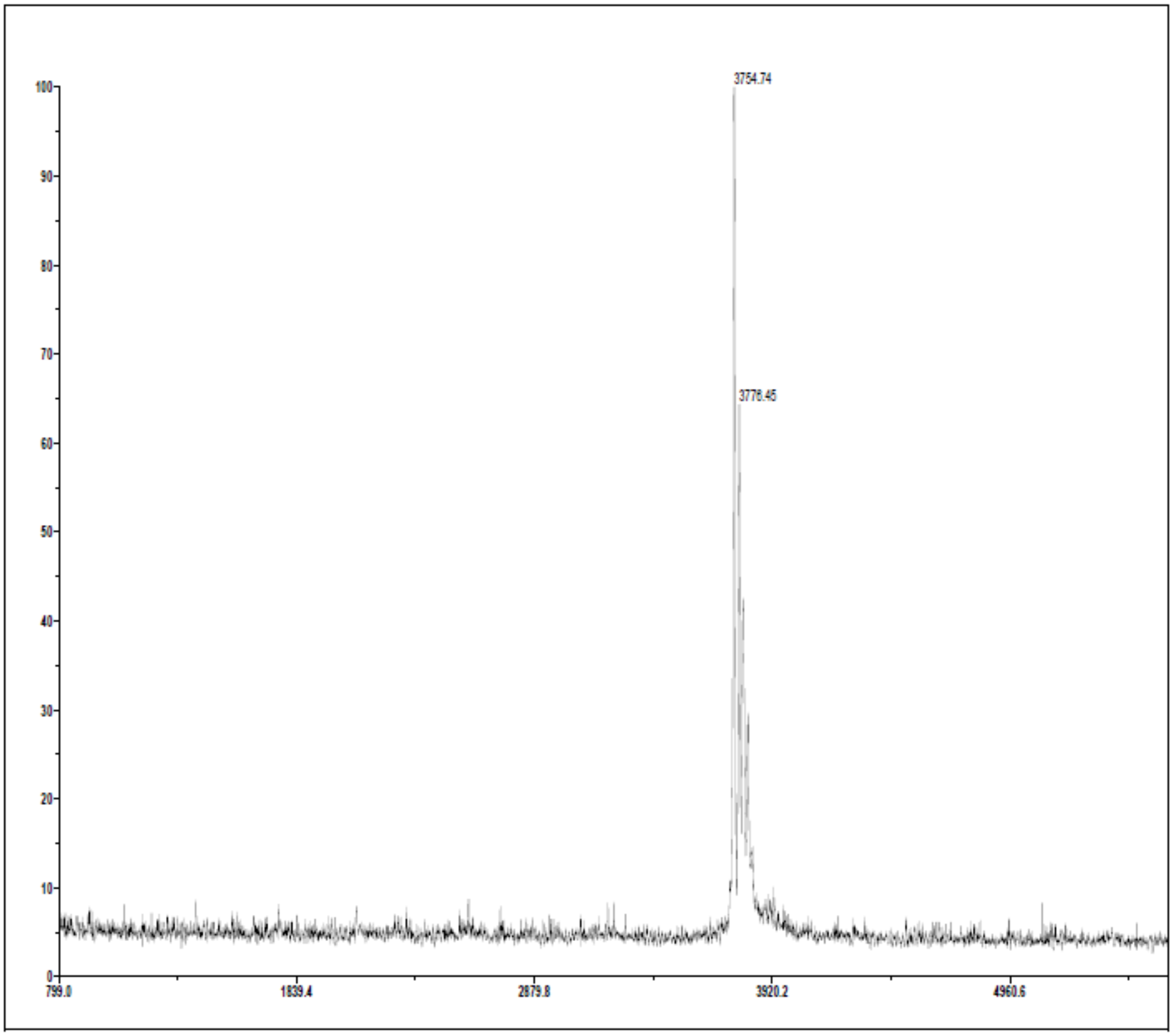

Lot No.: P061021-02-05
Method: MALDI-TOF
Main Peak: 3754.74
MW [M+H$^+$]: 3754.74
MW: 3753.74
Theoretical MW: 3753.61
Match: Approved
Z=1

Peptide LP26 = KKKKLKKILKKLFQFINQLDNQLQDFKQNK

HPLC Certificate

HPLC Column	(250×4.6mm I.D.) C18
Detection wavelength	220 nm
Gradient	15-45%B in 30 min
Buffer A	0.05%TFA +2%CH_3CN
Buffer B	0.05%TFA +90%CH_3CN

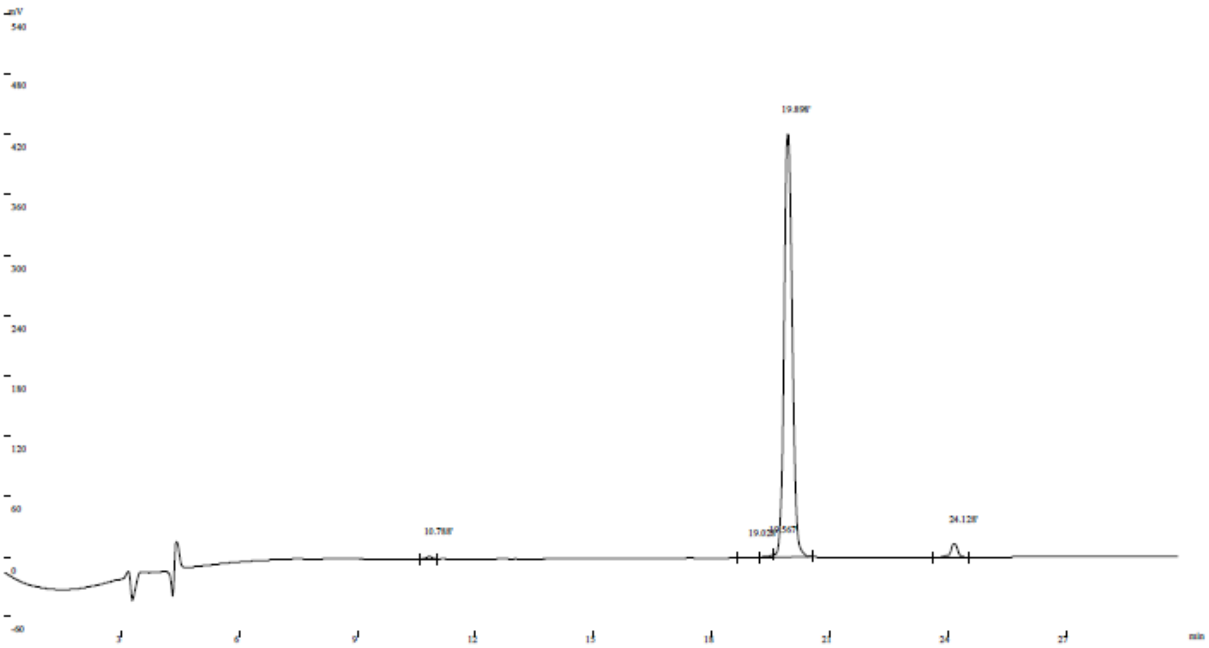

Peak Results

Rank	Time	Quantity	Area	Height
1	10.788	0.3634	20656	2173
2	19.028	0.04833	2747	392
3	19.567	0.4885	27764	3042
4	19.898	96.63	5491806	420109
5	24.128	2.474	140617	13378
Total		100	5683590	439094

Mass Spectrometry Certificate

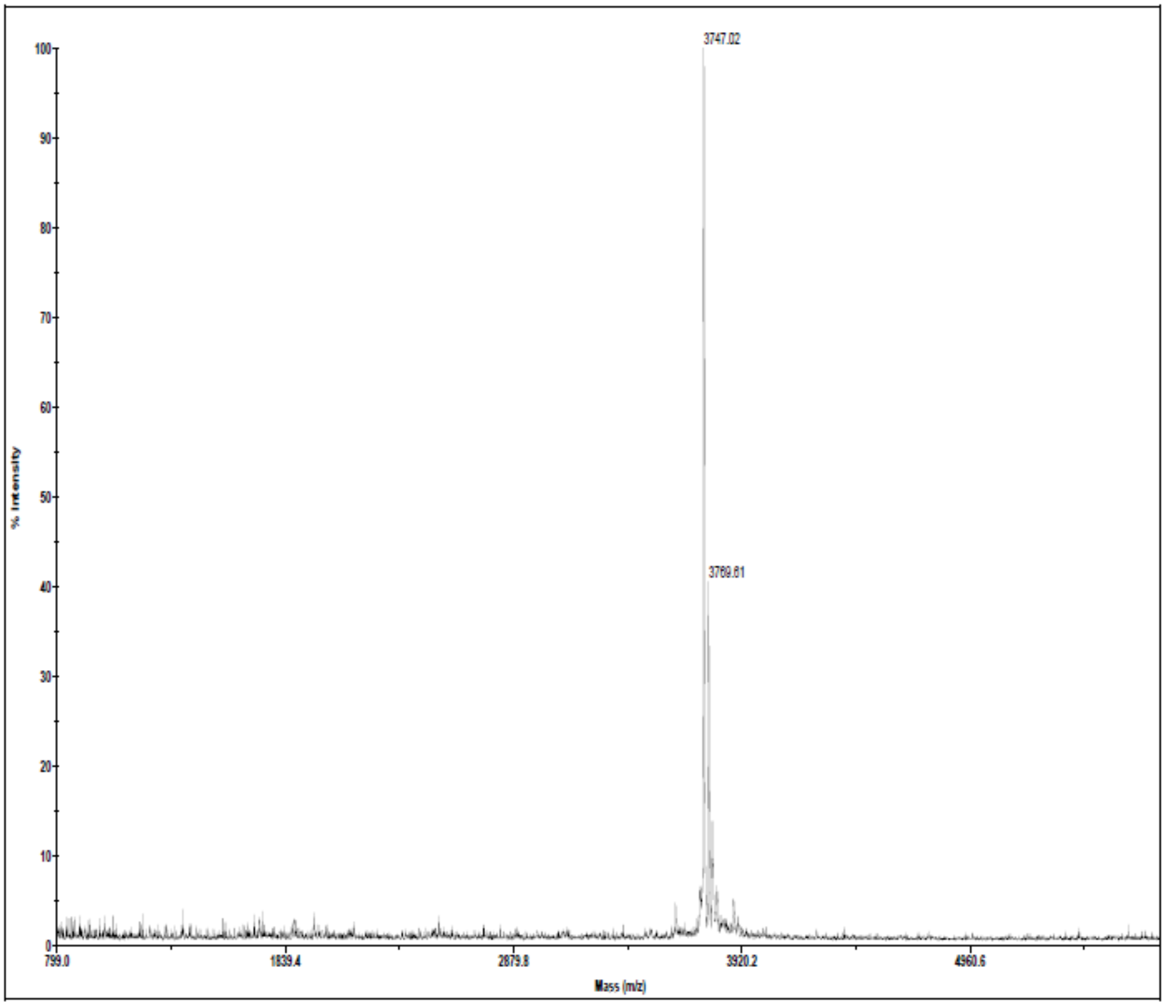

Lot No.: P071220-01-11
Method: MALDI-TOF
Main Peak: 3747.02
MW [M+H$^+$]: 3747.02
MW: 3746.02
Theoretical MW: 3746.58
Match: Approved
Z=1

Peptide LP28 = KKKKKLKKILKKLFQFINQLDNQLQDIKKKKK

HPLC Certificate

HPLC Column	(250×4.6mm I.D.) C18
Detection wavelength	220 nm
Gradient	20-50%B in 30 min
Buffer A	0.05%TFA +2%CH_3CN
Buffer B	0.05%TFA +90%CH_3CN

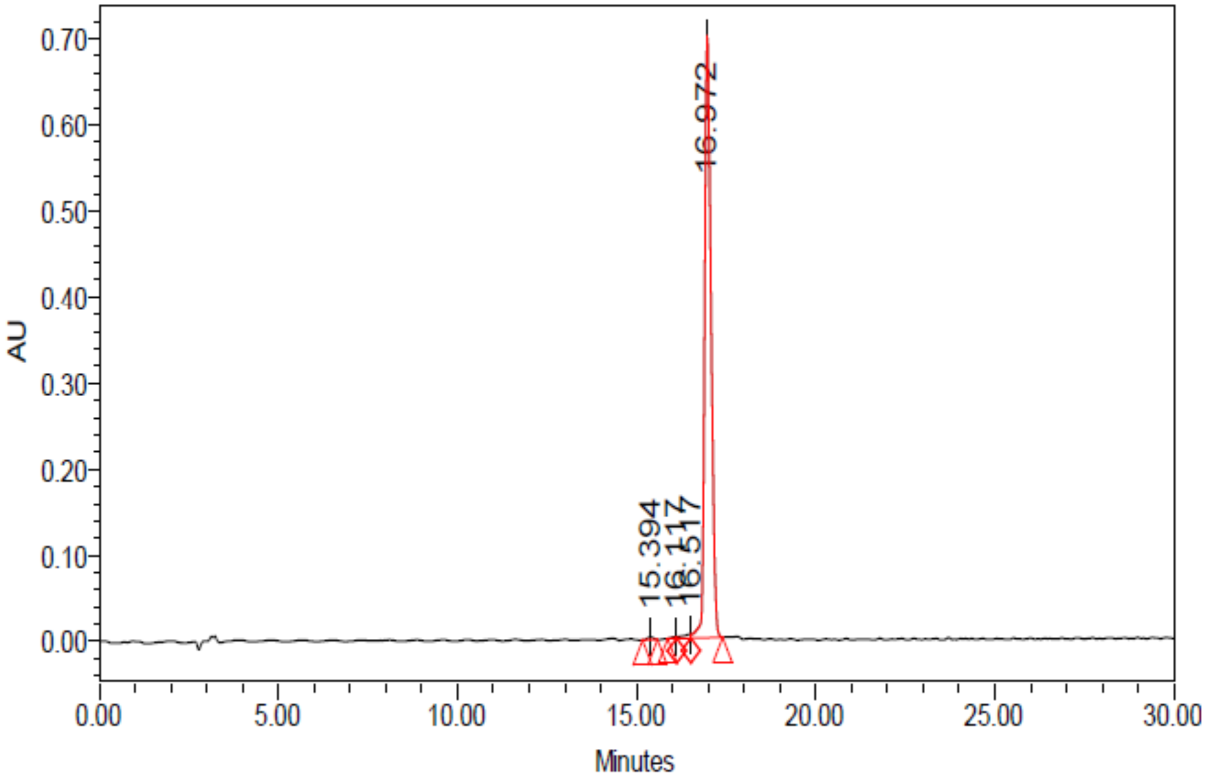

	Retention Time	Area	% Area	Height
1	15.394	36389	0.41	3069
2	16.117	15557	0.18	1673
3	16.517	71712	0.81	4311
4	16.972	8695304	98.60	700056

Mass Spectrometry Certificate

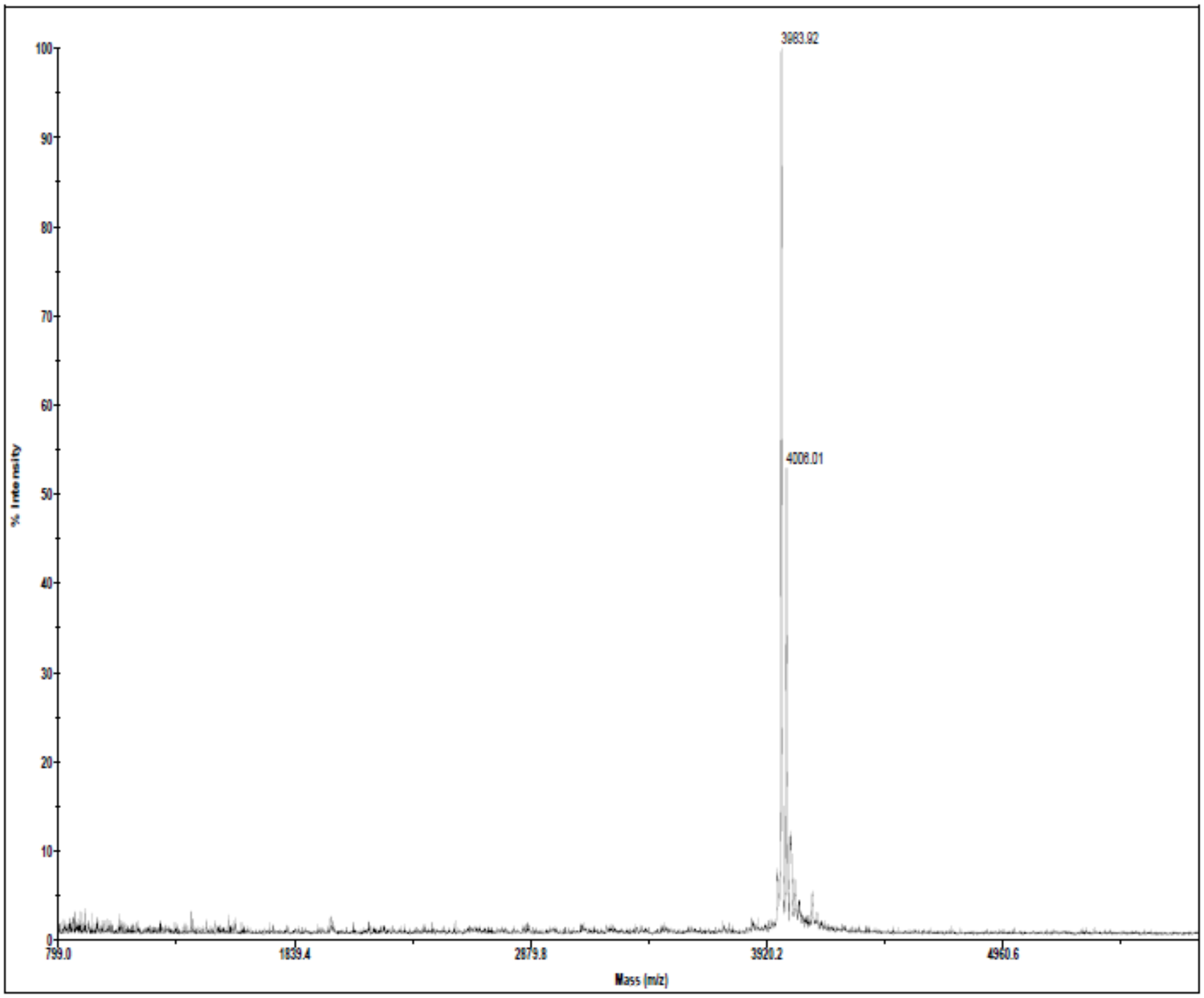

Lot No.: P071220-01-12
Method: MALDI-TOF
Main Peak: 3983.92
MW [M+H$^+$]: 3983.92
MW: 3982.92
Theoretical MW: 3983.03
Match: Approved
Z=1

Peptide LP29 = **KKKK**A**KK**AA**KK**A**F**Q**F**ANQA**D**NQAQ**D**A**K**QN**K**-NH2

HPLC Certificate

HPLC Column	(250×4.6mm I.D.) C18
Detection wavelength	220 nm
Gradient	0-30%B in 30 min
Buffer A	0.05%TFA +2%CH_3CN
Buffer B	0.05%TFA +90%CH_3CN

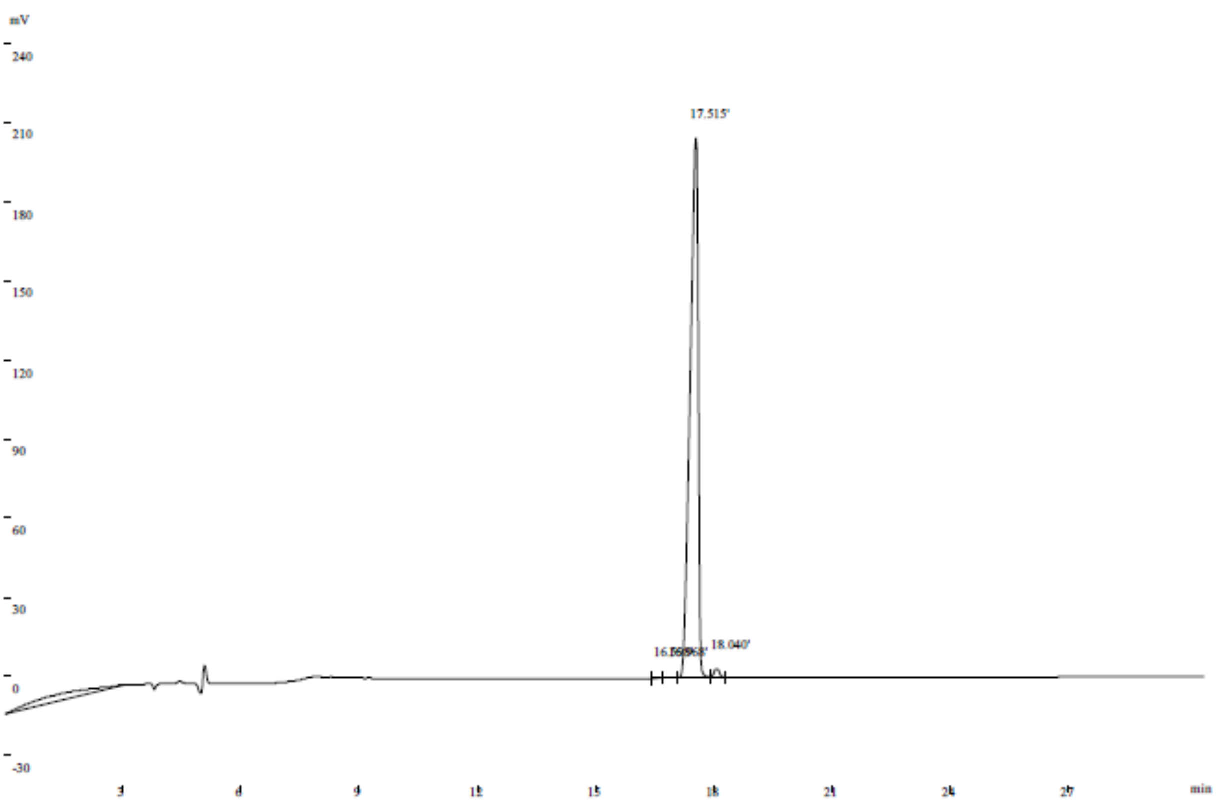

Peak Results

Rank	Time	Quantity	Area	Height
1	16.598	0.1139	3026	306
2	16.968	0.1953	5188	317
3	17.515	98.66	2620577	204732
4	18.040	1.032	27420	3387
Total		100	2656211	208742

Mass Spectrometry Certificate

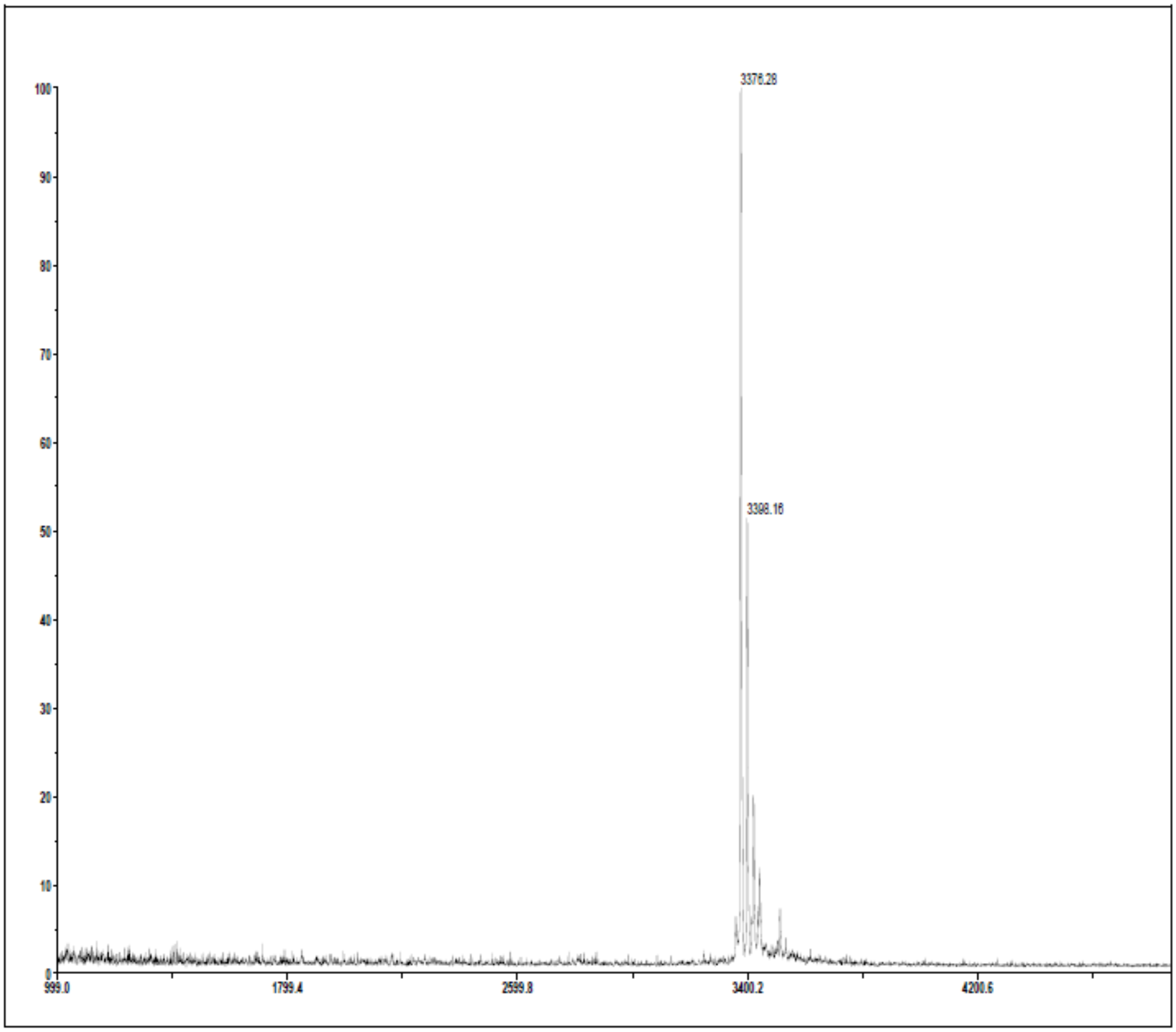

Lot No.: P170521-01-03
Method: MALDI-TOF
Main Peak: 3376.28
MW [M+H$^+$]: 3376.28
MW: 3375.28
Theoretical MW: 3374.93
Match: Approved
Z=1

Peptide LP30 = KKKKMKKMMKKMFQFMNQMDNQMQDMKQNK-NH2

HPLC Certificate

HPLC Column	(250×4.6mm I.D.) C18
Detection wavelength	220 nm
Gradient	18-48%B in 30 min
Buffer A	0.05%TFA +2%CH_3CN
Buffer B	0.05%TFA +90%CH_3CN

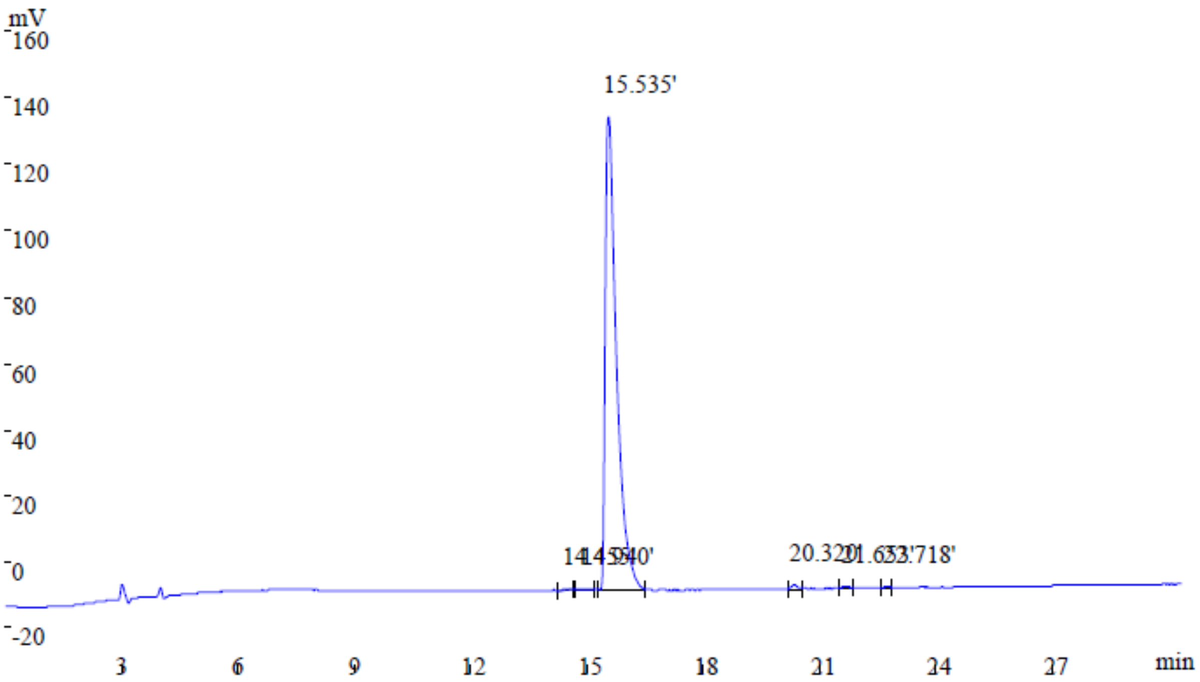

Peak Results

Rank	Time	Conc.	Area	Height
1	14.455	0.08827	2398	319
2	14.940	0.07097	1928	290
3	15.535	99.11	2692266	142301
4	20.320	0.4043	10984	1235
5	21.653	0.2333	6338	700
6	22.718	0.09402	2554	365
Total		100	2716468	145210

Mass Spectrometry Certificate

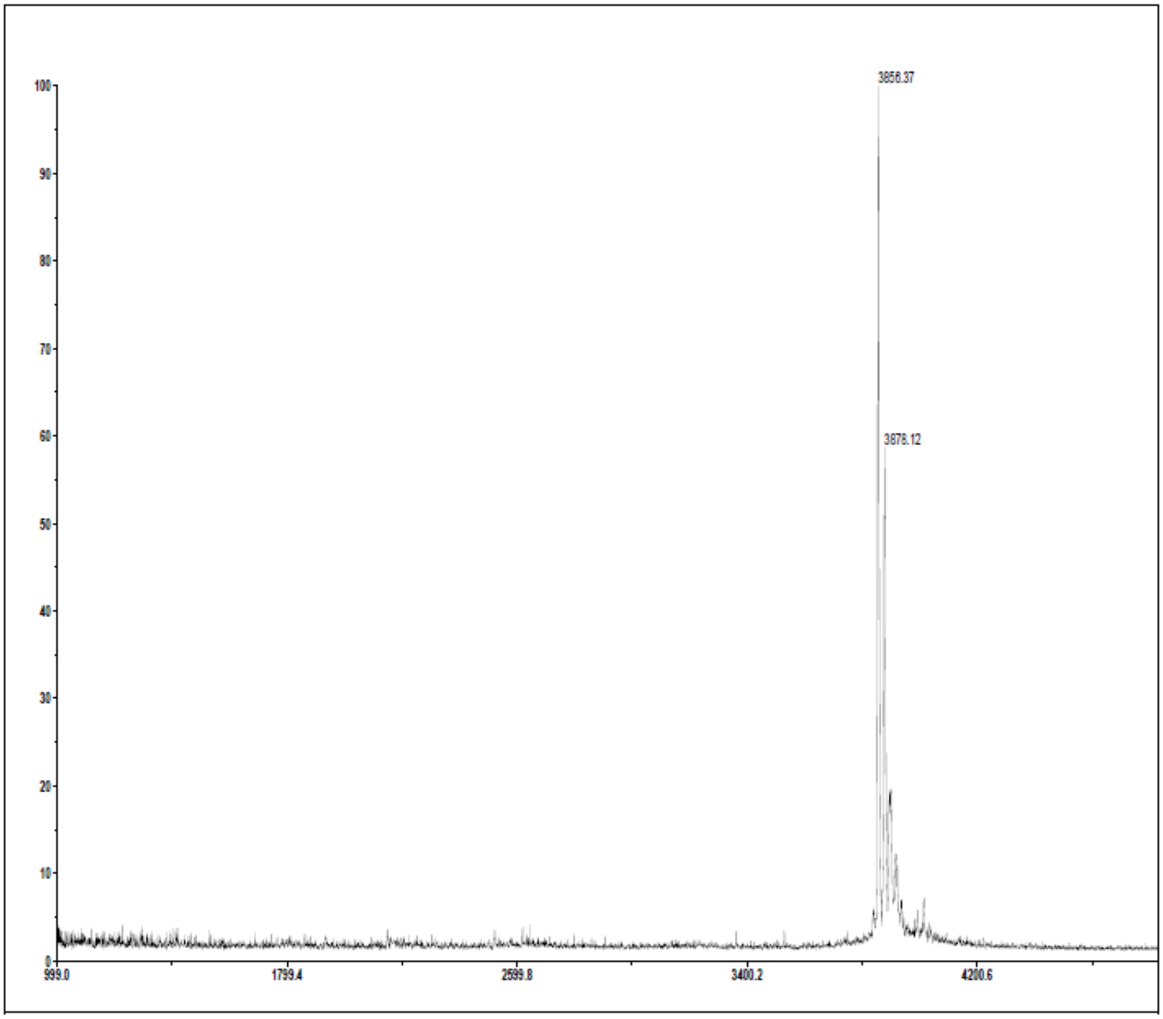

Lot No.: P170521-01-05
Method: MALDI-TOF
Main Peak: 3856.37
MW [M+H$^+$]: 3856.37
MW: 3855.37
Theoretical MW: 3855.87
Match: Approved
Z=1

Peptide LP31 = KKKKVKKVVKKVFQFVNQVDNQVQDVKQNK-NH2

HPLC Certificate

HPLC Column	(250×4.6mm I.D.) C18
Detection wavelength	220 nm
Gradient	12-42%B in 30 min
Buffer A	0.05%TFA +2%CH_3CN
Buffer B	0.05%TFA +90%CH_3CN

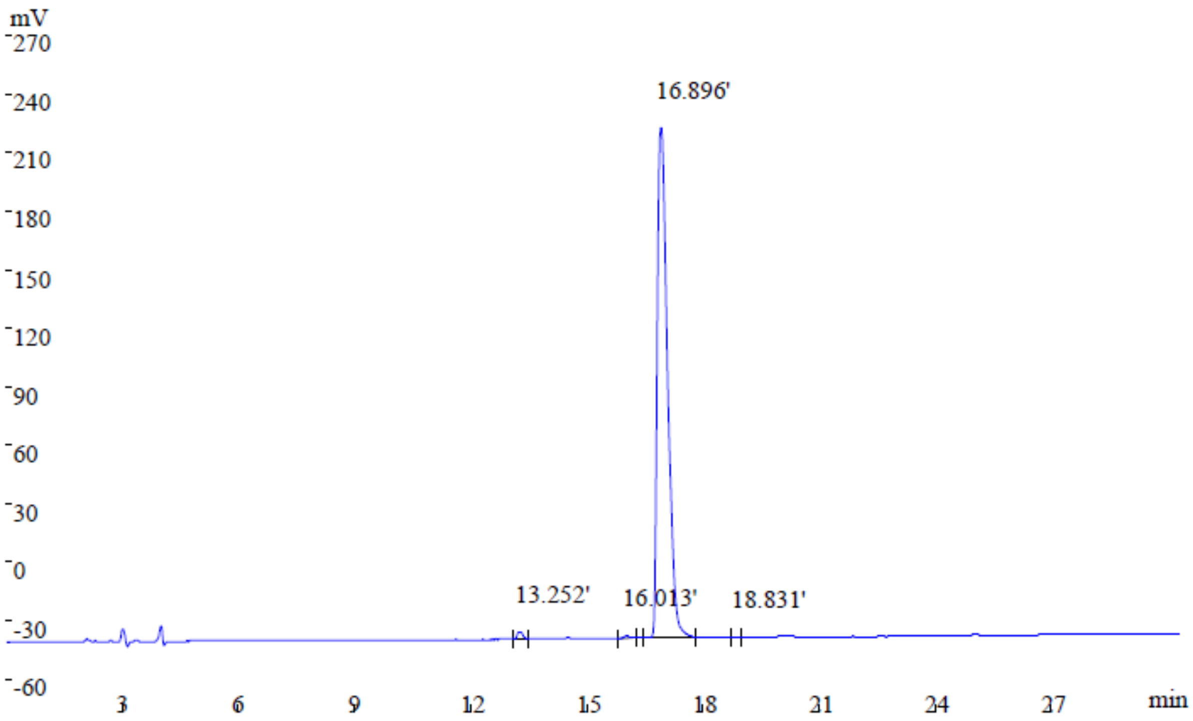

Peak Results

Rank	Time	Conc.	Area	Height
1	13.252	0.7556	34224	3856
2	16.013	0.3018	13669	1077
3	16.896	98.86	4477414	260958
4	18.831	0.08723	3951	490
Total		100	4529258	266381

Mass Spectrometry Certificate

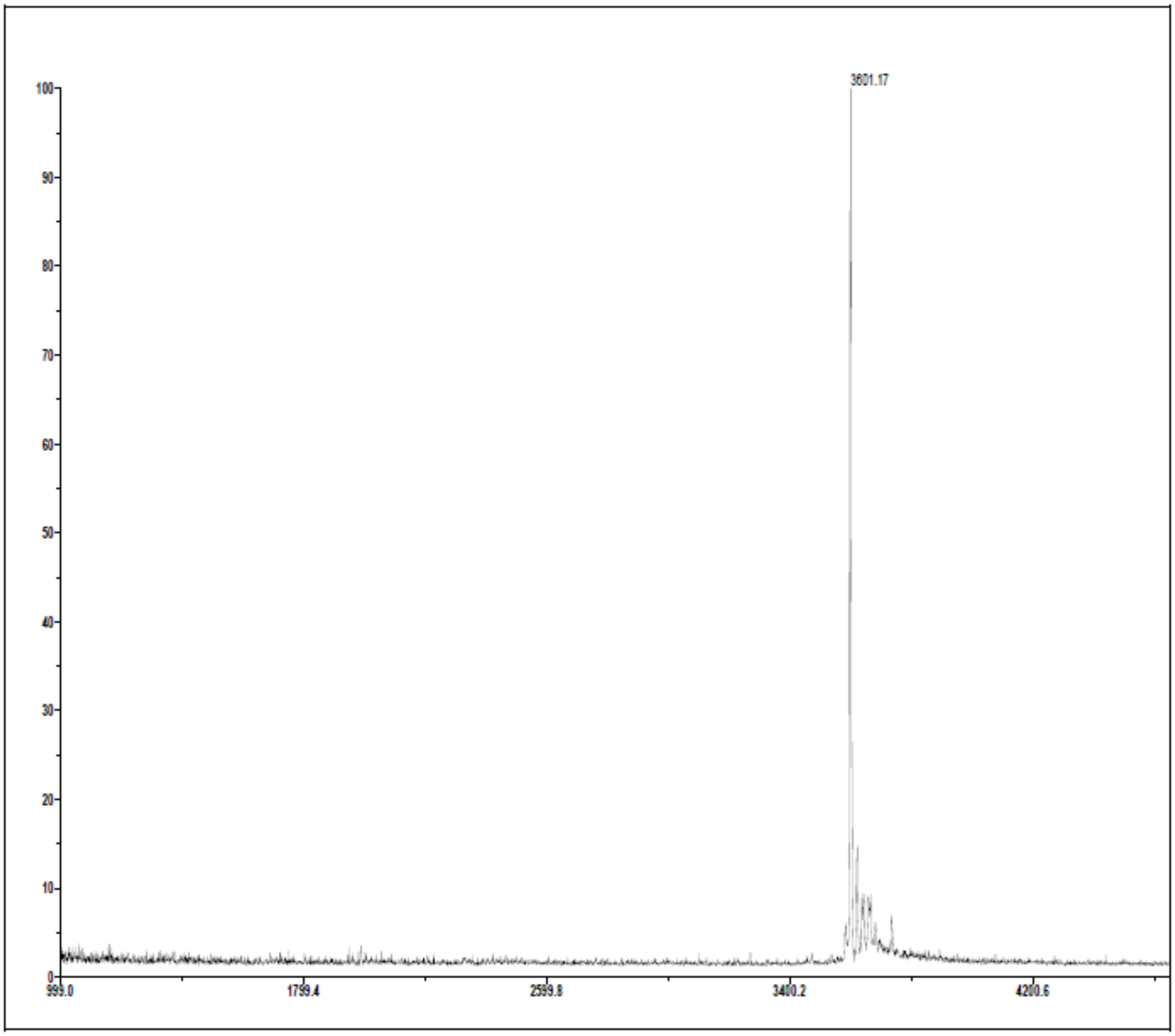

Lot No.: P170521-01-04
Method: MALDI-TOF
Main Peak: 3601.17
MW [M+H$^+$]: 3601.17
MW: 3600.17
Theoretical MW: 3599.36
Match: Approved
Z=1

Peptide LP32 = KKKKLKKLLKKLFQFLNQLDNQLQDLKQNK-NH2

HPLC Certificate

HPLC Column	(250×4.6mm I.D.) C18
Detection wavelength	220 nm
Gradient	20-50%B in 30 min
Buffer A	0.05%TFA +2%CH_3CN
Buffer B	0.05%TFA +90%CH_3CN

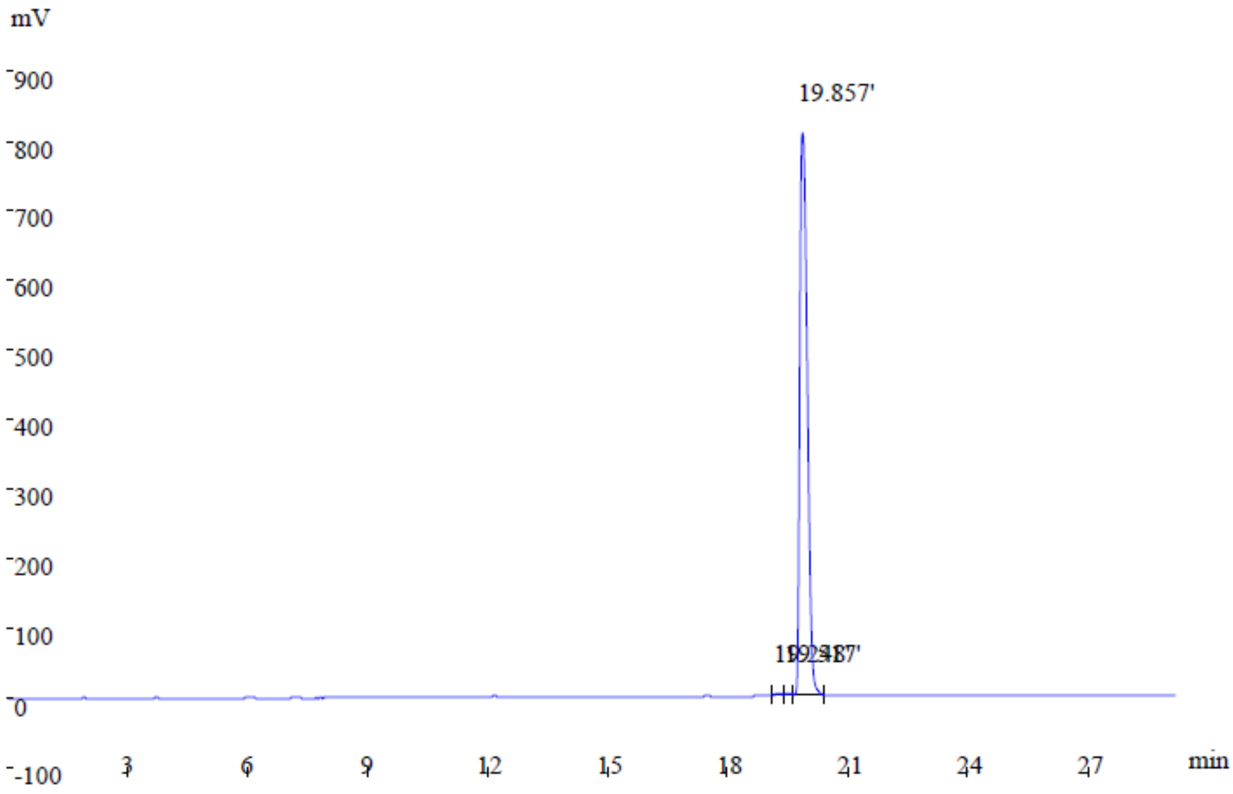

Peak Results

Rank	Time	Conc.	Area	Height
1	19.248	0.1937	18484	1685
2	19.517	0.2373	22644	2596
3	19.857	99.57	9502838	807162
Total		100	9543966	811443

Mass Spectrometry Certificate

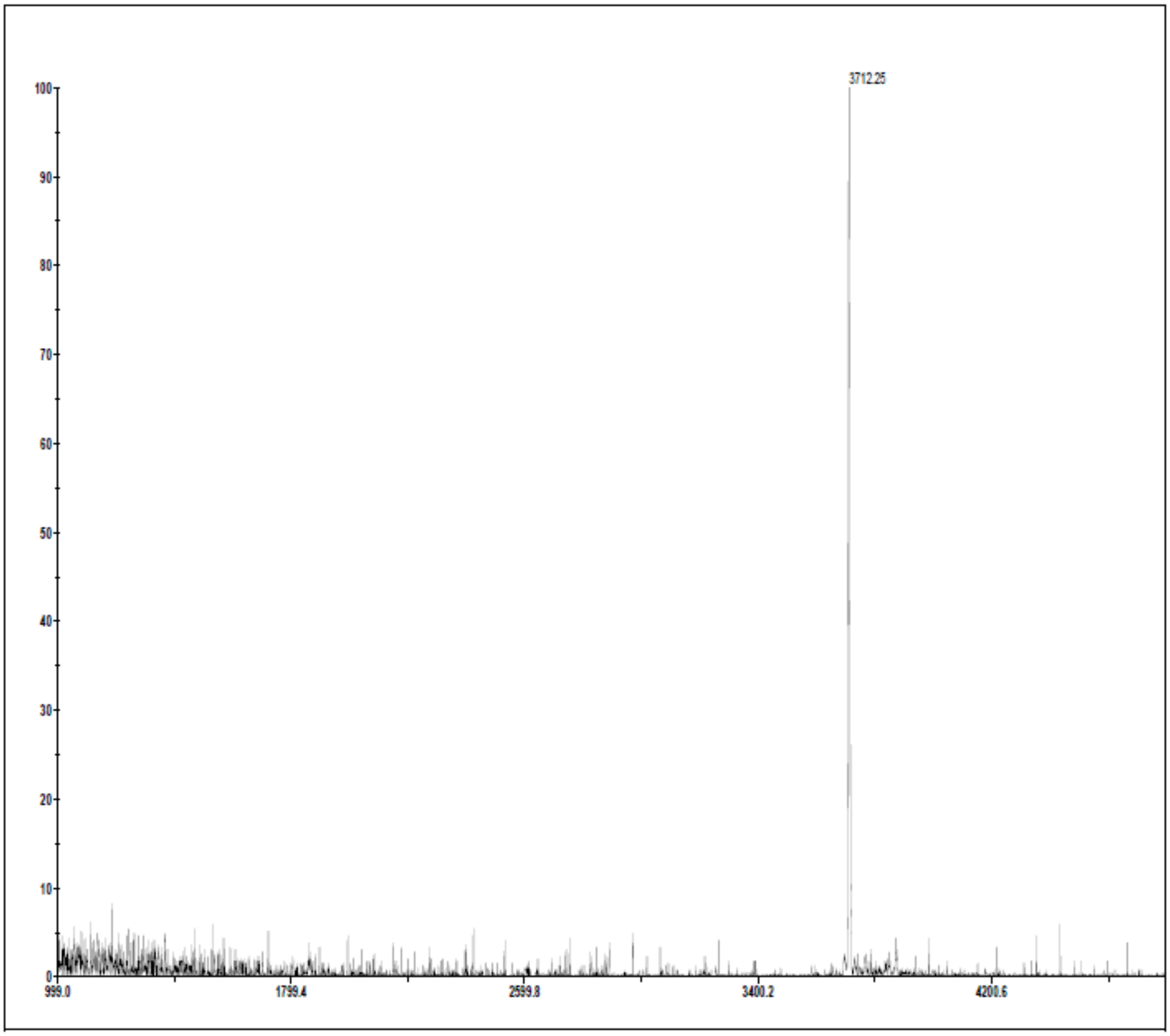

Lot No.: P170521-01-07
Method: MALDI-TOF
Main Peak: 3712.25
MW [M+H$^+$]: 3712.25
MW: 3711.25
Theoretical MW: 3711.58
Match: Approved
Z=1

Peptide LP33 = KKKKIKKIIKKIFQFINQIDNQIQDIKQNK-NH2

HPLC Certificate

HPLC Column	(250×4.6mm I.D.) C18
Detection wavelength	220 nm
Gradient	25-55%B in 30 min
Buffer A	0.05%TFA +2%CH_3CN
Buffer B	0.05%TFA +90%CH_3CN

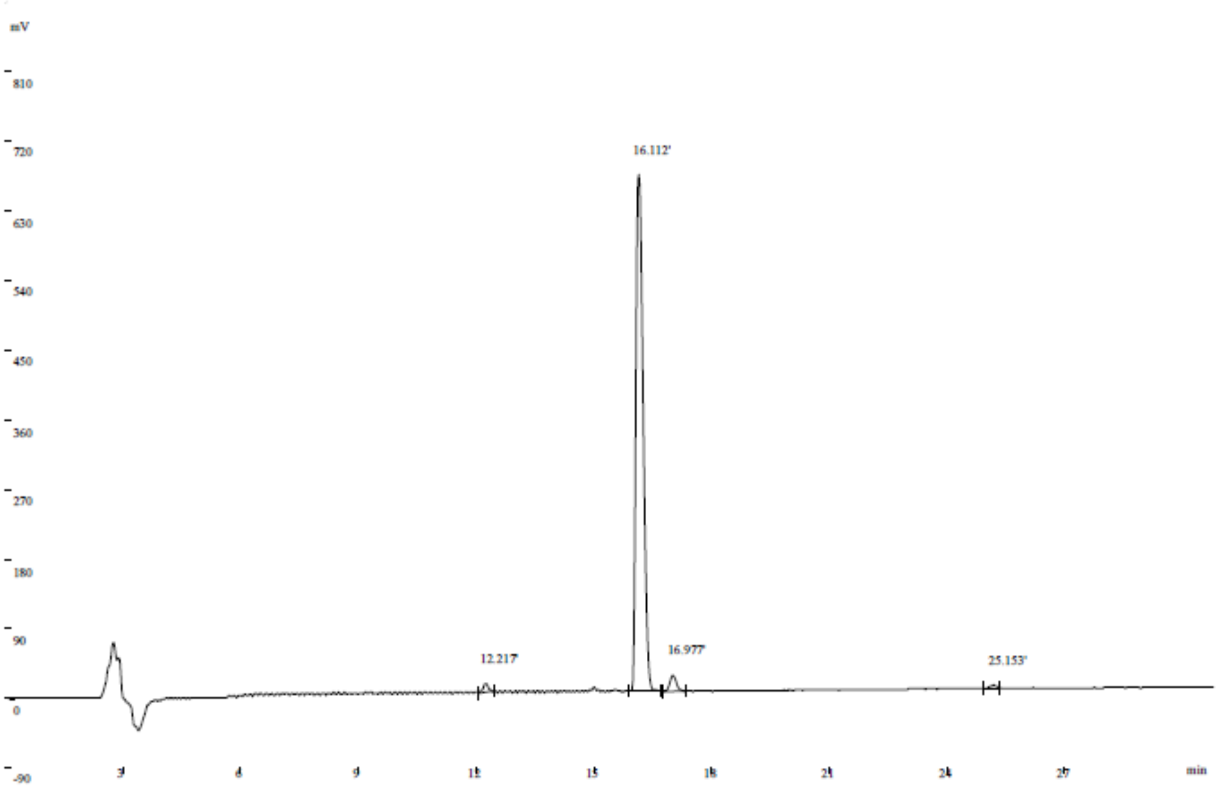

Peak Results

Rank	Time	Quantity	Area	Height
1	12.217	1.009	79496	11083
2	16.112	95.97	7557696	665694
3	16.977	2.502	197023	20544
4	25.153	0.5177	40772	3664
Total		100	7874987	700985

Mass Spectrometry Certificate

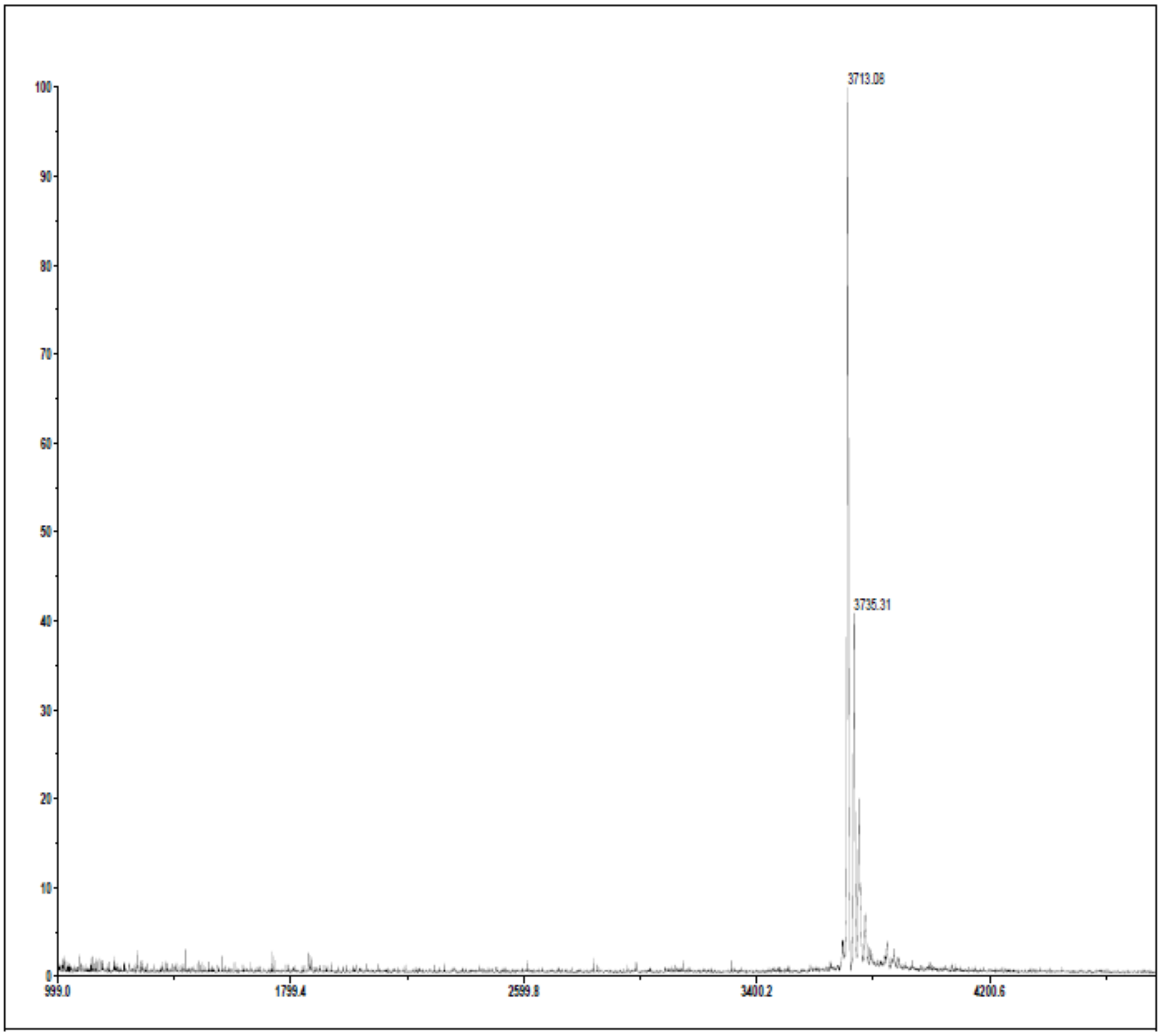

Lot No.: P170521-01-06
Method: MALDI-TOF
Main Peak: 3713.08
MW [M+H$^+$]: 3713.08
MW: 3712.08
Theoretical MW: 3711.58
Match: Approved
Z=1

Peptide LP34 = KKKKLKKILKKLFTFITTLDTTLTDIKTTK-NH2

HPLC Certificate

Lot No.	P170521-01-08
HPLC Column	(250×4.6mm I.D.) C18
Detection wavelength	220 nm
Gradient	28-58%B in 30 min
Buffer A	0.05%TFA +2%CH_3CN
Buffer B	0.05%TFA +90%CH_3CN

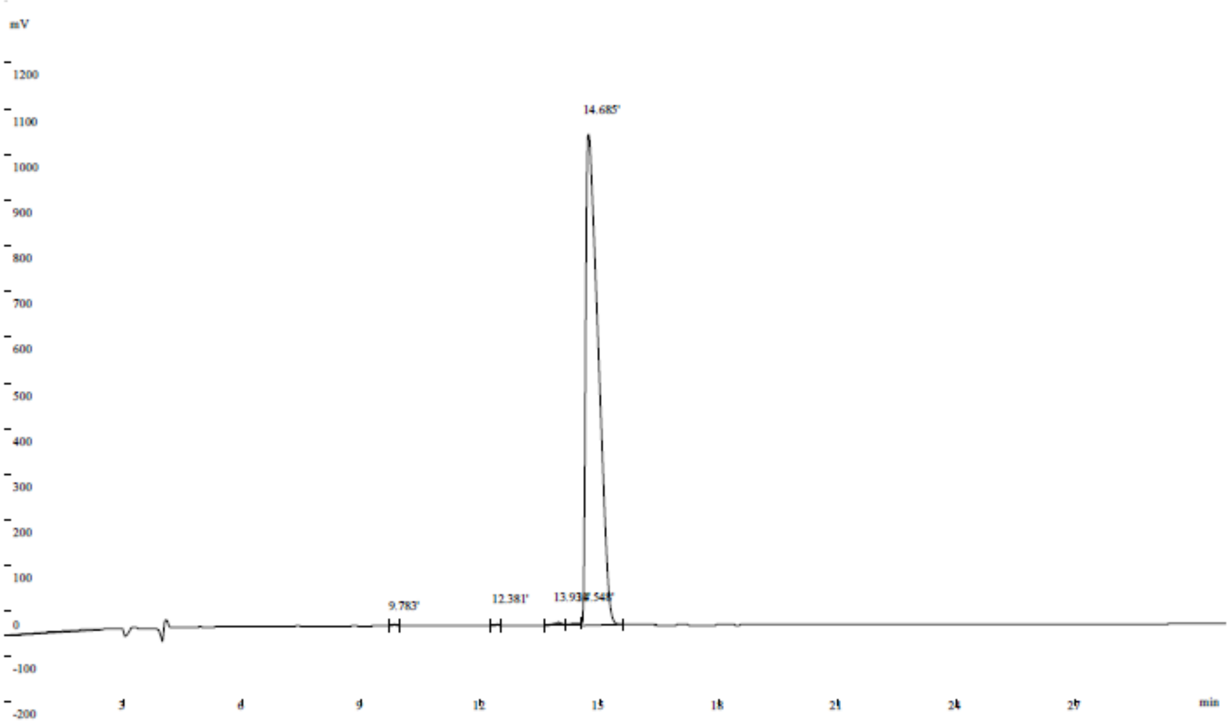

Peak Results

Rank	Time	Quantity	Area	Height
1	9.783	0.07631	16802	2228
2	12.381	0.04537	9990	1415
3	13.931	0.3482	76675	5647
4	14.548	0.3739	82326	6588
5	14.685	99.16	21831341	1072853
Total		100	22017134	1088731

Mass Spectrometry Certificate

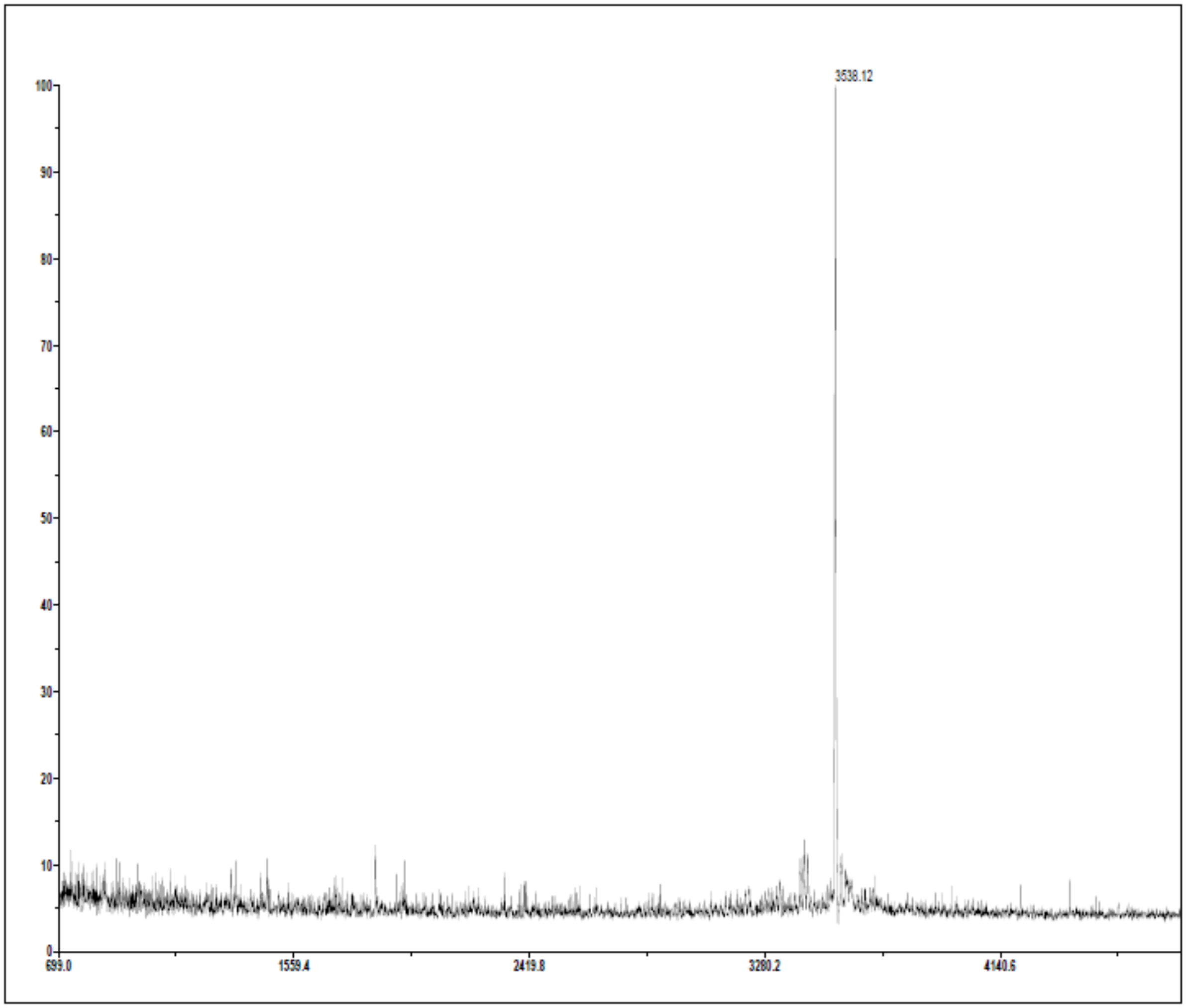

Lot No.: P170521-01-08
Method: MALDI-TOF
Main Peak: 3538.12
MW [M+H$^+$]: 3538.12
MW: 3537.12
Theoretical MW: 3537.45
Match: Approved
Z=1

Peptide LP35 = KKKKLKKILKKLFQFIQQLDQQLQDIKQQK-NH2

HPLC Certificate

HPLC Column	(250×4.6mm I.D.) C18
Detection wavelength	220 nm
Gradient	20-50%B in 30 min
Buffer A	0.05%TFA +2%CH_3CN
Buffer B	0.05%TFA +90%CH_3CN

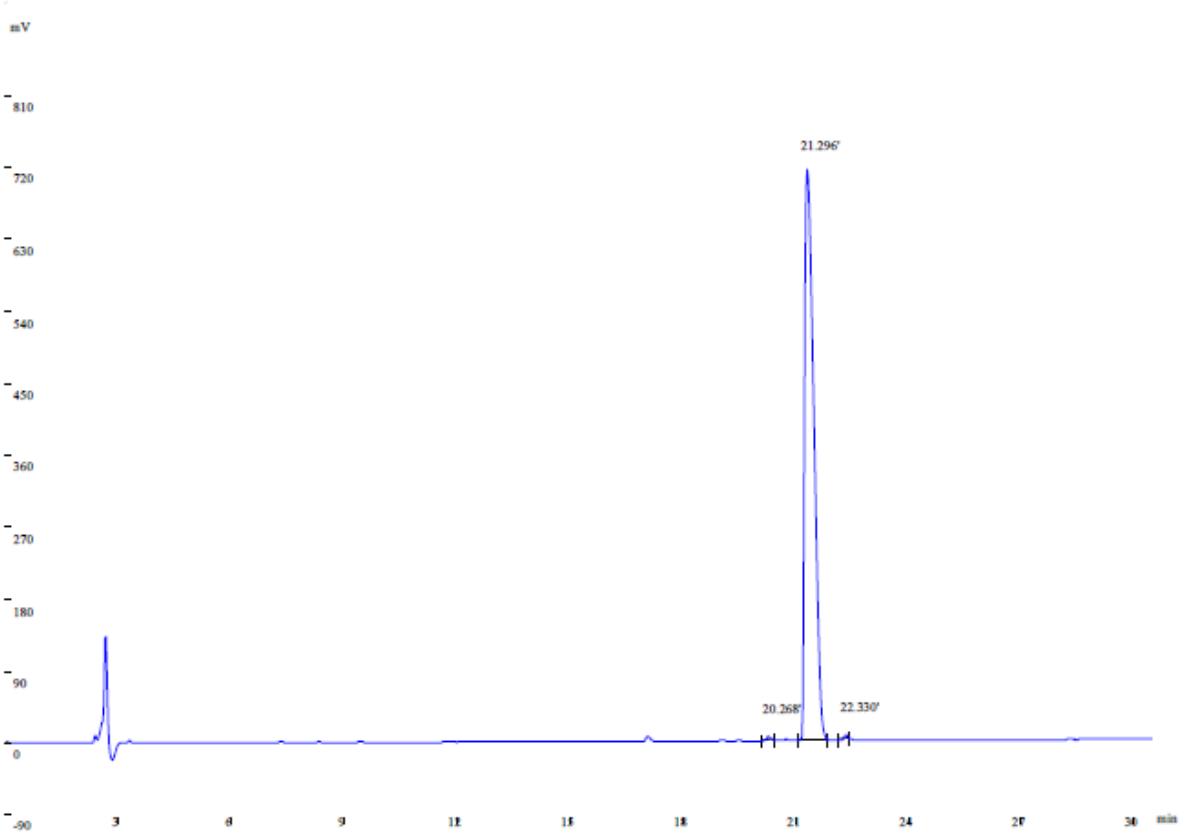

Peak Results

Rank	Time	Conc.	Area	Height
1	20.268	0.3373	41225	5315
2	21.296	99.37	12145670	714712
3	22.330	0.2875	35142	5714
Total		100	12222037	725741

<u>**Mass Spectrometry Certificate**</u>

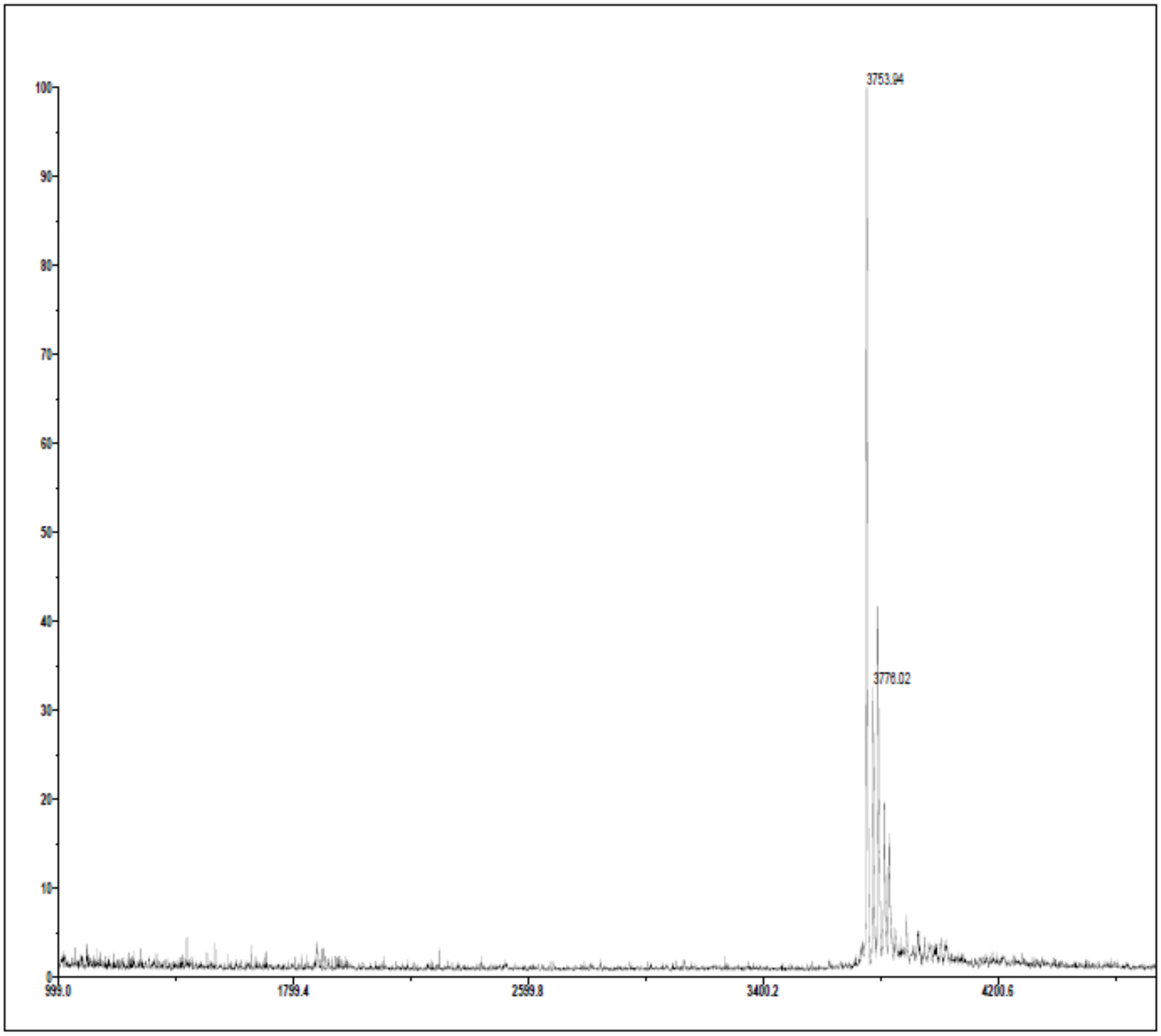

Lot No.: P170521-01-10
Method: MALDI-TOF
Main Peak: 3753.94
MW [M+H$^+$]: 3753.94
MW: 3752.94
Theoretical MW: 3753.66
Match: Approved
Z=1

Peptide LP36 = KKKKLKKILKKLFSFISSLDSSLSDIKSSK-NH2

HPLC Certificate

HPLC Column	(250×4.6mm I.D.) C18
Detection wavelength	220 nm
Gradient	20-50%B in 30 min
Buffer A	0.05%TFA +2%CH_3CN
Buffer B	0.05%TFA +90%CH_3CN

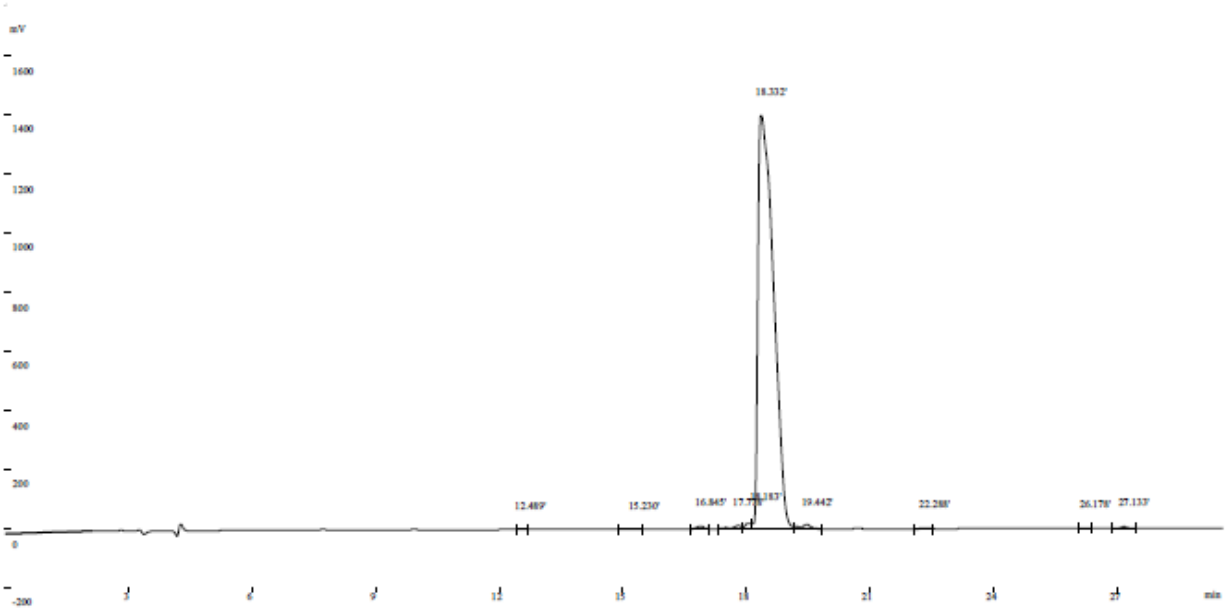

Peak Results

Rank	Time	Quantity	Area	Height
1	12.489	0.02091	7372	967
2	15.230	0.08147	28719	1900
3	16.845	0.2572	90663	8688
4	17.778	0.4167	146880	10146
5	18.183	0.5708	201204	27388
6	18.332	97.83	34486470	1398540
7	19.442	0.5819	205146	13246
8	22.288	0.04685	16516	1385
9	26.178	0.03691	13012	1600
10	27.133	0.1577	55609	5305
Total		100	35251591	1469165

Mass Spectrometry Certificate

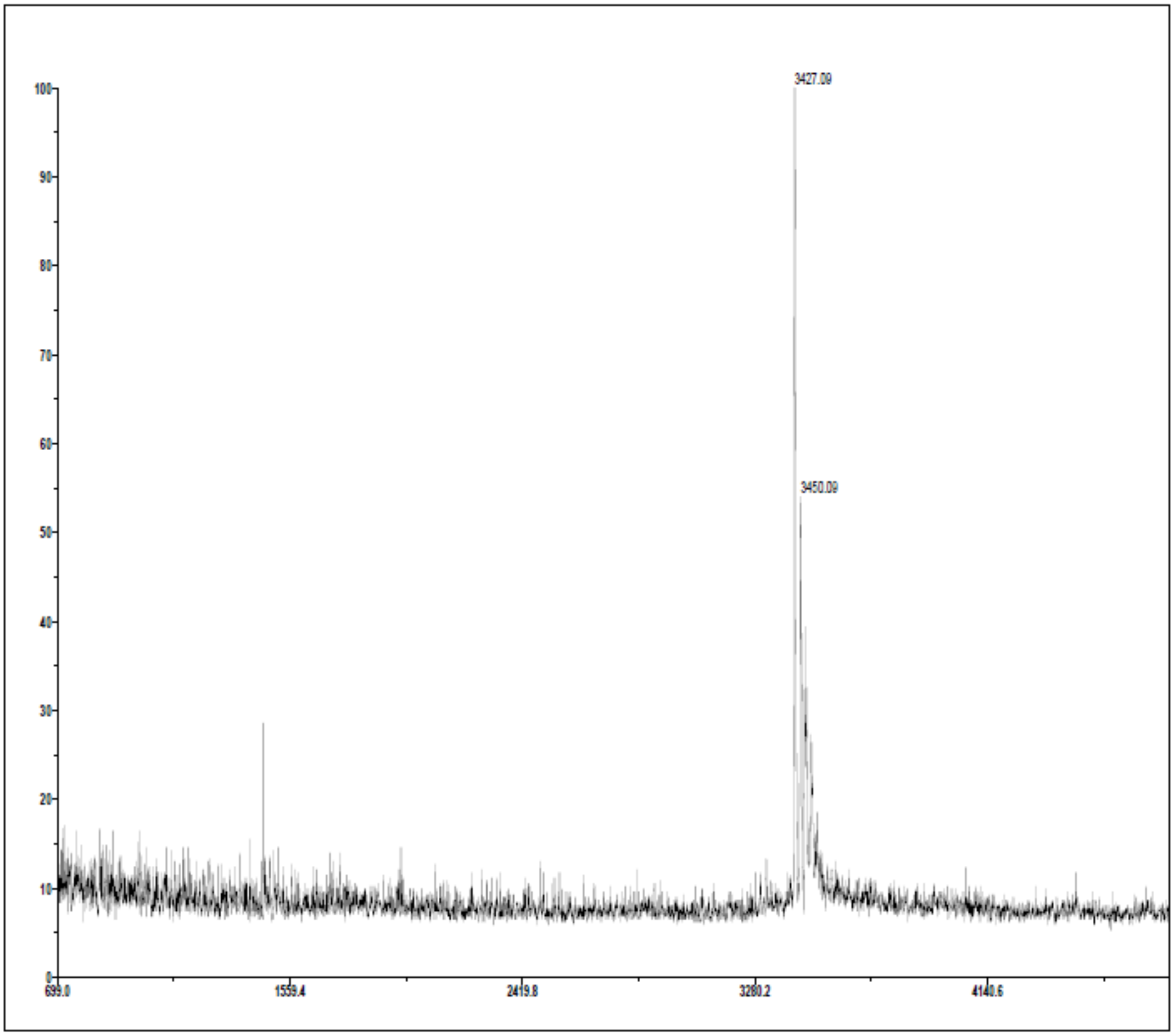

Lot No.: P170521-01-09
Method: MALDI-TOF
Main Peak: 3427.09
MW [M+H$^+$]: 3427.09
MW: 3426.09
Theoretical MW: 3425.23
Match: Approved
Z=1

Peptide LP37 = KKKKLKKILKKLFNFINNLDNNLNDIKNNK-NH2

HPLC Certificate

HPLC Column	(250×4.6mm I.D.) C18
Detection wavelength	220 nm
Gradient	20-50%B in 30 min
Buffer A	0.05%TFA +2%CH_3CN
Buffer B	0.05%TFA +90%CH_3CN

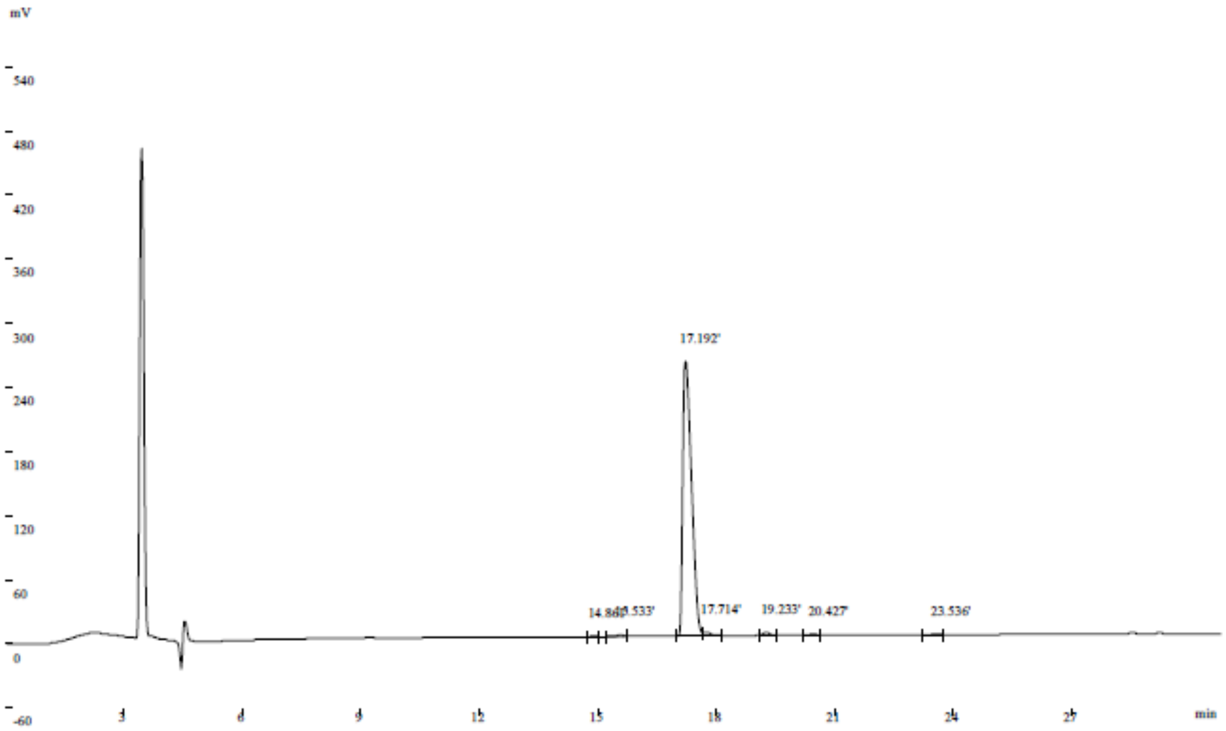

Peak Results

Rank	Time	Quantity	Area	Height
1	14.861	0.1074	3964	537
2	15.533	0.4005	14779	1073
3	17.192	97.39	3594154	256822
4	17.714	0.9335	34447	2951
5	19.233	0.6383	23556	2258
6	20.427	0.2717	10027	1041
7	23.536	0.2546	9396	1042
Total		100	3690323	265724

Mass Spectrometry Certificate

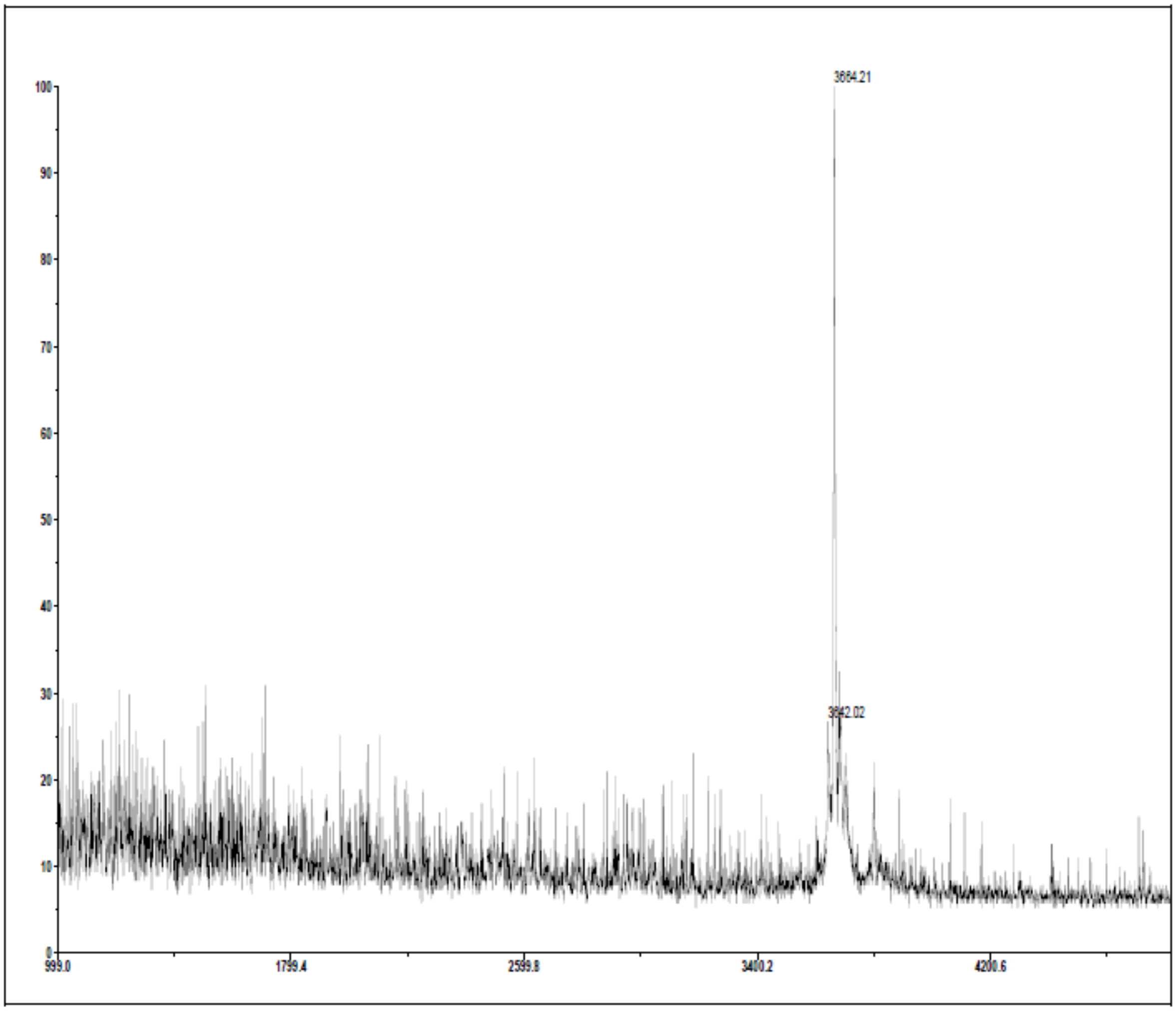

Lot No.: P170521-01-11
Method: MALDI-TOF
Main Peak: 3664.21
MW [M+Na^{+}]: 3664.21
MW: 3641.21
Theoretical MW: 3641.44
Match: Approved
Z=1

Peptide LP38 = KKKKLRRILRRLFQFINQLDNQLQDIKQNK-NH2

HPLC Certificate

**HPLC Column**	(250×4.6mm I.D.) C18
**Detection wavelength**	220 nm
**Gradient**	30-60%B in 30 min
**Buffer A**	0.05%TFA +2%CH_3CN
**Buffer B**	0.05%TFA +90%CH_3CN

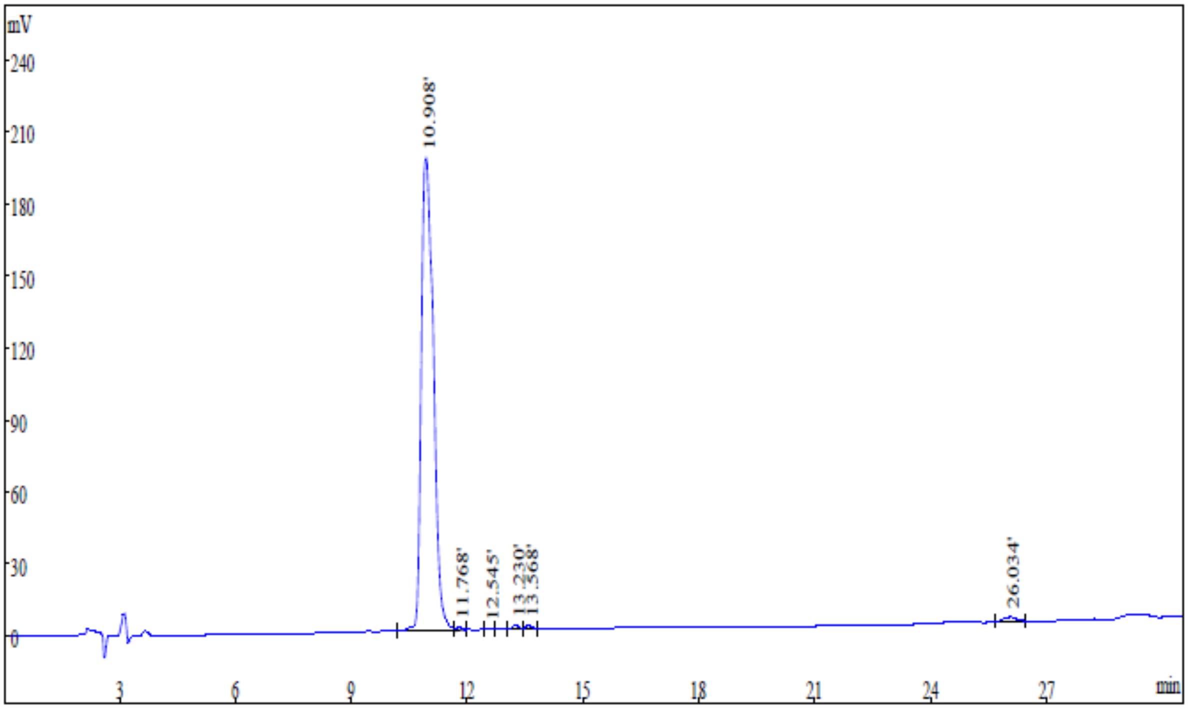

Peak Results

Rank	Time	Conc.	Area	Height
1	10.908	97.92	3970979	196425
2	11.768	0.1393	5649	728
3	12.545	0.05034	2042	334
4	13.230	0.4941	20036	2249
5	13.568	0.5128	20795	2241
6	26.034	0.8817	35753	1602
Total		100	4055254	203579

<u>**Mass Spectrometry Certificate**</u>

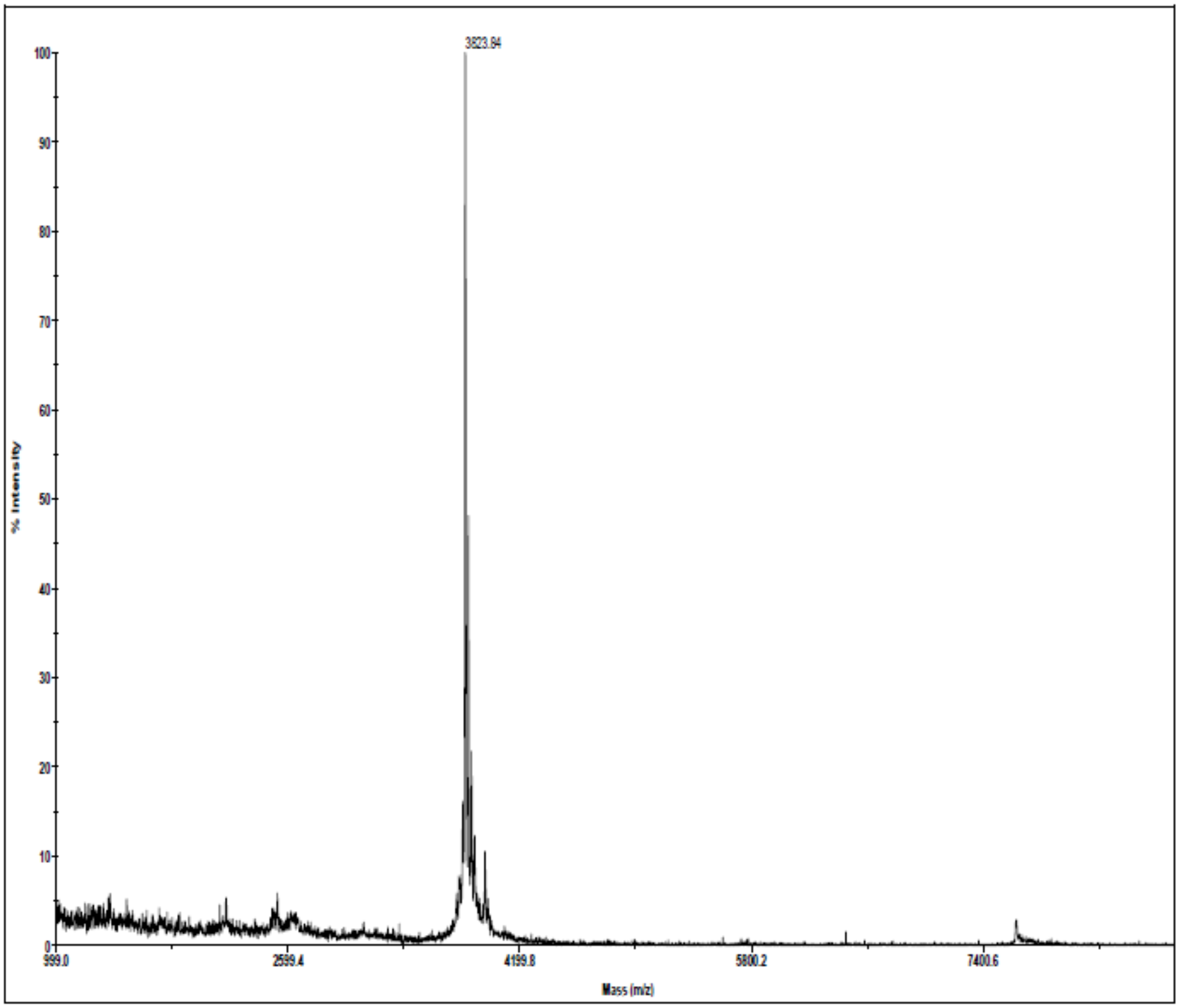

Lot No.: P300720-01-06
Method: MALDI-TOF
Main Peak: 3823.84
MW [M+H^+]: 3823.84
MW: 3822.84
Theoretical MW: 3823.63
Match: Approved
Z=1

Peptide LP39 = RRRRLKKILKKLFQFINQLDNQLQDIRQNR-NH2

HPLC Certificate

**HPLC Column**	(250×4.6mm I.D.) C18
**Detection wavelength**	220 nm
**Gradient**	22-52%B in 30 min
**Buffer A**	0.05%TFA +2%CH_3CN
**Buffer B**	0.05%TFA +90%CH_3CN

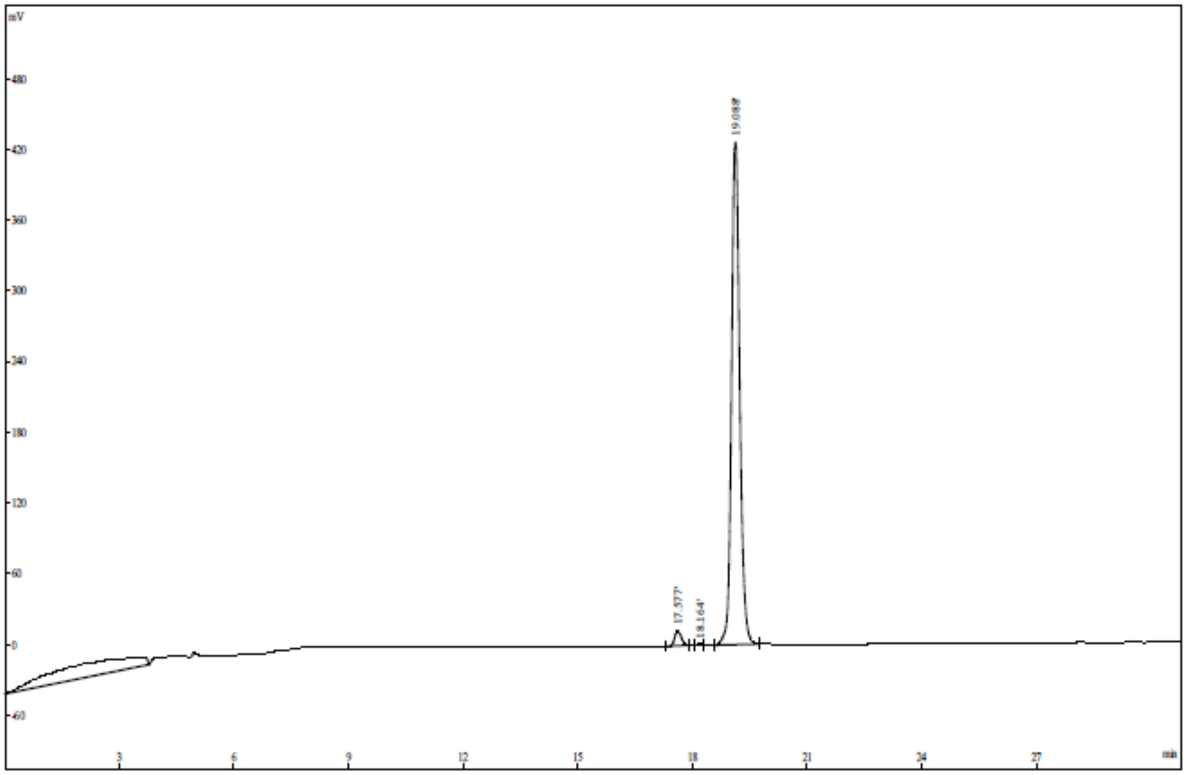

Peak Results

Rank	Time	Quantity	Area	Height
1	17.577	2.222	130904	12313
2	18.164	0.1141	6721	757
3	19.088	97.66	5752715	425024
Total		100	5890340	438094

Mass Spectrometry Certificate

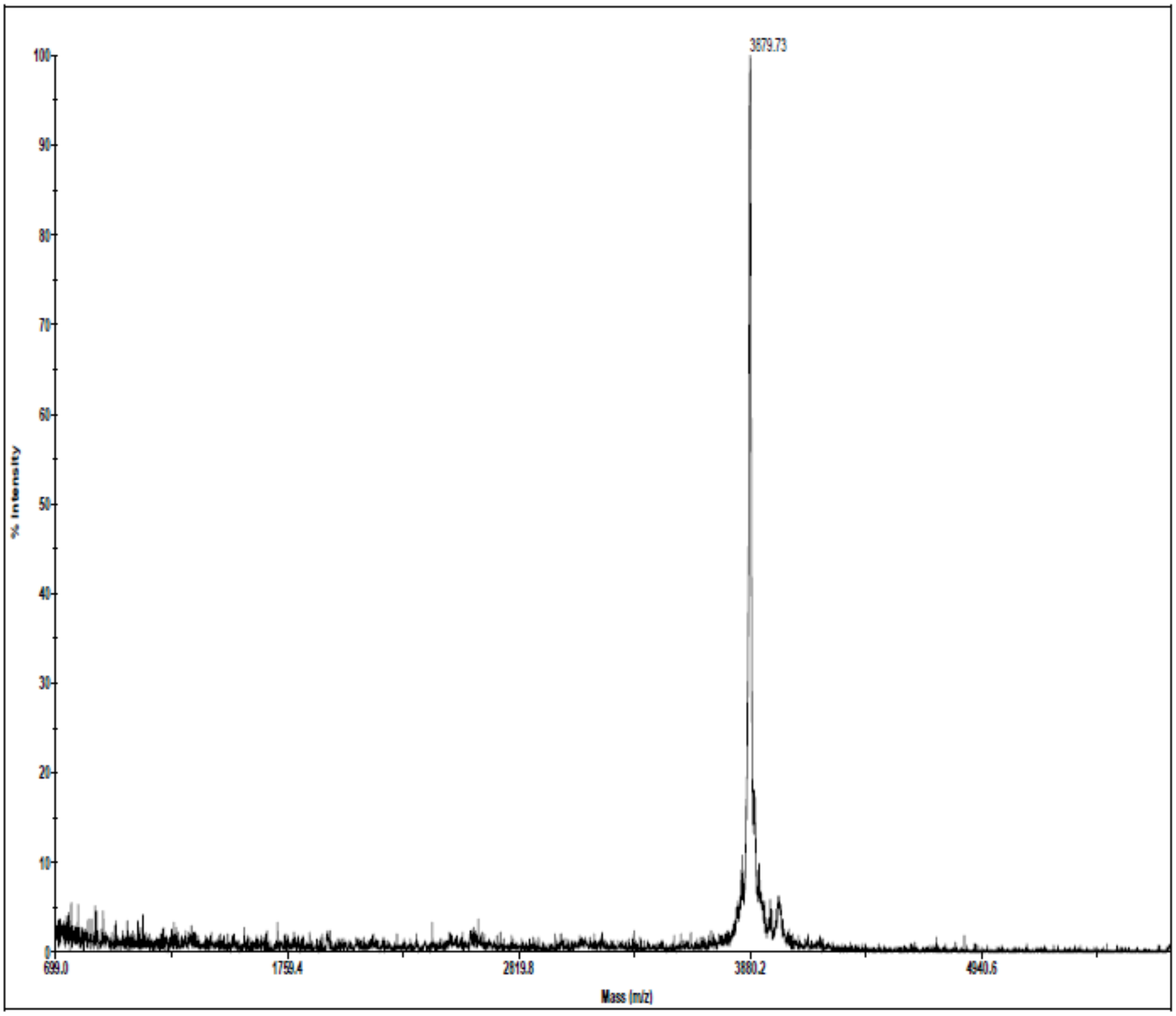

Lot No.: P300720-01-07
Method: MALDI-TOF
Main Peak: 3879.73
MW [M+H$^+$]: 3879.73
MW: 3878.73
Theoretical MW: 3879.66
Match: Approved
Z=1

Peptide LP40 = HHHHLKKILKKLFQFINQLDNQLQDIHHHH

HPLC Certificate

HPLC Column	(250×4.6mm I.D.) C18
Detection wavelength	220 nm
Gradient	30-60%B in 30 min
Buffer A	0.05%TFA +2%CH_3CN
Buffer B	0.05%TFA +90%CH_3CN

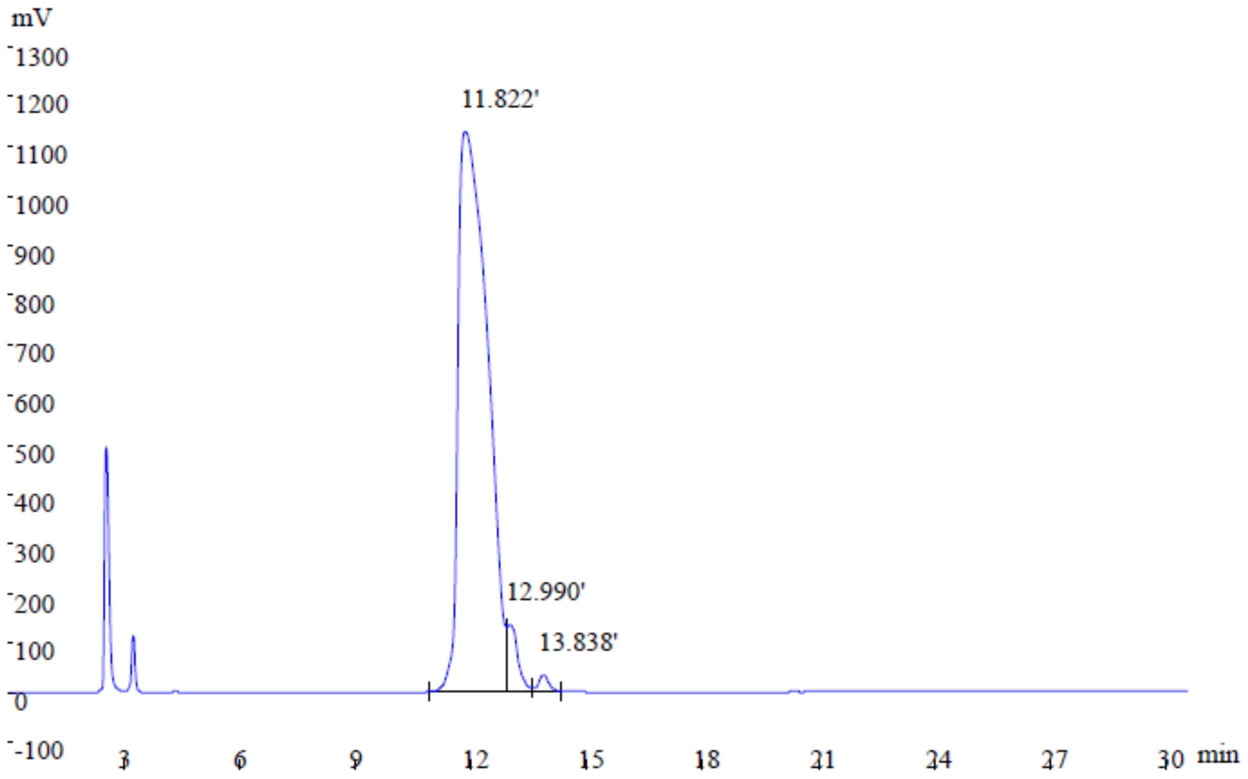

Peak Results

Rank	Time	Conc.	Area	Height
1	11.822	95.21	58034144	1126757
2	12.990	3.883	2366751	132323
3	13.838	0.9108	555207	33077
Total		100	60956102	1292157

Mass Spectrometry Certificate

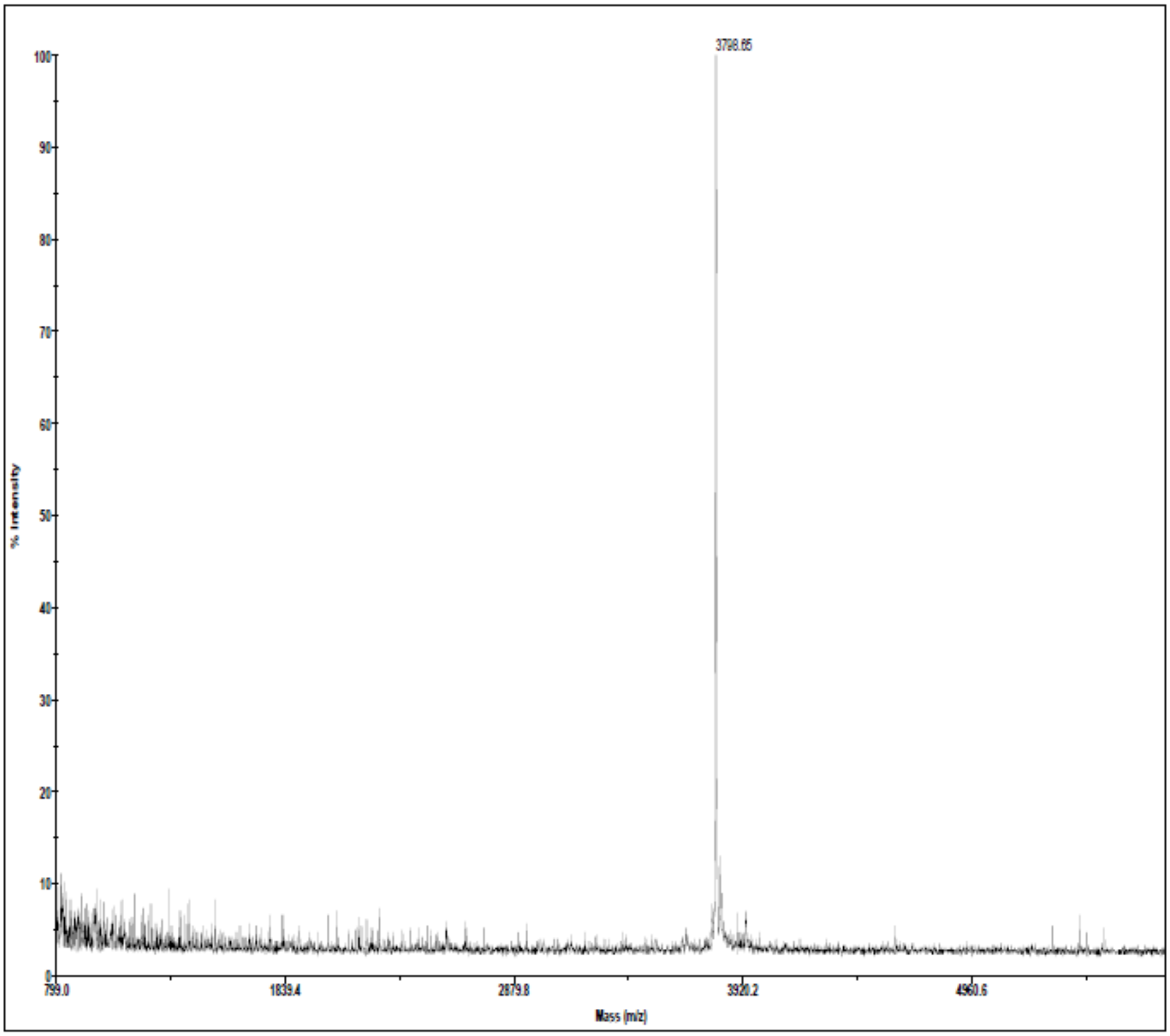

Lot No.: P071220-01-09
Method: MALDI-TOF
Main Peak: 3798.65
MW [M+H$^+$]: 3798.65
MW: 3797.65
Theoretical MW: 3798.41
Match: Approved
Z=1

Peptide LP43 = KNQNLDDILNQLFQFINQLKNQLQKIKKKK-NH2

HPLC Certificate

HPLC Column	(250×4.6mm I.D.) C18
Detection wavelength	220 nm
Gradient	8-38%B in 30 min
Buffer A	0.05%TFA +2%CH_3CN
Buffer B	0.05%TFA +90%CH_3CN

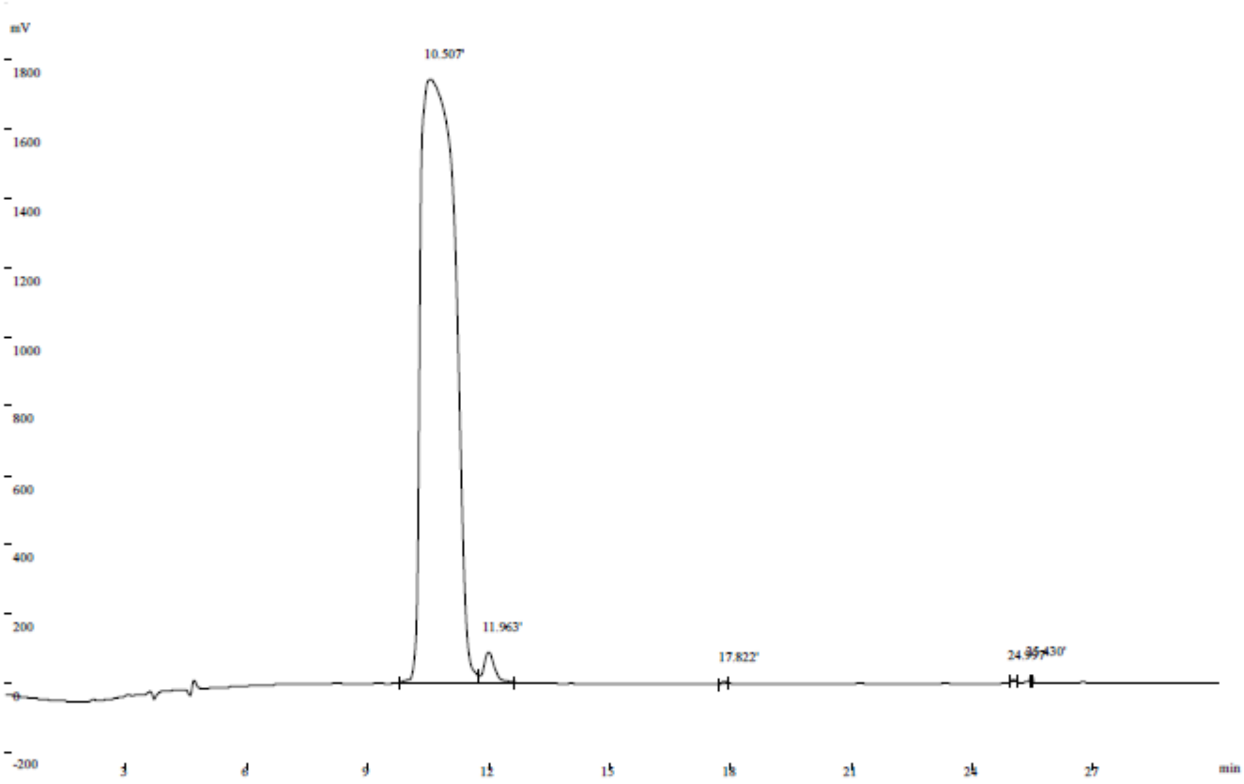

Peak Results

Rank	Time	Quantity	Area	Height
1	10.507	98.37	98711642	1741216
2	11.963	1.511	1516068	87484
3	17.822	0.03916	39295	5295
4	24.997	0.03865	38780	6517
5	25.430	0.03843	38565	14819
Total		100	100344350	1855331

Mass Spectrometry Certificate

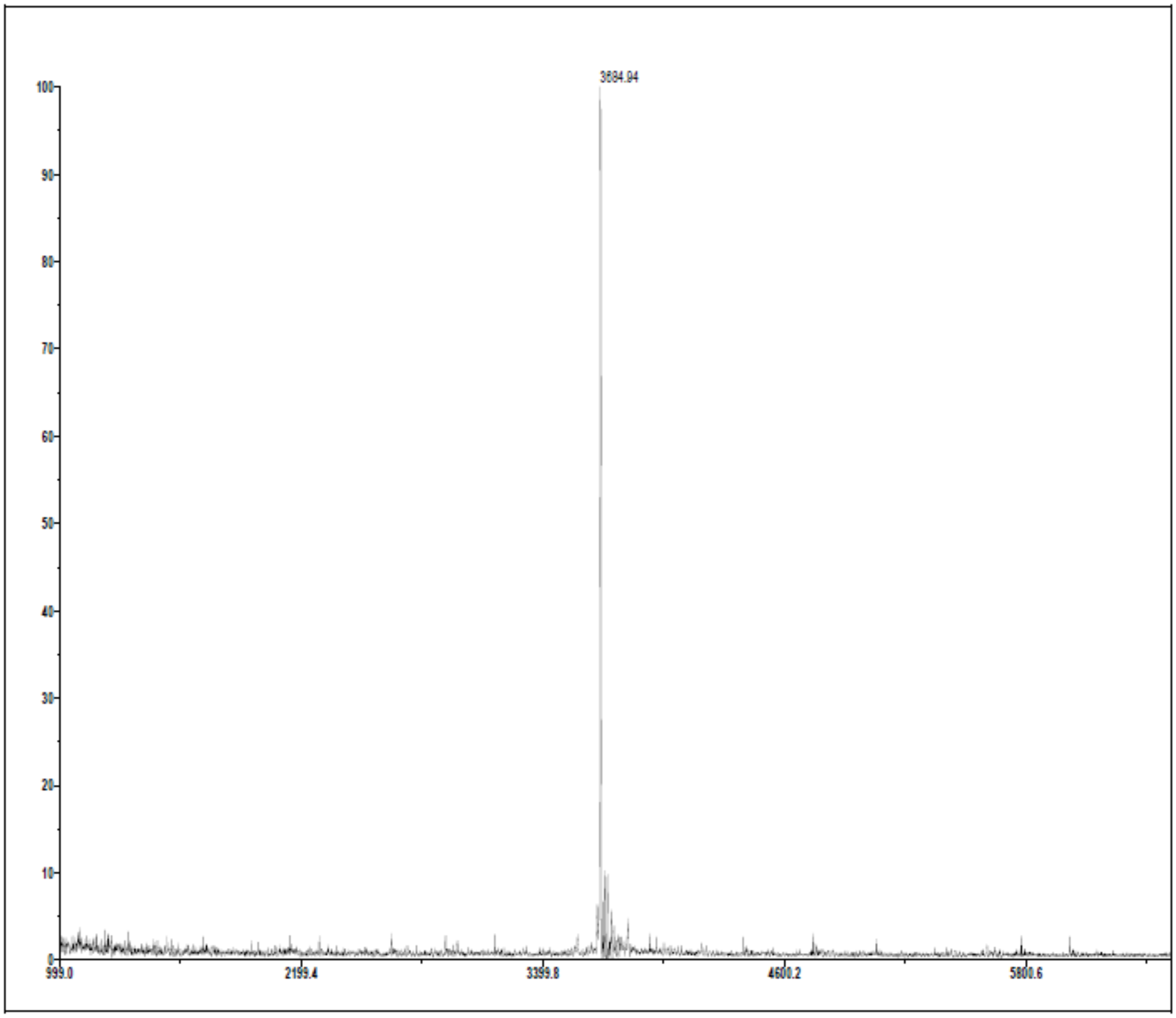

Lot No.: P061021-02-07
Method: MALDI-TOF
Main Peak: 3684.94
MW [M+H$^+$]: 3684.94
MW: 3683.94
Theoretical MW: 3683.39
Match: Approved
Z=1

Peptide Melittin = GIGAVLKVLTTGLPALISWIKRKRQQ-NH2

HPLC Certificate

**HPLC Column**	(250×4.6mm I.D.) C18
**Detection wavelength**	220 nm
**Gradient**	28-58%B in 30 min
**Buffer A**	0.05%TFA +2%CH_3CN
**Buffer B**	0.05%TFA +90%CH_3CN

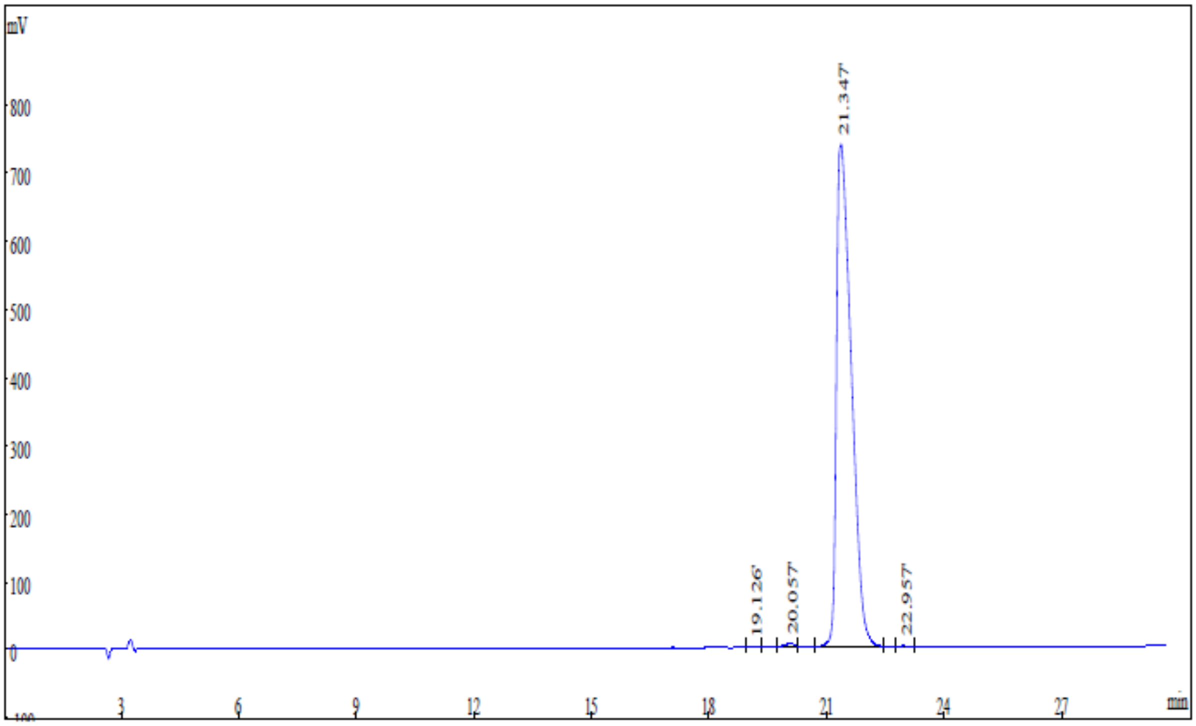

Peak Results

Rank	Time	Conc.	Area	Height
1	19.126	0.09264	17118	1252
2	20.057	0.3664	67706	4298
3	21.347	99.46	18376733	735029
4	22.957	0.08532	15766	1323
Total		100	18477323	741902

Mass Spectrometry Certificate

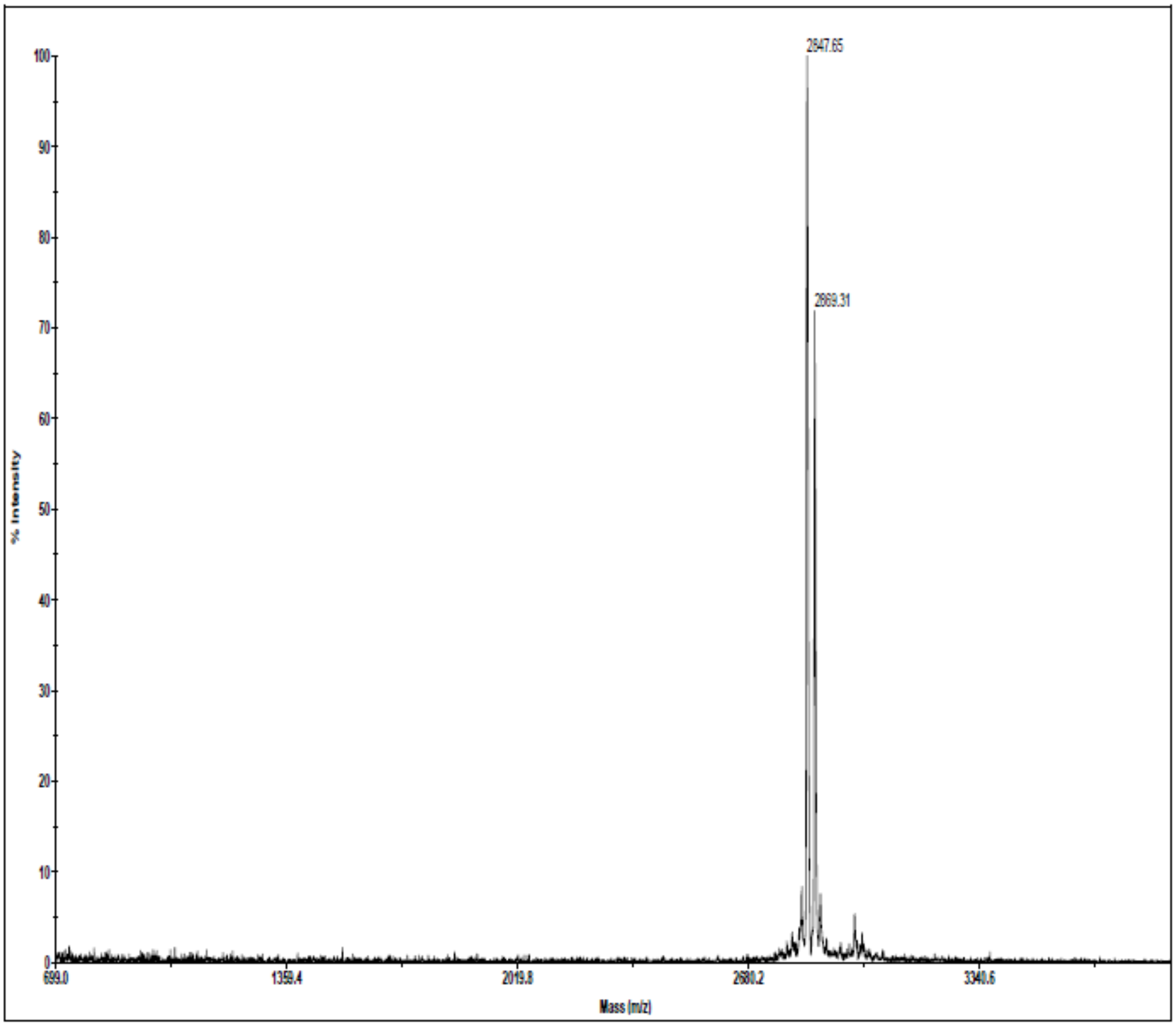

Lot No.: P300720-02-01
Method: MALDI-TOF
Main Peak: 2847.65
MW [M+H$^+$]: 2847.65
MW: 2846.65
Theoretical MW: 2846.53
Match: Approved
Z=1
